# Evaluation of Insulin-Like Activity of Novel Zinc Metal–Organics toward Adipogenesis Signaling

**DOI:** 10.3390/ijms22136757

**Published:** 2021-06-23

**Authors:** Catherine Gabriel, Olga Tsave, Maria P. Yavropoulou, Theodore Architektonidis, Catherine P. Raptopoulou, Vassilis Psycharis, Athanasios Salifoglou

**Affiliations:** 1 Laboratory of Inorganic Chemistry and Advanced Materials, School of Chemical Engineering, Aristotle University of Thessaloniki, 54124 Thessaloniki, Greece; katerinagabriel79@gmail.com (C.G.); tsaveolga@auth.gr (O.T.); h2tek42@outlook.com.gr (T.A.); 2 Center for Research of the Structure of Matter, Magnetic Resonance Laboratory, School of Chemical Engineering, Aristotle University of Thessaloniki, 54124 Thessaloniki, Greece; 3 Endocrinology Unit, 1st Department of Propaedeutic and Internal Medicine, Medical School, National and Kapodistrian University of Athens, Goudi, 11527 Athens, Greece; myavropoulou@med.uoa.gr; 4 Institute of Nanoscience and Nanotechnology, NCSR “Demokritos”, Aghia Paraskevi, 15310 Attiki, Greece; v.psycharis@inn.demokritos.gr

**Keywords:** zinc-induced adipogenesis, cell differentiation, insulin-like properties, zinc cell signaling, zinc metallodrugs, metal–organic complex, synthesis, crystal structure

## Abstract

Diabetes mellitus is a debilitating disease, plaguing a significant number of people around the globe. Attempts to develop new drugs on well-defined atoxic metalloforms, which are capable of influencing fundamental cellular processes overcoming insulin resistance, has triggered an upsurge in molecular research linked to zinc metallodrugs. To that end, meticulous efforts were launched toward the design and synthesis of materials with insulin mimetic potential. Henceforth, trigonelline and N-(2-hydroxyethyl)-iminodiacetic acid (HeidaH_2_) were selected as organic substrates seeking binding to zinc (Zn(II)), with new crystalline compounds characterized by elemental analysis, FT-IR, X-rays, thermogravimetry (TGA), luminescence, NMR, and ESI-MS spectrometry. Physicochemical characterization was followed by in vitro biochemical experiments, in which three out of the five zinc compounds emerged as atoxic, exhibiting bio-activity profiles reflecting enhanced adipogenic potential. Concurrently, well-defined qualitative–quantitative experiments provided links to genetic loci responsible for the observed effects, thereby unraveling their key involvement in signaling pathways in adipocyte tissue and insulin mimetic behavior. The collective results (a) signify the quintessential role of molecular studies in unearthing unknown facets of pathophysiological events in diabetes mellitus II, (b) reflect the close associations of properly configured molecular zincoforms to well-defined biological profiles, and (c) set the stage for further physicochemical-based development of efficient zinc antidiabetic metallodrugs.

## 1. Introduction

Diabetes mellitus is a galloping form of disease, with grievous repercussions on the quality of human life. The current American Diabetes Association (ADA) guidelines classify diabetes mellitus in two main subtypes: type 1 diabetes, which occurs due to autoimmune β-cell destruction, leading to absolute insulin deficiency, and type 2 diabetes occurring as a result of a progressive loss of adequate β-cell insulin secretion frequently on the background of insulin resistance [1]. A common factor in both of the above cases is insulin, which is a hormone with a plethora of physiological roles yet one that holds the key to pathophysiological aberrations inflicting serious damage upon organ tissues and processes, ultimately endangering human life [2,3]. 

**Scheme 1:**
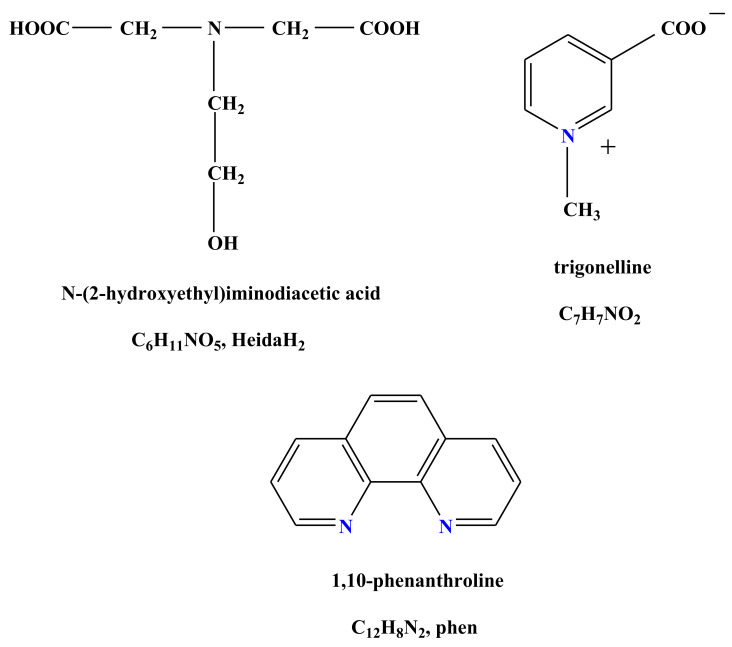
Organic substrates coordinating Zn(II).

The therapeutic administration of diabetes mellitus II has been actively pursued through an arsenal of drugs and drug formulations, most of them with insufficient potency or toxicity issues extensively brought upon patients [4]. To that end, efforts have been launched over the past decades to develop alternative drugs and therapeutic approaches with (a) more effective biological and clinical signatures and (b) fewer side effects than currently administered drugs, including insulin injections [5]. Paramount in that quest for new drugs has been the introduction of metal ions capable of exerting insulin mimetic effects. In such efforts fighting hyperglycemia through metal-induced insulin mimetic reactivity, several metal ions such as vanadium and zinc have been brought to the forefront of molecular drug development [6,7,8]. However, regardless of the extent of the work done, well-defined molecular drugs with specified effects on diabetes pathology, targeting specific processes, such as insulin resistance, have been limited. It is in this respect that research has been launched in our lab to pursue the design, synthesis, physicochemical characterization, and employment of well-defined binary and ternary zinc complex forms that (a) possess solubility and bioavailability profiles, enabling further use in biological investigative work, and (b) intercede molecular reactivity pathways, uniquely or synergically (with insulin) counteracting resistance mechanisms inherent to diabetes mellitus II [9,10]. Key to such research and thus the emergence of zincoforms, acting on insulin resistance pathways, is the use of appropriately designed organic ligands that will coordinate Zn(II) and in so doing modulate its chemical reactivity in biologically relevant media. In line with such a rationale, carboxylic acids were selected as a general family of organic substrates to bestow new coordination properties upon Zn(II), which is a non-redox active metal ion with rich chemical reactivity history. In that family of ligands, (a) a zwitterionic substrate was chosen that contains the necessary carboxylic acid (negative terminal) moiety anchored onto an aromatic ring and a positively charged terminal in a meta-position linked to the pyridine nitrogen atom of the same ring. The specific substrate (trigonelline) was selected, as it reflects a natural derivative of vitamin B_3_ and concurrently exemplifies an important methylation process on the nitrogen of the molecule; and (b) a hydroxy dicarboxylic amino acid, i.e., N-(2-hydroxyethyl)iminodiacetic acid (HeidaH_2_), was chosen that contains a carboxylic acid group, an alcoholic terminal, and an imino nitrogen anchor, which are all potential Lewis acid bases seeking metal ion coordination, including chelation [11]. The rationale for choosing the specific ligands was supplemented by the introduction of an N,N’-aromatic chelator 1,10 phenanthroline (phen), which is known to efficiently bind metal ions and thermodynamically stabilize their emerging coordination sphere. Collectively, the selection of the aforementioned ligands (Scheme 1) provides the necessary grounds for the development of discretely defined binary and ternary complex materials, further entering biological studies in an effort to evaluate their potential insulin mimetic effect potency in cell differentiation (adipogenesis) and their impact on insulin signaling.

## 2. Results

### 2.1. Synthesis

**Scheme 2:**
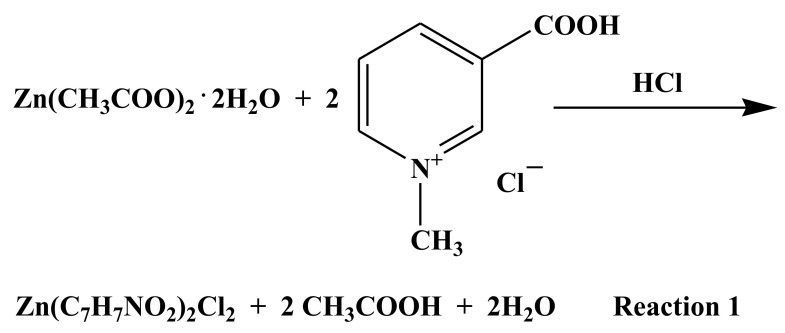
Reaction scheme for the synthesis of compound **1**.

The synthesis of compound Zn(C_7_H_7_NO_2_)_2_Cl_2_ (**1**) was achieved efficiently through a reaction between Zn(II) and trigonelline hydrochloride in aqueous solutions. The pH, at which the reaction was developed, was 7.0. The adjustment of pH was made by adding a solution of ammonia (1:1). In the final product, the presence of chlorides bound to Zn(II) was evident. This behavior had been previously observed in zinc chemistry [12,13,14,15,16,17,18]. The stoichiometric reaction, leading to the formation of the title compound, is shown below (Scheme 2, Reaction 1).

**Scheme 3:**
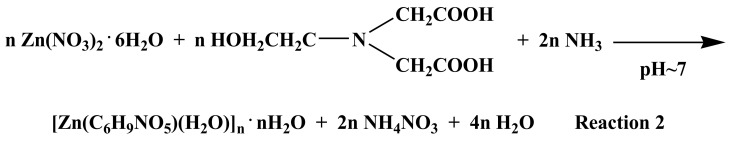
Reaction scheme for the synthesis of compound **2**.

The synthesis of compound [Zn(C_6_H_9_NO_5_)(H_2_O)]n • nH2O (**2**) was achieved efficiently through a reaction between Zn(II) and N-(2-hydroxyethyl)iminodiacetic acid in aqueous solutions. The pH at which the reaction was developed was 7.0The adjustment of pH was made by adding a solution of ammonia (1:1).. The stoichiometric reaction, leading to the formation of the title compound, is shown below (Scheme 3, Reaction 2).

**Scheme 4:**
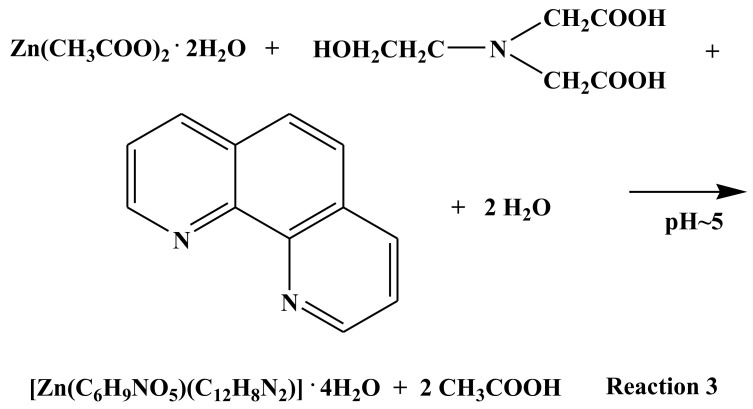
Reaction scheme for the synthesis of compound **3**.

Compound [Zn(C_6_H_9_NO_5_)(C_12_H_8_N_2_)]•4H_2_O (**3**) was synthesized expediently through a reaction between Zn(II) and N-(2-hydroxyethyl)iminodiacetic acid in aqueous solutions. The pH at which the reaction was developed was 5. Adjustment of the pH was made by the addition of 1,10-phenanthroline (phen). The stoichiometric reaction, leading to the formation of the title compound, is shown below (Scheme 4, Reaction 3).

**Scheme 5:**
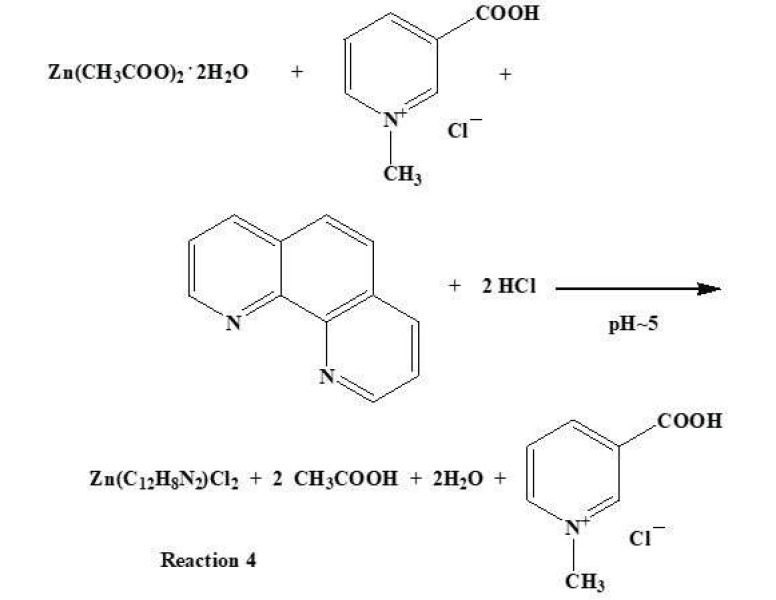
Reaction scheme for the synthesis of compound **4**.

The synthesis of compound Zn(C_12_H_8_N_2_)Cl_2_ (**4**) was expediently pursued through a facile reaction between Zn(II) and trigonelline hydrochloride in aqueous solutions at pH ≈ 5. Adjustment of pH was achieved by the addition of 1,10-phenanthroline. The stoichiometric reaction, leading to the formation of **4** (Scheme 5, Reaction 4), is shown below.

**Scheme 6:**
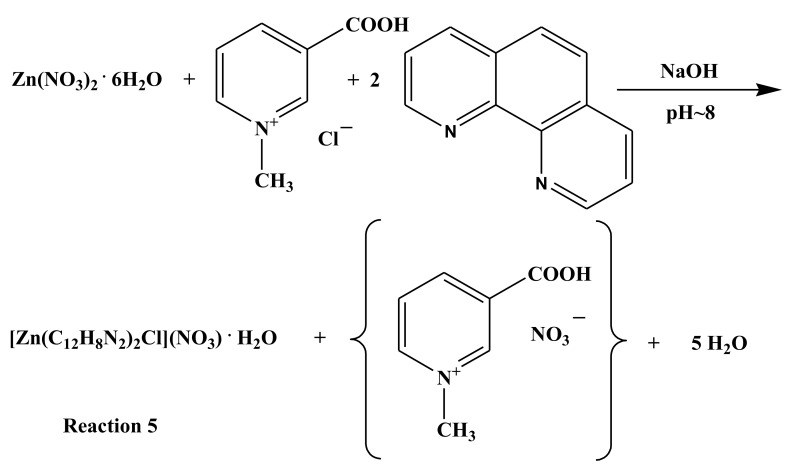
Reaction scheme for the synthesis of compound **5**.

The synthesis of compound [Zn(C_12_H_8_N_2_)_2_Cl](NO_3_)•H_2_O (**5**) was also pursued through a facile reaction between Zn(II) and trigonelline hydrochloride in aqueous solutions at pH ≈ 8. Adjustment of pH was achieved by the addition of 1,10-phenanthroline and sodium hydroxide (1.0 Μ). The stoichiometric reaction, leading to the formation of **5** (Scheme 6, Reaction 5), is shown below.

Compounds **1–5** were all isolated in pure crystalline form. Elemental analysis of the isolated crystalline materials suggested the molecular formulation Zn(trigonelline)_2_Cl_2_ (**1**), [Zn(Heida)(H_2_O)]•H_2_O (**2**), [Zn(Heida)(phen)]•4H_2_O (**3**), Zn(phen)Cl_2_ (**4**), and [Zn(phen)_2_Cl](NO_3_)•H_2_O (**5**). Further spectroscopic inspection of **1**-**5** by FT-IR revealed the presence of the ligands bound to Zn(II), thus supporting the proposed formulation. 

Compounds **1–5** are soluble in water. The materials are stable in the air at room temperature for a long period of time. 

### 2.2. Description of Crystallographic Structures

**Figure 1:**
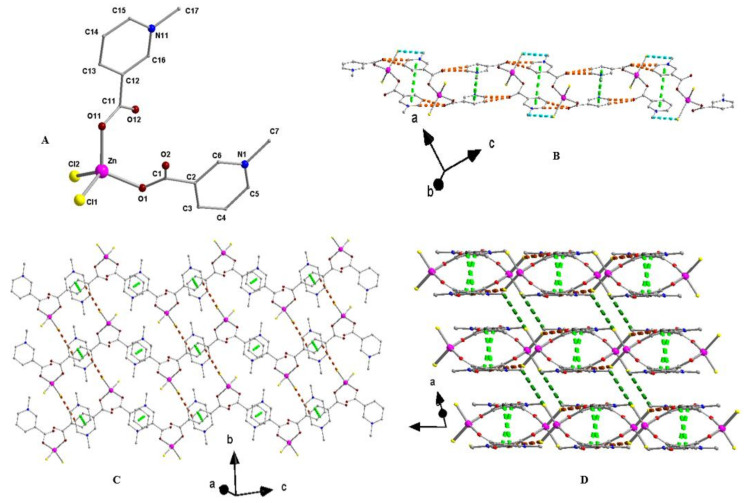
(**A**) Plot of the molecular structure of **1**. (**B**) Chains of clusters along the [10$\bar{1}$] direction. Dashed green, cyan, and orange lines indicate π–π, C-H^…^Cl, and C-H^…^O interactions. (**C**) Layers of clusters parallel to the (101) plane formed through C(14)-H(14)^…^Cl(1) hydrogen bonds (dashed brown lines). (**D**) The 3D lattice structure of compound **1** built through C(7)-H(7B)^…^Cl(2) hydrogen bonds (dashed dark green lines). In (**C**,**D**), hydrogen bonds along the chains are not shown for clarity reasons.

**Figure 2:**
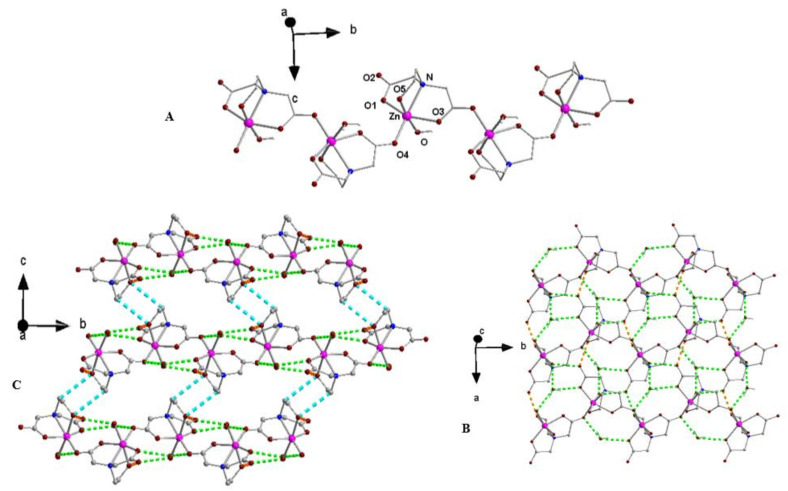
(**A**) Part of the 1D chains of **2** along the b-axis. (**B**) Part of the 2D structure of **2** due to O(5)-H(5O)^…^O(2) (orange dashed lines) hydrogen bonds and hydrogen bonds developing among coordinated, lattice solvent water molecules, and coordinated oxygen atoms as well (green dashed lines). (**C**) Part of the 3D lattice structure of **2** due to C-H^…^O hydrogen bonds (cyan dashed lines).

**Figure 3:**
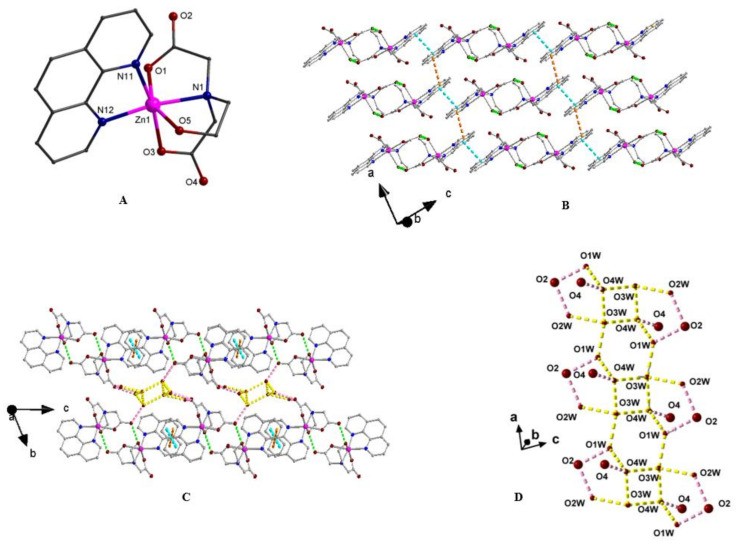
(**A**) Partially labeled plot of the molecular structure of **3** (**B**) Layers of the complex forming through O-H···O hydrogen bonds (intradimer interactions indicated by green dashed lines) and π-π type of interactions, with the two types of overlap indicated by cyan and orange lines, respectively. (**C**) 3D structure of **3** built by layers of complexes and tapes of water. (**D**) Tape of waters linking the layers of complexes. Pink and yellow dashed lines indicate intermolecular interactions among water and the complexes and intra-tape hydrogen bond interactions.

**Figure 4:**
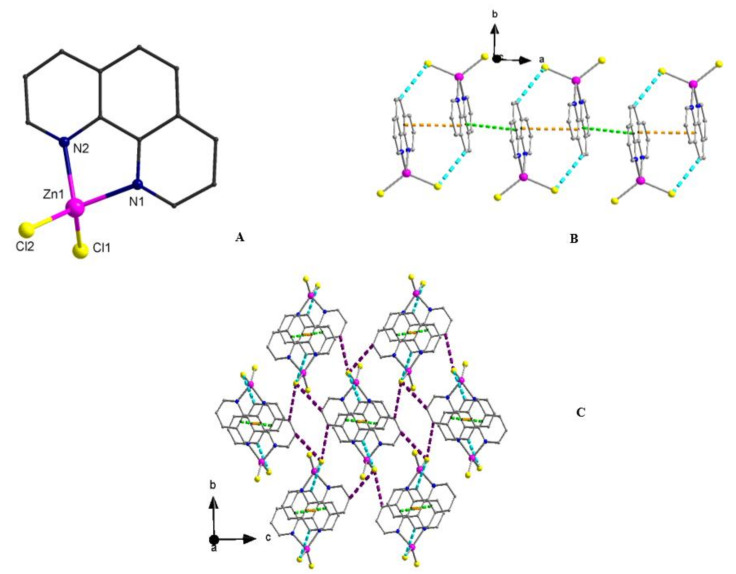
(**A**) Partially labeled plot of the molecular structure of **4**. (**B**) Chains of complexes along the a-axis formed through C-H∙∙∙Cl hydrogen bonds (cyan dashed lines) and π–π type of interactions (intradimer and interdimer interactions are indicated with dashed orange and light green lines, respectively). (**C**) The 3D intermolecular structure of **4** due to C-H^…^Cl interactions (dashed violet lines).

**Figure 5:**
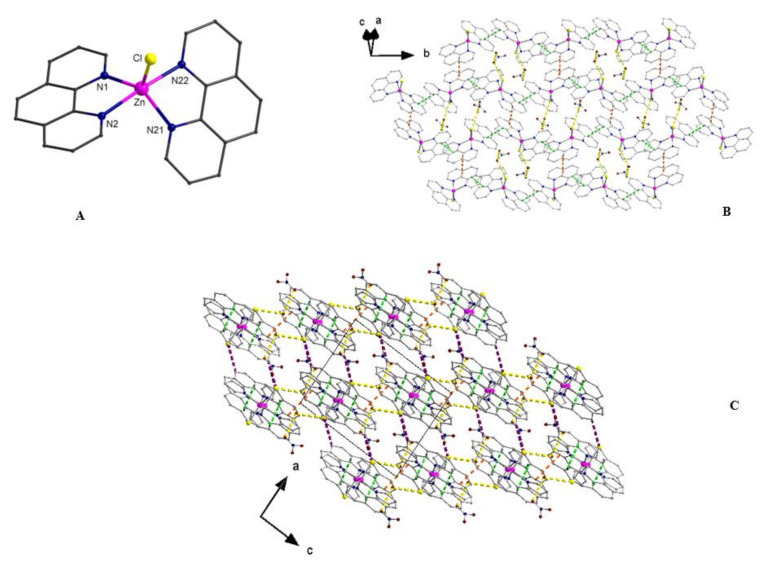
(**A**) Partially labeled plot of the molecular structure of **5**. (**B**) Part of the 2D intermolecular Scheme 5 due to π-π interactions (dashed green lines and orange ones indicate intrachain and interchain interactions, respectively). (**C**) Part of the 3D intermolecular structure of **5** (dashed violet line indicate C(6)-H(6)^…^Cl interactions). Dashed yellow lines indicate O(1w)-H(1w1)^…^Cl and O(1w)-H(2w1)^…^O(32) hydrogen bonds. C(3)-H(3)^…^Cl and C(23)-H(23)^…^Cl intermolecular interactions are not shown for clarity reasons.

The X-ray crystal structures of **1**–**5** reveal discrete solid-state lattices. The molecular structures of **1**–**5** are provided in Figure 1, Figure 2, Figure 3, Figure 4 and Figure 5; crystallographic data details are provided in Table 1, with selected bond distances and angles listed in Table 2. Compound **1** crystallizes in the triclinic space group *P*$\bar{1}$. The solid-state structure of **1** reveals a mononuclear species. The Zn(II) center is coordinated to two trigonelline ligands and two chloride ions. Zn(II) is four-coordinate, with two of the four places covered by the carboxylato oxygens of the trigonelline ligand and the other two occupied by two chloride ions (Figure 1A). The coordination geometry around the Zn(II) ion is tetrahedral with angles in the range 104.54(8)–113.52(10) °. The mononuclear entities in the lattice of **1** are linked through π-π intermolecular interactions, which develop between centrosymmetrically related pyridine rings of both trigonelline ligands. In so doing, they create chains extending parallel to the [(10$\bar{1}$)] crystallographic direction (intercentroid distances between the parallel pyridine rings, C_g1_···C_g1_ = 3.468 Å, C_g2_···C_g2_ = 3.531 Å; Cg1 and Cg2 correspond to centroids of pyridine rings defined by N(1) and N(11), respectively). Neighboring clusters along the chain direction are further linked through the C(17)-H(17A)···Cl(1), C(4)-H(4)···O(12), C(5)-H(5)···O(12), C(14)-H(14)···O(2) and C(15)-H(15)···O(2) hydrogen bonds (Table S1, Figure 1B). The chains interact through C(14)-H(14)···Cl(1) hydrogen bonds and form layers parallel to the (101) plane (Table S1, Figure 1C). Monomers belonging to neighboring layers interact through C(7)–H(7B)···Cl(12) hydrogen bonds, thus giving rise to a 3D architecture (Figure 1D).

Compound **2** crystallizes in the monoclinic space group P2_1/c_. The structure consists of polymeric chains forming by Zn(II) metal centers connected through N-(2-hydroxyethyl)iminodiacetato ligands (Figure 2A). The Zn(II) ion is octahedrally coordinated, with one site occupied by the imino nitrogen atom, a second site occupied by the terminal alcoholic oxygen atom O(5), and two others occupied by carboxylato oxygen atoms O(1) and O(3). The fifth coordination site is occupied by a water ligand. The two carboxylate groups adopt two different coordination modes: a monodentate one through O(1) and a *syn,anti*-bridging one through O(3) and O(4) oxygen atoms. O(4) atoms complete the octahedral distorted geometry of the Zn(II) metal centers, with the specific atom serving as an anchor to a neighboring Zn(II) ion, thus forming polymeric chains, which extend parallel to the *b* crystallographic axis (Figure 2A). A water solvent molecule is also present in the asymmetric unit of the structure. The chains of **2** are linked through strong hydrogen bonds O(5)-H(5O···O(2) and form a 2D network, which extends parallel to the (001) crystallographic plane (Figure 2B, Table S1). The chains within the layers are further linked via hydrogen bonds through the coordinated and lattice solvent water molecules (Figure 2B, Table S1). The lattice structure of **2** further evolves into a 3D network due to C(5)-H(5B)···O(5) hydrogen bonds (Figure 2C).

Compound **3** crystallizes in the triclinic space group *P*$\bar{1}$. The molecular structure of **3** consists of mononuclear Zn(II) complexes and four lattice water molecules per monomer. In each mononuclear unit, the Zn(II) ion is coordinated to a phen molecule through the two nitrogen atoms and to a N-(2-hydroxyethyl)iminodiacetato dianion, which is bound via the imino nitrogen N(1), the protonated alcoholic oxygen atom O(5), and the two deprotonated carboxylato groups through O(1) and O(3). The coordination geometry around the Zn(II) ion is distorted octahedral (Figure 3A). The mononuclear entities of **3** form dimers through hydrogen bonds, developing between the protonated alcoholic oxygen O(5) and one of the non-coordinated carboxylato oxygen atoms O(4), belonging to a centrosymmetrically related mononuclear molecule (Table S1). Neighboring, centrosymmetrically related dimers are linked through π-π interactions of aromatic rings of the phen coordinated molecules. The planes of phen ligands stand at 3.141(7) and 3.246(6) Å apart, revealing two types of overlap. In so doing, they form layers parallel to the (010) plane (Figure 3B). Neighboring layers interact along the b crystallographic axis through the lattice water molecules, thus giving rise to a 3D architecture of the structure (Figure 3C). The lattice water molecules O(1w), O(3w), and O(4w) form tapes of the T4(2)6(2) type, through hydrogen bonds, with the four-membered rings being planar and the six-membered one exhibiting a chair conformation, respectively (Figure 3D, Table S1) [19]. These tapes of water molecules are linked through the water lattice molecule O(2w) “(O(2w)^…^O(3w) hydrogen bond) and the O(2w)^…^O(2), O(1w)^…^O(2), and O(4w)^…^O(4) hydrogen bonds, with the mononuclear complexes forming the layers.

Compound **4** crystallizes in the monoclinic space group P2_1_/n. The molecular structure of **4** has been determined previously from precession photographs taken with Mo Kα radiation at room temperature. However, our data are unambiguously of better quality and precision [20]. The molecular structure of **4** consists of mononuclear complexes. The Zn(II) ion is coordinated to two Cl^−^ anions and one phen molecule, thus exhibiting a tetrahedral N_2_Cl_2_ coordination environment (Figure 4A). The bond angles around the tetrahedral Zn(II) ion are in the range 81.49(8)–125.83(6) °; the smallest angle is the bite angle between the nitrogen atoms of phen. The mononuclear entities of **4** are linked through π-π intermolecular interactions, which develop between centrosymmetrically related aromatic rings of phen ligands and form dimers with their planes standing 3.486(2) Å apart. There is an additional intradimer C(5)-H(5)∙∙∙Cl(1) hydrogen bond interaction (Figure 4B). Phen molecules belonging to neighboring dimers interact through π–π interactions, and their plains are at a distance of 3.544(1) Å. The dimers are stacked along chains parallel to the *a* crystallographic axis (Figure 4B). The chains are further linked through C-H···Cl intermolecular interactions and build the 3D lattice structure of **4** (Table S1, Figure 4C).

Compound **5** crystallizes in the monoclinic space group P2_1_/n and consists of mononuclear assemblies. The Zn(II) ion is coordinated to four nitrogen atoms from two phen ligands and one Cl^−^ ion (Figure 5A). The coordination geometry around the Zn(II) ion is trigonal bipyramidal, with the apical positions defined by atoms N(2) and N(22) (N(2)-Zn-N(22) = 172.72(7) °) and the trigonal plane defined by the remaining atoms, i.e., N(1), N(21), Cl. The bond angles in the trigonal plane are in the range 118.31(5)-121.09(7) °. The trigonality index is τ = 0.86 (τ = 0 for square pyramid and τ = 1 for trigonal bipyramid). The mononuclear units of **5** are linked through π-π intermolecular interactions, developing between aromatic rings of phen ligands, defined by N1, N2, and N21ˊ, N22ˊ atoms (symmetry code (ˊ): 0.5-x, 0.5+y, 0.5-z) and which are related by a two-fold screw axis symmetry, with the angle and the distance between the planes being 3.94(3)° and 3.453(2) Å, respectively. The so-formulated architecture gives rise to chains parallel to the b crystallographic axis. Mononuclear units belonging to neighboring chains also interact through π-π interactions of centrosymmetrically related phen ligands, which are defined by N(21), N(22), and N(21ˊˊ), N(22ˊˊ) atoms (symmetry code (ˊ): 1-x, -y, 1-z), with the distance of the parallel planes being 3.380(3) Å and forming layers parallel to the [(10$\bar{1}$)] planes. Monomers, within the layers, interact further through C(3)-H(3)···Cl and C(23)-H(23)…Cl intermolecular interactions. The lattice water molecule and NO_3_^−^ anion are linked to the layers of monomers through the O(1w)-H(1w1)···Cl and O(1w)-H(2w1)···O(32) hydrogen bonds (Table S1). The mononuclear assemblies interact through the C(6)-H(6)···Cl intermolecular bonds, thus giving rise to the 3D lattice structure of 5 (Figure 5A–C, Table S1). The molecular structure of **5** has been reported previously, albeit as a complex [ZnCl(phen)_2_](NO_3_)•H_2_O crystallized in the triclinic space group *P*$\bar{1}$ It consists of mononuclear units, forming chains similar to the one observed in **5** but with different packing, thus resulting in a different lattice structure [21].

### 2.3. ESI-MS Spectrometry

The spectra of **1**–**5** confirm their identity in solution, with the ESI-MS measurements run in positive mode, on solutions arising upon dissolution of the title compounds in water.. For **1**, M_1-1_ = [Zn(L_1_H)OH_2_], (*m*/*z* = 235.9442, z = 1, [M_1-1_H]^+^), M_1-2_ = [Zn(L_1_H)_2_OH_2_], (*m*/*z* = 372.9917, z = 1, [M_1-2_H]^+^), (Figure S1-A); for **2**, M_2-1_ = [Zn(L_2_)], (*m*/*z* = 237.9703, z = 1, [M_2-1_H]^+^), M_2-2_ = [Zn(L_2_)_2_H_2_O], (*m*/*z* = 255.2331, z = 1, [M_2-1_H]^+^), (Figure S1-B); for **3**, M_3-1_ = [Zn(phen)(OH)_2_], (*m*/*z* = 303.0109, z = 1, [M_3-1_H]^+^), M_3-2_ = [Zn(L_2_)(phen)], (*m*/*z* = 420.0539, z = 1, [M_3-2_H]^+^), (Figure S1-C); for **4**, M_4-1_ = [Zn(phen)(OH)_2_], (*m*/*z* = 303.0109, z = 1, [M_4-1_H]^+^), M_4-2_ = [Zn(L_2_)(phen)], (*m*/*z* = 420.0539, z = 1, [M_4-2_H]^+^), (Figure S1-D); and for **5**, M_5-1_ = [Zn(phen)(Cl)], (*m*/*z* = 278.9657, z = 1, [M_5-1_H]^+^) (Figure S1-E).

### 2.4. Solution NMR Spectroscopy

#### 2.4.1. ^13^C-NMR Spectroscopy

**Figure 6:**
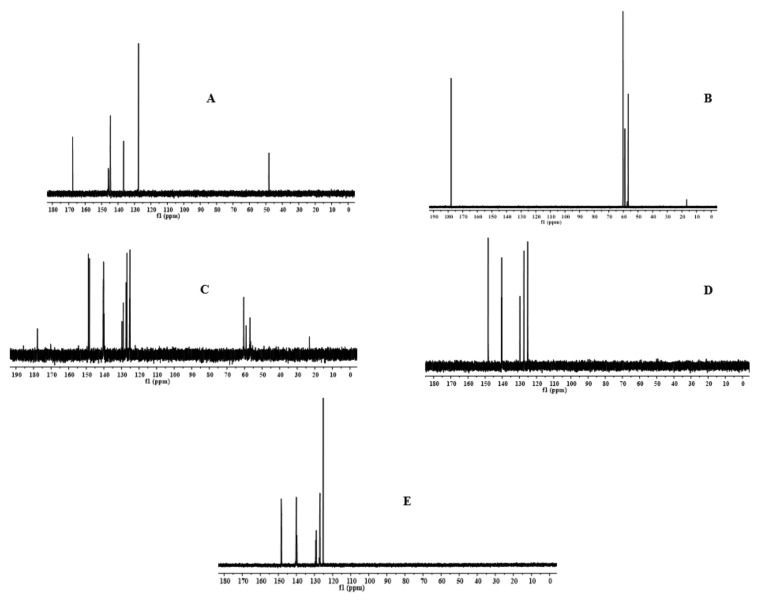
^13^C-Solution NMR spectra of **A(1), B(2), C(3), D(4), and E(5)** in D_2_O.

The solution ^13^C-NMR spectra of freshly prepared compounds **1**–**5** were measured in D_2_O. The ^13^C spectrum of **1** (Figure 6A, Supplementary Materials Figure S2A) reveals a resonance at 48.8 ppm, which is attributed to the –CH_3_ groups. Resonances between 127.7 and 144.5 ppm are assigned to the aromatic carbons of the trigonelline ligand, and a resonance at 167.8 ppm is attributed to the –COO^–^ carbon of the bound trigonelline ligand. The spectrum of **2** (Figure 6B, Supplementary Materials Figure S2B) exhibits three resonances at 57.9, 59.0, and 60.41 ppm (-CH_2_- groups) and one resonance at 177.68 ppm (–COO^–^ carbons) of the HEIDA^2−^ ligand bound to Zn(II). The spectrum of **3** (Figure 6C, Supplementary Materials Figure S2C) exhibits three resonances at 56.7, 59.9, and 60.1 ppm (-CH_2_- groups) and one resonance at 177.68 ppm (–COO^–^ carbons) for the HEIDA^2−^ ligand bound to Zn(II). Furthermore, there are several signals between 125.3 and 159.2 ppm, which are assigned to phen bound to Zn(II). All resonances are shifted to slightly higher values compared to the free ligand. Figure 6D (Supplementary Materials Figure S2D) presents the spectrum of **4**, with signals between 125.3 and 148.3 ppm representing carbons of the phen ligand. Finally, Figure 6E (Supplementary Materials Figure S2E) exhibits the spectrum of **5**, with signals between 125.7 and 148.5 ppm representing carbons of the phen ligand. The above observations are consistent with the X-ray crystallographic results.

#### 2.4.2. ^1^H-NMR Solution NMR

The ^1^H-NMR spectra of **1**–**5** in D_2_O showed several peaks. The spectrum of **1** (Supplementary Materials Figure S3A) exhibits a resonance at 4.3 ppm, which is consistent with the presence of the methyl protons. The resonances at 8.9 and 9.2 ppm are attributed to the aromatic protons. The spectrum of **2** (Supplementary Materials Figure S3B) exhibits resonances in the range 2.7–3.4 ppm that are consistent with the presence of the–N-CH_2_–, the –CH_2_COO^−^, and the –CH_2_OH group protons. The spectrum of **3** (Supplementary Materials Figure S3C) exhibits resonances in the range 2.7–3.6 ppm that are consistent with the presence of the –N–CH_2_–, the –CH_2_COO^−^, and the –CH_2_OH group protons. The resonances in the range 7.6–8.9 ppm could be attributed to the presence of aromatic protons. The spectrum of **4** (Supplementary Materials Figure S3D) exhibits resonances in the range 7.5–8.5 ppm that could be attributed to the presence of aromatic protons. The spectrum of **5** (Supplementary Materials Figure S3E) exhibits resonances in the range 7.6–8.9 ppm that could be attributed to the presence of aromatic protons.

### 2.5. Thermal Studies

**Scheme 7:**
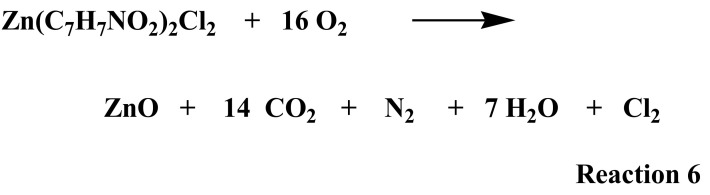
Thermogravimetric reactivity in **1**.

**Scheme 8:**
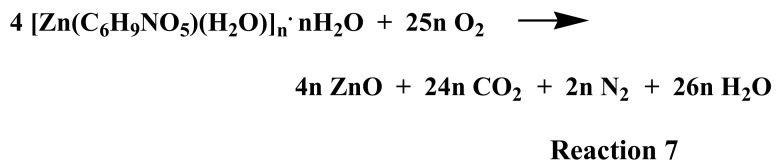
Thermogravimetric reactivity in **2**.

**Scheme 9:**
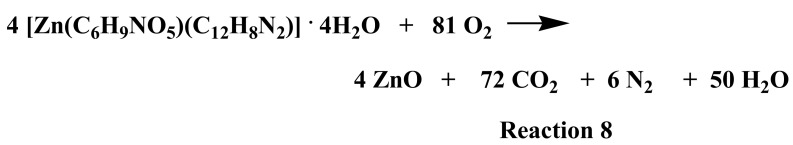
Thermogravimetric reactivity in **3**.

The thermal behavior of **1**–**5** was examined by TGA under an oxygen atmosphere. Compound **1** is thermally stable up to 291 °C (Figure S4A). Beyond that point, there are sequential processes up to 620 °C for **1**, involving decomposition of the organic part of the material, which is consistent with the reactivity shown below (Scheme 7, reaction 6):

In **2**, there are two steps, where lattice water molecules and crystal water molecules are removed and there are two sequential processes in the range 100–438 °C, reflecting decomposition of the organic part of the material (Figure S4B), which is consistent with the reactivity shown below. No clear plateaus are reached between processes, thereby suggesting instability of the derived intermediates and further decomposition, which is consistent with the reactivity shown below (Scheme 8, reaction 7): 

In **3**, there is one step, where lattice water molecules are removed and there are two sequential processes in the range 107–471 °C, reflecting decomposition of the organic part of the material (Figure S4C), which is in line with the reactivity shown below (Scheme 9, reaction 8). No clear plateaus are reached between processes, thereby suggesting instability of the derived intermediates and further decomposition. 

**Scheme 10:**
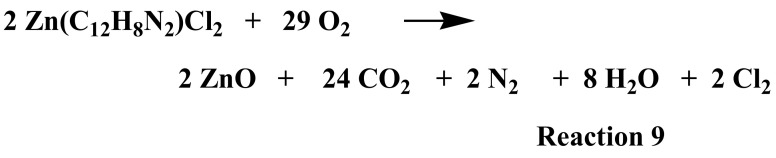
Thermogravimetric reactivity in **4**.

Compound **4** retains its stability up to 405 °C. After that, there are two sequential processes in the range 405–654 °C, projecting decomposition of the organic part of the material (Figure S4D). The aforementioned processes involve no clear plateaus, projecting instability and further decomposition of the derived products, which is in line with the reactivity shown below (Scheme 10, reaction 9). 

**Scheme 11:**
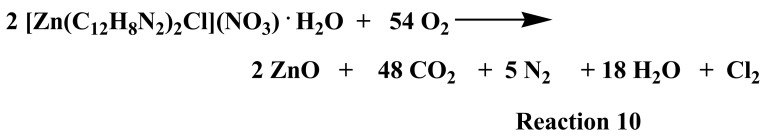
Thermogravimetric reactivity in **5**.

Compound **5** maintains stability up to 55 °C. Beyond that point, there are several process steps in the range 55–635 °C, reflecting decomposition of the organic part of the title compound (Figure S4E), which is in line with the reactivity shown below (Scheme 11, reaction 10): 

Mass loss calculations are in line with ZnO being the final product for all compounds. Decomposition reaches completion at 620 °C for **1**, 438 °C for **2**, 471 °C for **3**, 654 °C for **4**, and 635 °C for **5**, with the total mass loss standing at 95% for **1**, 72% for **2**, 84% for **3**, 95% for **4**, and 92% for **5**. 

### 2.6. Luminescence

The solid-state spectra of **1**–**5**, along with those of trigonelline, HeidaH_2_, and phen ligands were studied at room temperature (Figure S5A–E). The spectral data suggest that none of the free ligands trigonelline or HeidaH_2_ possess any luminescence. The strongest emission band for free phen appears at 417 nm (λ**_ex_** 368 nm) and could be assigned to π*–π transitions. The emission spectrum of **1** exhibits a strong peak at 390 nm (λ**_ex_** 339 nm), with **2** displaying an intense band at 387 nm (λ**_ex_** 300 nm). Compound **3** shows a strong emission at 393 nm (λ**_ex_** 352 nm). In **4** and **5**, too, two emissions emerge at 379 and 398 nm (λ**_ex_** 351 nm) for **4**, and there is a strong emission at 449 nm (λ**_ex_** 382 nm) for **5**. These emissions are neither metal-to-ligand charge transfer (MLCT) nor ligand-to-metal transfer (LMCT) in nature, since the Zn(II) ion is difficult to oxidize or reduce due to its d^10^ configuration [22]. They could potentially be assigned to the intraligand (π–π*) fluorescent emission. Many aromatic ligands, such as phenanthroline ligands, which possess a fluorescence profile yet they are not strongly emissive on their own, show much stronger luminescence when coordinated to Zn(II) [23]. The observed enhancements in luminescence are likely a result of coordination of those ligands to Zn(II) [24]

### 2.7. Biological Studies

#### 2.7.1. Cytotoxicity Results

##### Zn(II) Compound Toxicity in 3T3-L1 Pre-Adipocytes for 24 h

**Figure 7:**
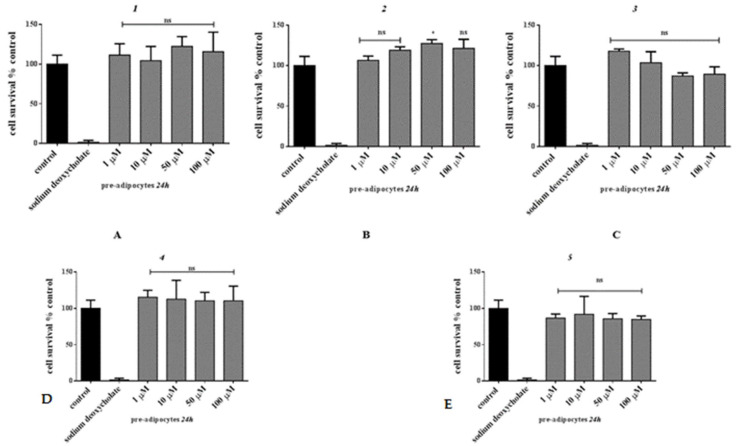
Percent change of cell survival in 3T3-L1 pre-adipocytes, following treatment with various concentrations (0.5–100 μM) of **1**(**A**)–**5**(**E**) for 24 h. Sodium deoxycholate has been used as a positive control. Values represent the mean value of several (n = 3) independent experiments. Vertical bars represent SEMs. *: *p* 0.05 vs. control.

To assess the effect of **1**–**5** on cell viability, 3T3-L1 pre-adipocytes were exposed to the title complexes at various concentrations (1–100 μΜ) for 24 h. Sodium deoxycholate (1 mg/mL) was used as a positive control of the assay, exhibiting full reduction of cell survival under the employed experimental conditions. More specifically, **1** and **4** were completely atoxic for the tested concentrations under the employed experimental conditions (Figure 7). Complex **2** exhibits a slight proliferative effect at higher concentrations (50 and 100 μΜ), which amounts to ≈20% compared to the control (untreated cells) (Figure 7B). Complex **3** is completely atoxic at low concentrations (1 and 10 μΜ), whereas a slight concentration-dependent reduction is observed at higher concentrations, reaching up to 12% and 11% when cells are treated with 50 and 100 μΜ, respectively (Figure 7C). Complex **5** projects a slight reduction in cell survival (≈12.7%) in all cases (Figure 7E).

##### Zn(II) Compound Toxicity in 3T3-L1 Pre-Adipocytes for 48 h

**Figure 8:**
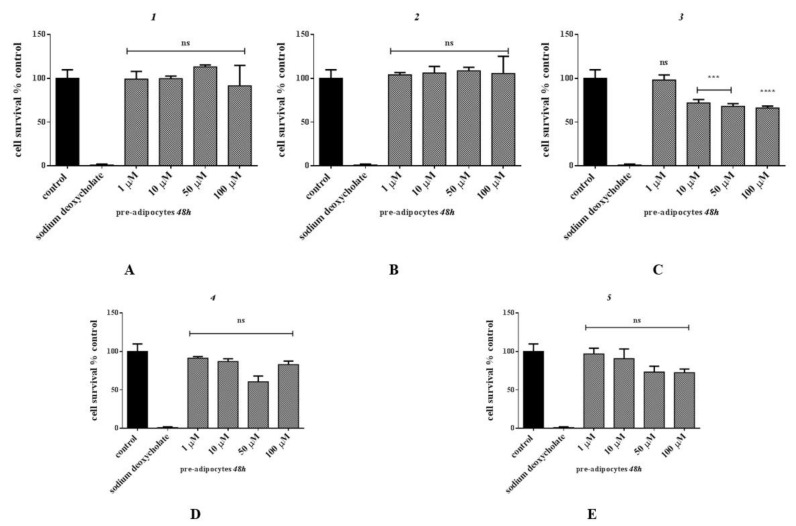
Cell survival in 3T3-L1 pre-adipocytes following treatment with various concentrations (0.5–100 μM) of **1**(**A**)–**5**(**E**) for 48 h. Sodium deoxycholate has been used as a positive control. Values represent the mean value of several (n = 3) independent experiments. Vertical bars represent SEMs. *** *p* 0.001 (extremely significant), and **** *p* ≤ 0.0001 (extremely significant) or non-significant (*p* 0.05).

In similar experiments, 3T3-L1 pre-adipocytes were exposed to the same concentration range (1–100 μΜ) for a longer incubation period (48 h). The specific time period was selected since cells are incubated longer than 24 h in the presence of the title complexes during the induction and maintenance of the adipogenesis process. Complex **1** appears to be completely atoxic at concentrations up to 50 μΜ, whereas a slight reduction in cell survival is observed (≈10%) at 100 μΜ (Figure 8A). Complex **2** appears to be completely atoxic at all concentrations tested under the employed experimental conditions (Figure 8B). Complex **3** appears to be atoxic at low concentrations (1 μΜ), whereas a slight concentration-dependent reduction in cell survival is observed when cells are treated with 10, 50, and 100 μΜ, which amounts to 72%, 68.1%, and 66.3%, respectively (Figure 8C). 

In the case of **4**, cell survival amounts to 91.3%, 87.0%, 60.7%, and 83.0%, when cells are treated with 1, 10, 50, and 100 μΜ, respectively, projecting a concentration-dependent reduction in cell survival (Figure 8D). Similarly, complex **5** reduces cell viability in a concentration-dependent manner. More specifically, cell survival amounts to 96.9%, 90.6%, 73.2%, and 72.5%, when cells are treated with 1, 10, 50, and 100 μΜ, respectively (Figure 8E).

##### Zn(II) Compound Toxicity in Differentiated 3T3-L1 Cells for 48 h

**Figure 9:**
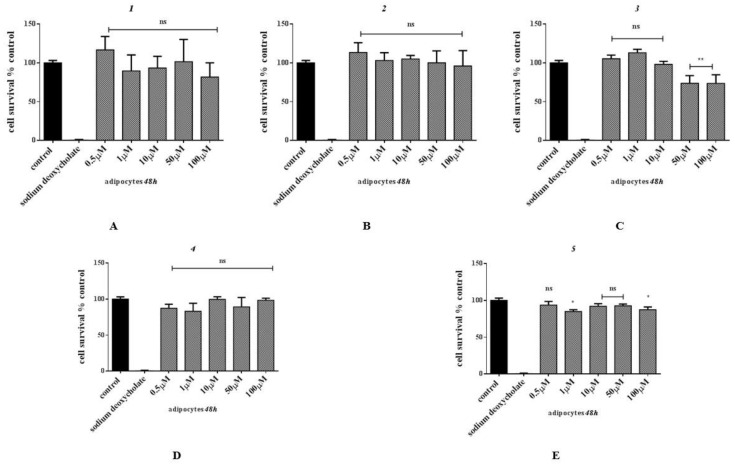
Percent change of cell survival in 3T3-L1 mature adipocytes following treatment with various concentrations (0.5–100 μM) of **1**(**A**)–**5**(**E**) for 48 h. Sodium deoxycholate has been used as a positive control. Values represent the mean value of several (n = 3) independent experiments. Vertical bars represent SEMs. * *p* 0.05 (significant), ** *p* 0.01 (highly significant), or non-significant (*p* 0.05).

Finally, cell survival rates were also assessed in the case of mature adipocytes (differentiated in the presence of insulin). Fully differentiated adipocytes were exposed to the title complexes at various concentrations (0.5–100 μΜ) for 48 h. Here, too, sodium deoxycholate was used as a positive control of the assay, exhibiting full reduction of cell survival under the employed experimental conditions. Complex **1** appears to be completely atoxic at concentrations ranging from 0.5 to 50 μΜ, whereas reduction in cell survival is observed (≈20%) when cells are treated with 100 μΜ (Figure 9A). Complex **2** appears to be completely atoxic (Figure 9B), whereas **3** is also completely atoxic at concentrations up to 10 μΜ. In the case of **3**, cell viability is reduced by ≈26%, when cells are treated with higher concentrations (50 and 100 μΜ) (Figure 9C). 

In the case of **4**, cell survival amounts to 87.4%, 83.1%, 99.7%, 89.2%, and 98.4%, when cells are treated with 0.5, 1, 10, 50, and 100 μΜ, respectively (Figure 9D). Similarly, in the case of **5**, cell survival amounts to 93.8, 84.9%, 92.0%, 92.7%, and 87.4%, when cells are treated with 0.5, 1, 10, 50, and 100 μΜ, respectively (Figure 9E). 

#### 2.7.2. Oil Red O Staining Results

**Figure 10:**
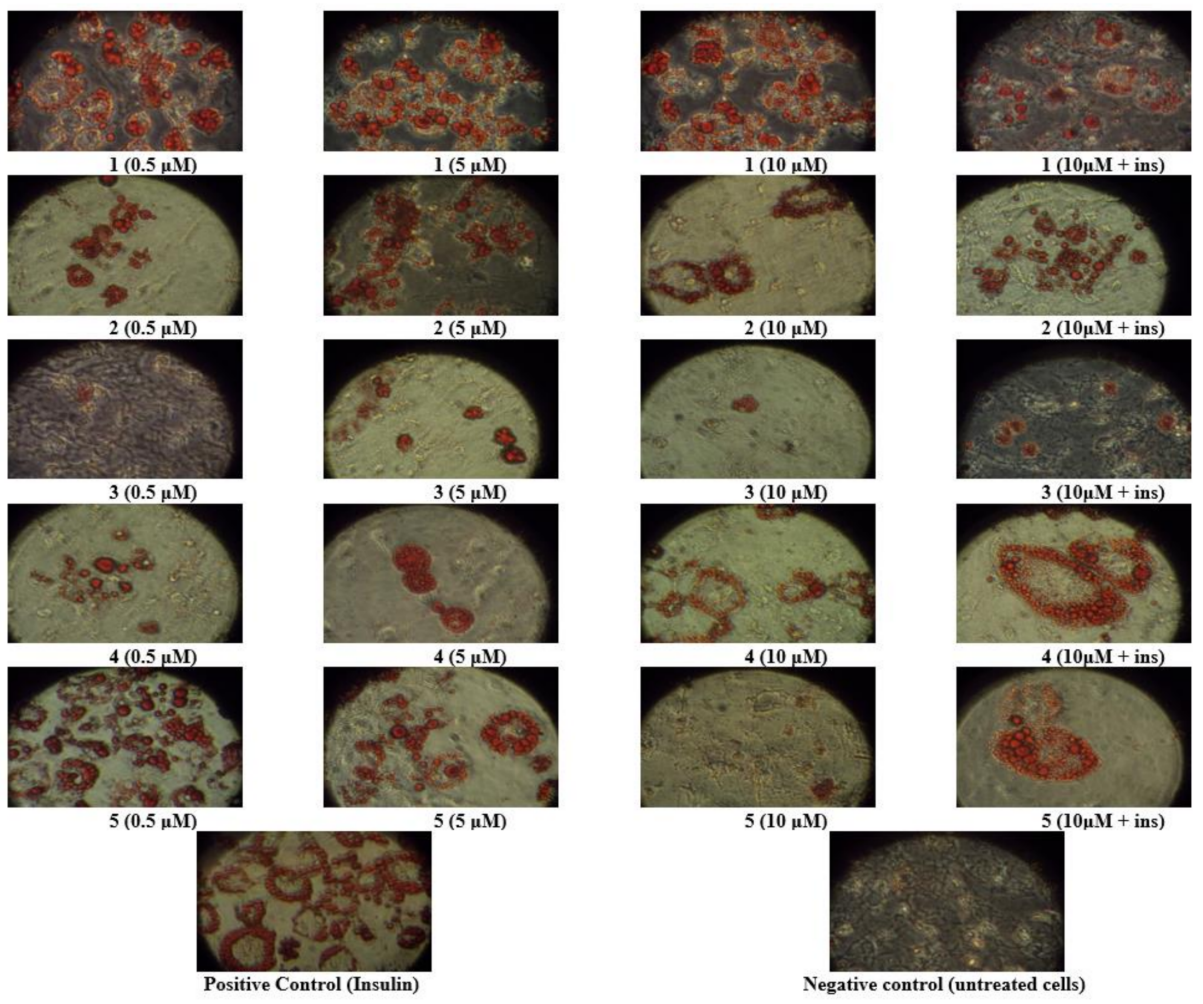
Representative micrographs of 3T3-L1 pre-adipocyte differentiation as assessed by oil red O staining.

To investigate the adipogenic potential and thus insulin mimetic and/or enhancing activity, the title compounds were used to induce cell differentiation of 3T3-L1 fibroblasts into mature adipocytes, following a standard differentiation protocol. Compounds were used to induce differentiation by the complete replacement of insulin or in combination with insulin to further investigate potential synergistic/inhibiting effects. Compounds **1**–**5** induced differentiation of pre-adipocytes into mature adipocytes in most cases tested (0.5–10 μΜ), in a concentration-dependent manner, as that was assessed through oil red O staining (Figure 10). Untreated cells served as negative control (Figure 10). The so obtained results were comparable to the differentiation effect induced by insulin (10 ng/mL), which serves as a positive control of the assay (Figure 10). The results suggest a ligand-dependent synergistic and/or enhancing relationship between zinc compounds and insulin. More specifically, complex **1** and **2** project high lipogenesis in almost all cases tested, whereas **3** projects a limited number of lipid droplets. 

#### 2.7.3. Biomarker Gene Expression

**Figure 11:**
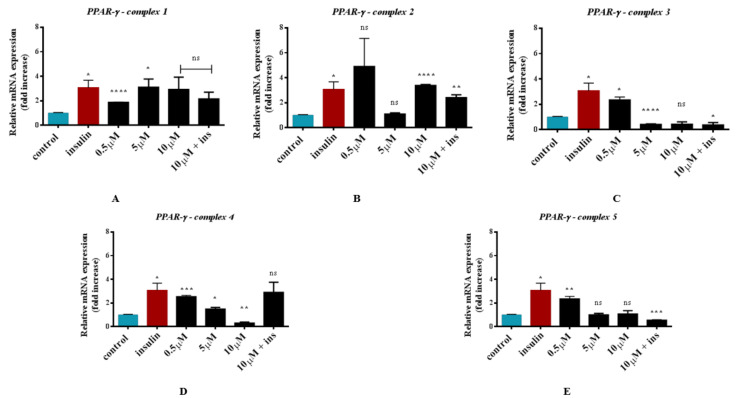
Relative concentration of mRNA expression for PPAR-γ for complex **1** (**A**), complex **2** (**B**), complex **3** (**C**), complex **4** (**D**), and complex **5** (**E**), in mature adipocytes compared to pre-adipocytes *p 0.05 (significant), ** *p* 0.01 (highly significant), *** *p* 0.001 (extremely significant), and **** *p* ≤ 0.0001 (extremely significant) or non-significant (*p* 0.05) vs. control.

The adipogenic potential of the newly synthesized materials was further evaluated through the expression of closely related genes that serve as biomarkers. In so doing, RT-PCR was run to examine the relative mRNA expression of PPAR-γ, GLUT 1, and GLUT 4 at several concentrations, on the 8th day of the differentiation process. Initially, PPAR-γ was examined, since it serves as a key regulator of the differentiation process. The results show that PPAR-γ is expressed in most cases and the effect was comparable to that of insulin, whereas in some cases, the signal was even higher than that of the positive control (Figure 11). The relative mRNA expression of PPAR-γ in the case of insulin, under the employed experimental conditions, was 3.1. Compound **1** exhibits a 1.9-fold increase at 0.5 μΜ concentration, a 3.1-fold increase at 5 μΜ, a 2.9-fold increase at 10 μΜ, and a 2.1-fold increase when cells are differentiated in combination with insulin and compound **1** (10 μΜ) (Figure 11A). In the case of **2**, PPAR-γ was expressed at 0.5 μΜ with a 4.9-fold increase and at 10 μΜ with a 3.4-fold increase (Figure 11B). No expression was observed in the case of 5 μΜ, whereas when cells were treated in combination with insulin, a 2.4-fold increase was observed. The relative mRNA expression of PPAR-γ in the case of **3** exhibits a 2.3-fold increase at 0.5 μΜ. No expression was observed at higher concentrations or in combination with insulin (Figure 11C). In the case of **4**, PPAR-γ projects a 2.5-fold increase at 0.5 μΜ, a 1.5-fold increase at 5 μΜ, a 0.3-fold change at 10 μΜ, and a 2.9-fold increase when cells are treated in combination with insulin (Figure 11D). By the same token, compound **5** projects a 2.3-fold increase at 0.5 μΜ. For the rest of the cases, no expression was observed (Figure 11E). 

**Figure 12:**
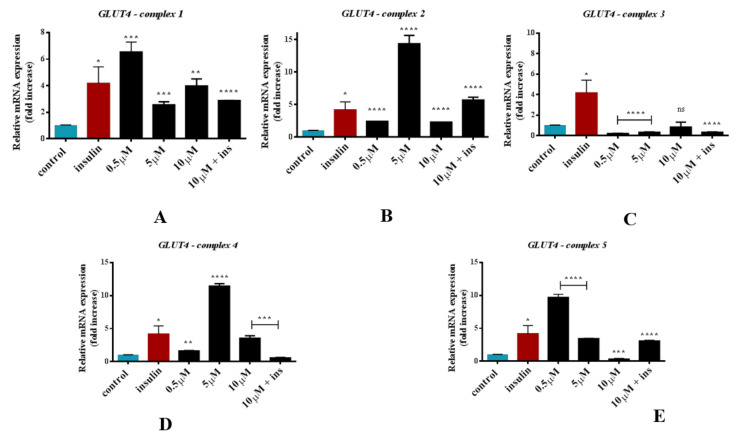
Relative concentration of mRNA expression for GLUT 4 for complex **1** (**A**), complex **2** (**B**), complex **3** (**C**), complex **4** (**D**), and complex **5** (**E**), in mature adipocytes compared to pre-adipocytes * *p* 0.05 (significant), ** *p* 0.01 (highly significant), *** *p* 0.001 (extremely significant), and **** *p* ≤ 0.0001 (extremely significant) or non-significant (*p* 0.05) vs. control.

Following PPAR-γ, GLUT 4, which is expressed only in mature adipocytes and serves as a biomarker of successful adipogenesis, was also examined. The results show a 4.6-fold increase when cells are induced to differentiate in the presence of insulin (standard differentiation protocol, positive control). In case of **1**, a 6.5-fold increase was observed at 0.5 μΜ, a 2.6-fold increase was observed at 5 μΜ, and a 4.0-fold increase was observed at 10 μΜ. A 2.9-fold increase in expression was observed when cells are treated in combination with insulin (Figure 12A). In the case of **2**, GLUT 4 exhibits a 2.4-fold increase at 0.5 μΜ, a 14.4-fold increase at 5 μΜ, and a 2.3-fold increase at 10 μΜ. When cells are differentiated in the presence of both **2** and insulin, a 5.7-fold increase is observed (Figure 12B). Complex **3** fails to induce the expression of GLUT 4 in all cases tested under the employed experimental conditions (Figure 12C). A 1.6-fold increase was observed at 0.5 μΜ, a 11.4-fold increase was observed at 5 μΜ, and a 3.5-fold increase was observed at 10 μΜ in the case of **4**. No expression was achieved when cells were differentiated in the presence of both **4** and insulin (Figure 12D). By the same token, in the case of **5**, GLUT 4 expression shows a 9.7-fold increase at 0.5 μΜ, a 3.4-fold increase at 5 μΜ, and 3.1-fold increase in combination with insulin. No expression was achieved when cells were differentiated in the presence of **5** at 10 μΜ (Figure 12E). Moreover, the relative mRNA expression of GLUT1 was also examined. The expression in the case of GLUT 1 projects no significant change between pre-mature and mature adipocytes (data not shown), as that was expected, since GLUT 1 is idiostatically expressed in all cell types.

### 2.8. Cell Signaling Studies

**Figure 13:**
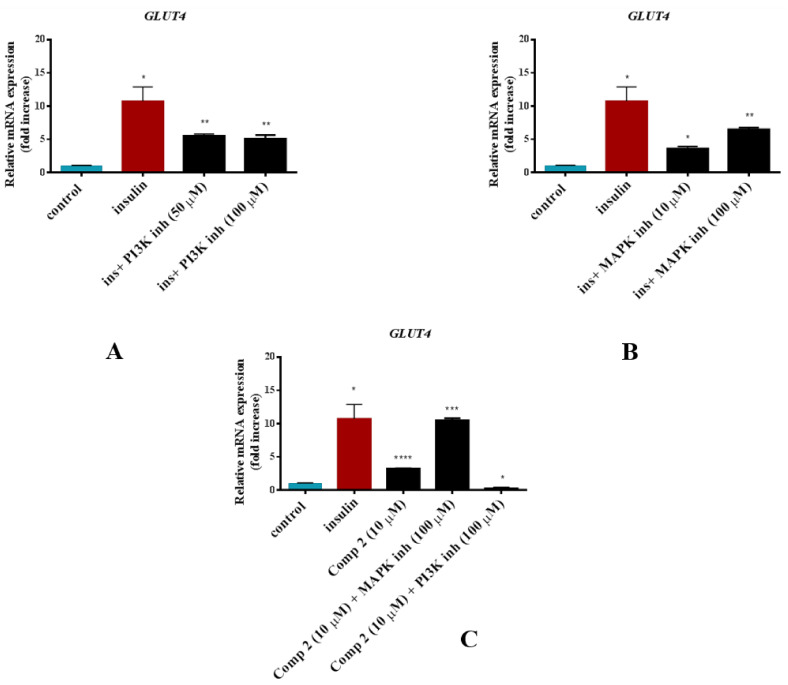
Relative concentration of mRNA expression for GLUT 4 in the presence of (**A**) PI3K inhibitor, (**B**) MAPK inhibitor, and (**C**) complex 2, in mature adipocytes compared to pre-adipocytes * *p* 0.05 (significant), ** *p* 0.01 (highly significant), *** *p* 0.001 (extremely significant) and **** *p* ≤ 0.0001 (extremely significant) or non-significant (*p* 0.05) vs. control.

For the effect of PI3-kinase and MAPK inhibitors on 3T3-L1 adipocyte differentiation induction, post-confluent cells were pretreated with each individual inhibitor at the indicated concentration for 2 h and then induced to differentiate, following the standard differentiation induction protocol. The inhibitors were added prior to the induction process during the first 2 h and were then removed. Cells were stained with oil red O on day 8, and whole mRNA was extracted in order to assess the relative mRNA expression of GLUT 4. Since GLUT 4 is expressed only in mature adipocytes, it was considered as an indicator of successful adipogenesis at the genetic level. Pre-adipocytes were induced to differentiate in the presence of the inhibitors following treatment with compound **2**. Adipogenesis in the presence of insulin was again considered as positive control, whereas cell differentiation in the presence of each inhibitor and insulin was also included in the study as additional controls (Figure 13A,B). As shown in Figure 13, when cells are treated with the PI3K inhibitor prior to induction of adipogenesis, the expression of GLUT 4 is significantly reduced (≈5.5-fold change) compared to that of insulin alone (10.7-fold change) for both concentrations of the inhibitor. In the case of MAPK inhibition, GLUT 4 expression is significant when cells are treated with the MAPK inhibitor in a concentration-dependent manner (3.6 and 6.5-fold increase for 10 and 100 μΜ, respectively), although a reduction in expression is observed compared to insulin. When cells are treated with **2** in the presence of the PI3K inhibitor, complete suppression is observed (0.2-fold increase), indicating an inhibition of adipogenesis. Interestingly, in the case of MAPK inhibition, a significant expression of GLUT 4 is observed (10.5-fold increase) that is even higher than the complex itself (3.2-fold increase) (Figure 13C). Taken together, the inhibition of PI3K significantly reduces adipogenesis, whereas inhibition of MAPK increases adipogenesis (also reported in the literature) [25,26]. The results indicate that in the case of **2**, cell differentiation is mediated by the PI3K signaling pathway, and there is a clear synergistic effect with the MAPK inhibitor (via inhibition of the MAPK signaling pathway).

## 3. Discussion

### 3.1. From Hydroxycarboxylic Acids to Zwitterions in Zinc Chemistry

The quest for appropriately configured forms of zinc (zincoforms), which are capable of inducing cellular differentiation linked to insulin resistance in metabolic syndromes, rides heavily on the organic substrate bound to Zn(II) and the associated complex species speciation. Poised to peruse such crystallographically characterized well-defined forms of Zn(II) as potential insulin mimetics in diabetes mellitus II, a series of organic substrates were chosen to pursue the synthesis of complex zincoforms at the binary and ternary level. To that end, a family of (hydroxy)carboxylic acid substrates was selected with distinct structural characteristics in their molecular assembly. Specifically, trigonelline was chosen to represent a category of organic acids possessing zwitterionic characteristics, with the second category involving HeidaH_2_, representing the broader family of hydroxycarboxylic acids. In an effort to expand that class of substrates, a ternary partner, namely the N,N’-chelator 1,10-phenanthroline, was employed to serve as a reference chelator molecule capable of extending the chemically derived Zn(II) complex species to ternary hybrid metal–organic forms. In all of the aforementioned cases, the structural attributes of the employed organic substrates (a) were well-defined and promoted Zn(II) ion binding in various modes of coordination, and (b) contributed significantly to the solubility of the arising synthetic complex forms of Zn(II), thereby providing ample support for bioavailable species that would intervene in the ensuing in vitro biological studies and specifically insulin mimesis. The nature of the employed ligands and their incorporation in the synthetically studied systems of Zn(II) was key to pursuing isolable crystalline materials with well-defined physicochemical characteristics. It appears that the choice of ligands made, and the family of Zn(II) complex species designed and subsequently synthesized, worked successfully (vide infra).

### 3.2. Structural Specificity of Zinc in the Biology of Adipogenesis

Having selected the organic substrates according to the logic outlined above, the pursued synthetic investigation at the binary level first involved the zwitterionic ligand trigonelline. The molecule, containing a well-defined separation of positive and negative charges, exemplifies vividly the methylation process on the nitrogen atom of the aromatic ring applied onto vitamin B_3_ (niacin), concurrently being a metabolic product excreted in the urine of mammals. The coordination mode of the zwitterion to the Zn(II) center in compound **1** revealed a tetrahedral arrangement of two organic trigonelline ligands bound to the metal center in a unidentate fashion, with the remaining two sites occupied by chloride ligands present in the originally employed hydrochloride salt of the starting ligand reagent itself. In such a tetrahedral complex assembly, both of the trigonelline ligands retain their zwitterionic nature, thereby generating an outer shell of positive charge while concurrently maintaining an overall neutral charge in the entire complex. The thus-generated hybrid molecule exhibits pronounced solubility in water, thereby rendering itself a good candidate for further biological studies on adipogenesis. 

The second category of ligands involves HeidaH_2_ in both binary and ternary systems with Zn(II) and phen. Specifically, the binary Zn(II)-HeidaH_2_ system initially led to the isolation of crystalline molecule **2**. It is a polymeric compound, with the monomeric unit containing a mononuclear octahedral Zn(II) assembly comprised of a tridentate coordinating [Heida]^2−^ ligand and a water molecule, with an additional water solvent molecule present in the generated lattice. The ligand itself uses all three terminal anchors to bind Zn(III), thus itself serving as an anchor to an abutting Zn(II) assembly, extending through one of its carboxylate oxygens the polymeric chain in the lattice of **2**, while concurrently reflecting a potential site for further chemistry with ternary substrates (e.g., of biological interest) through the labile water ligand bound to Zn(II).

In a further attempt to enrich the coordination sphere of Zn(II) and generate a hybrid ternary species, phen was introduced in the synthetically investigated system, thus giving rise to isolable crystalline mononuclear assemblies in the generated lattice of **3**. It is worth emphasizing in this structure the fact that (a) π–π interactions dominate in the architectural arrangement of the mononuclear species, and (b) solvent water molecules give rise to hydrogen bonds that collectively assemble ‘tape’ structures within the lattice, comprised of four and six membered rings of planar and chair conformation, respectively. Thus, upon going from the binary to the ternary system, the following observations can be made: (a) the basic [Zn(C_6_H_9_NO_5_)]^o^ unit in **2** is maintained, and (b) the bound water molecule and the additional (sixth) coordination site in **2**, occupied by a terminal carboxylato oxygen from an adjacently located mononuclear assembly, are occupied by the N,N’-aromatic chelators phen. In doing so, the 3D character of the lattice of **2** is maintained (hydrogen bonds), yet it gets enriched with π–π interactions.

In the case of compound **4**, the idea of the [Heida]^2−^ ligand, being present in the coordination sphere of Zn(II), is abandoned, and the derived species contains only one phen molecule. To this end, the coordination geometry around zinc becomes tetrahedral. The remainder of the coordination sites are occupied by chloride ions, which are present in the original reaction mixture. The tetrahedral arrangement of ligands around Zn(II) does not prevent the structural arrangement of species to assume a lattice architecture of 3D, with the presence of C-H^…^Cl interactions contributing to that specific dimensionality.

Finally, when a second phen ligand enters the coordination sphere of Zn(II), the arising species in compound **5** contains one chloride ion bound to the metal ion, with the coordination number of the metal ionic sphere rising to 5, thus rendering the geometry trigonal bipyramidal. The dimensionality of the arising lattice is 3D, with the same factor contributing to the achievement of that as in the case of compound **4**.

The physicochemical properties of all derived species were investigated in the solid state and in solution, in an effort to generate a profile that would help in the ensuing biological studies. To that end, elemental analysis, FT-IR, NMR, TGA, and X-ray crystallography were essential in defining molecular properties, including the three-dimensional structure of all species. Attention is drawn to the family of compounds **2**–**5**, where progressive introduction of the N,N’-aromatic chelator phen induces profound effects in the solid state and solution electronic properties of the family structural members. Compound **2** is the binary species of Zn(II) with [Heida]^2−^ in a 1:1 stoichiometry, with structural features (bound water molecule) predicating further incorporation of the aromatic chelator phen in the coordination sphere of Zn(II). In the emerging ternary Zn(II)-Heida-phen species (compound **3**), the coordination sphere of Zn(II) remains intact (octahedral). In a yet additional step, which is consistent with the broad coverage of ligand-specific structural speciation of Zn(II) in the presence of the two substrates, an abstention of [Heida]^2−^ incorporation in the metal ion coordination sphere changes the coordination geometry around Zn(II), reducing it to tetrahedral (compound **4**). Due attention is drawn to the fact that the specific compound had been previously crystallized yet the herein reported crystallographic signature is different from the one reported in the past [21], thereby prompting report of the structure and comprehensive elaboration of the physicochemical features of the species derived in this study. Further incorporation of a second phen in the coordination sphere around Zn(II) afforded an additional change in the geometry around the metal ion, ultimately reflecting a trigonal bipyramidal arrangement of two phen ligands and a chloride ion. This structure, too, had been previously reported in the literature [21], yet the crystallographic signature was also different from the one presented in this study, thereby prompting an extensive and comprehensive description of the species (compound **5**). Finally, ESI-MS spectrometry on all materials was useful in providing the solution signature, so it is necessary for all species to enter the ensuing in vitro biological studies on adipogenesis. The structural variations observed in **1**–**5** were apparently linked to the well-defined signatures obtained through mass spectrometry. The so-defined profile of the species in the solid state and solution presents a reliable set of assemblies to be accounted for in the wake of the biological experiments to ensue. Evidently, the mononuclear identity of all complex unit assemblies investigated is retained in solution, which is also supported by NMR, with the arising species being in line with the ones encountered and explicitly described in the solid state (vide supra). 

Therefore, collectively, the structural speciation of the family of species examined here and involving the two ligands, i.e., hydroxycarboxylic [Heida]^2−^ and phen, affirms the need for well-defined species, when such species are slated for employment in vitro biological studies encompassing the Zn(II) ion.

### 3.3. Luminescence

A significant effort was also made to investigate potential luminescent properties of the derived materials, with all compounds exhibiting emission at discrete energies in the UV-Visible range. Compound **1** exhibits its own luminescent signature, owing to the presence of the zwitterionic trigonelline ligand in the coordination sphere of Zn(II) in a tetrahedral environment. Quite interesting, in that respect, was the case of compounds **2** and **3**, with the former species being the binary and the latter species being the ternary assemblies containing both [Heida]^2−^ and phen. It appears that the emission wavelength of the ternary species is shifted to longer wavelengths (lower energies) compared to the binary species, albeit at different wavelengths of excitation. To that end, a recognition signature arises in this case, with the structural identity of the species linked to distinct electronic properties associated with luminescence. A similar shift toward progressively lower energies (379 and 398 nm in **4**) is observed, when the coordination sphere of Zn(II) continues to be modified upon departure of the [Heida]^2−^ligand from the complex assembly, leaving the Zn(II) ion only in the presence of phen. Further introduction of an additional phen chelator in the coordination sphere of Zn(II) results in an even lower energy emission of light in **5** (449 nm). However, at different energies of excitation, the observed progressive energy reduction of the emission wavelength of light, in the series of compounds **2**–**5**, is linked to the gradual replacement of the [Heida]^2−^ ligand by phen chelators, despite the fact that toward the end of the series (compounds **4** and **5**), the coordination geometry around Zn(II) changes from octahedral (**2**,**3**) to tetrahedral (**4**) and trigonal bipyramidal (**5**). The importance of the observations made signifies the essence of further studies toward delineation of the electronic properties of rationally designed metal–organic species, sequentially traversing from one end of the ligand participating in binary assemblies to another ligand participating in ternary assemblies so as to peruse the potential for the development of new luminescent sensors. The quest for new knowledge in the specific family of species continues in our lab.

### 3.4. The Insulin Mimetic Potential of Hybrid Zinc Metal–Organics

Prior to assessing the insulin mimetic activity of the newly synthesized materials, it was deemed of great priority to evaluate their effect on cell survival. To this end, cell viability was investigated in the presence of **1**–**5** at several concentrations (0.5–100 μΜ) over both short (24 h) and long (48 h) incubation times. Moreover, cell survival was also assessed with regard to cell type (pre-adipocytes vs. mature adipocytes). Compounds **1**, **2**, and **4** appear to be completely atoxic at all concentrations tested for 24 h in the case of pre-adipocytes. Complexes **3** and **5** project a slight reduction in cell survival. For longer incubation times, cell survival is not affected in the case of **1** and **2**, whereas a slight, concentration-dependent reduction is observed for **3**, **4**, and **5**. These results further support the structure-specific impact of the substrates to which Zn(II) is bound. Moreover, taking into account that almost all compounds (except **3** at higher concentrations) appear to be completely atoxic, it is worth noting that the examined Zn(II) species project tissue-related specificity as well. In general, the results suggest that all compounds can be further employed in biomimetic activity assays. In the current experimental design, Zn(II) complex concentrations up to 10 μΜ were considered atoxic, thereby justifying further employment in cell differentiation assays. It is also worth mentioning the fact that complexation of Zn(II) with specific substrates enhances solubility and bioavailability, given that Zn(II)-nitrate (it serves as the starting material for the synthesis of **1**–**5**) projects significantly low solubility at pH 7.4, which is the pH for all investigated biological processes. 

To investigate the potential effects of the title compounds on the endogenous cell migration of 3T3-L1 pre-adipocytes, an in vitro scratch assay was performed. The physiological migrating activity of cells, grown in the presence of only DMEM, was considered as control. Cells were treated with 10 μΜ of **1**–**5** for 24 h. As shown in Figure S6, cells exhibited normal migration in the case of **1**, **2**, and **5**, whereas a slight inhibition is observed in **3**. Complete inhibition of cell motility is observed in **4** compared to control (untreated cells). No aberration from the normal state was observed for both undifferentiated and differentiated cells (Figure S7). The specific qualitative assay presents itself as a useful method for monitoring the anti-migratory activity of drug candidates. In the present study, the results show a non-significant lower migration ability compared to the control in most cases (untreated cells—incubated only in the presence of medium). 

To assess the adipogenic and hence insulin mimetic activity of the title compounds, cells were treated with several concentrations (0.5–10 μΜ) of **1**–**5**, according to a standard cell differentiation protocol. The success of the process of adipogenesis was based on oil red O staining along with the relative mRNA expression of tissue-specific markers (in this case, PPAR-γ and GLUT 4). Cell differentiation in the presence of insulin was considered as the positive control of the assay, whereas a negative control (completely untreated cells) was also included. All compounds were used by completely replacing insulin during the adipogenesis process or in combination with insulin. To that end, it appears that compound **1** successfully induces cell differentiation (compared to control) at all concentrations tested, when it replaces insulin. When **1** is used in combination with insulin, the result is comparable to that of insulin alone, thus indicating a non-inhibiting/competitive effect toward adipogenesis. In the case of **4**, cell differentiation is achieved for all concentrations tested. Only in the case of 5 μΜ was the effect higher than that of insulin. When **4** is used in combination with insulin, an inhibiting effect toward insulin activity is observed. By the same token, **5** induces adipogenesis, and the observed effect is comparable to that of insulin. Interestingly, the effect grows weaker with increasing concentrations. When **5** is used in combination with insulin, the result is comparable to that of insulin alone, thereby indicating a non-inhibiting/competitive effect toward adipogenesis. Similar results are also obtained in the case of **2**. What is worth noting is that when **2** is used in combination with insulin, the result is higher than in the case of insulin alone or complex **2** at the same concentration (10 μΜ) alone, thereby indicating a potential synergistic effect in cell maturation. Compound **3** failed to induce adipogenesis at all concentrations tested or in combination with insulin. This result can be partially explained due to its slight toxicity effect compared to compounds **1**, **2**, **4**, and **5**. 

A second round of experiments was also performed to investigate the interplay of the newly synthesized forms of zinc with key components of adipogenesis signaling. For that reason, complex **2** was selected for further investigation given its biological behavior (completely atoxic and enhanced adipogenic capacity). It is well known that in post-confluent 3T3-L1 pre-adipocytes, the signal cascade is launched, following activation of the IGF-1 receptor, although the pathways involved are not fully explored [25]. The very first step, following induction of differentiation, involves cell cycle re-entry and initiation of the adipogenesis process. IGF-1 receptor signaling can activate two kinase systems, namely the MAP kinase and the phosphatidylinositol 3-kinase-protein kinase B/Akt (PI3K), in 3T3-L1 pre-adipocytes. It is well-established that the PI3K cascade is involved in a plethora of vital cellular events (proliferation, apoptosis, etc.) [25]. It has been shown that (a) the inhibition of PI3K can lead to the blockage of adipogenesis in 3T3-L1 cells [27] and (b) the ectopic expression of the constitutively active form of PKB/Akt may lead to spontaneous adipogenesis [28] However, the initiation of IGF-receptor activity is considered as an upstream event, and insulin can still activate the IGF-1 receptor signaling cascade in the presence of PI3K inhibitors [25] On the other hand, it was shown that the inhibition of p38MAPK increases adipogenesis. Moreover, it has been suggested that p38MAPK activity is significantly higher in pre-adipocytes than in mature adipocytes, thus indicating that p38MAPK activity decreases during adipocyte differentiation, and in vitro inhibition of p38MAPK may increase expression of closely related adipocyte biomarkers [26,29] In the present study, two inhibitors (PI3K and MAPK inhibitors) were used in an effort to investigate the mechanism of action of Zn(II) complexes. The results further support the fact that MAPK inhibition leads to a more effective adipogenesis and that the effect is concentration-dependent. The effect of PI3K inhibition was comparable to that of insulin and negative control, yet it failed to completely suppress the expression of GLUT 4 (biomarker of mature adipocytes) under the employed experimental conditions. The results support the fact that **2** induces adipogenesis via the PI3K signaling pathway, and there is a clear synergistic effect with the MAPK inhibitor (via inhibition of the MAPK signaling pathway). This observation is in line with the literature and the fact that the inhibition of MAPK results in decreased proliferation, which is a key step to initiating adipogenesis. However, it is worth mentioning that the molecular targets of zinc signals are still not completely delineated. It has been shown that increased intracellular free zinc can activate MAPK activity and that zinc exerts an insulin-like effect toward glucose uptake mediated by PI3K and Akt in 3T3-L1 fibroblasts and adipocytes [30,31]. Taken together, the case of the herein developed Zn(II) species in adipogenesis formulates a representative model, with distinct molecular structural and biochemical characteristics, which might serve in meaningful comparisons when rationalizing the impact of structural speciation on insulin biomimesis. However, given the complexity of the interplay of numerous components (transcription factors, genes, signaling pathways, epigenetic regulators, and microRNAs) during adipogenesis and the impact of zinc alone in multiple determinants and pathways, more studies are required in order to fully delineate the exact role of zinc in insulin mimesis. 

## 4. Materials and Methods

All experiments were carried out in air. Nanopure quality water was used for all reactions run. Zn(CH_3_COO)_2_•2H_2_O, Zn(ΝO_3_)_2_•6H_2_O, trigonelline hydrochloride (C_7_H_8_NO_2_Cl), N-(2-hydroxyethyl)iminodiacetic acid (C_6_H_11_NO_5_, HeidaH_2_), and 1,10-phenanthroline (C_12_H_8_N_2_, phen) were purchased from Aldrich. Ammonia and sodium hydroxide were supplied by Fluka.

### 4.1. Physical Measurements

FT-infrared spectra were recorded on a Thermo Nicolet IR 200 FT-IR spectrometer. A Thermo Finnigan Flash EA 1112 CHNS elemental analyzer was used for the simultaneous determination of carbon, hydrogen, and nitrogen (%). The analyzer is based on the dynamic flash combustion of the sample (at 1800 °C) followed by reduction, trapping, complete GC separation, and detection of the products. The instrument is fully automated and controlled by PC via the Eager 300 dedicated software. It is capable of handling solid, liquid, or gaseous substances.

Electrospray ionization mass spectrometry (ESI-MS) infusion experiments were carried out on a ThermoFisher Scientific (Bremen, Germany) model LTQ Orbitrap Discovery MS. The trigonelline ligand, (L_1_ = {C_7_H_7_NO_2_}, trigonelline), HeidaH_2_ ligand, (L_2_H_2_ = HeidaH_2_), and compounds [Zn(L_1_)_2_Cl_2_] (**1**), [Zn(L_2_)(H_2_O)]•H_2_O (**2**), [Zn(L_2_)(phen)]•4H_2_O (**3**), [ZnCl_2_(phen)] (**4**), and [Zn(phen)_2_Cl](NO_3_)•H_2_O (**5**) were dissolved in water and introduced into the ESI source of the MS through an integrated syringe pump at a flow rate of 5 μL/min. The infusion experiments were run using a standard ESI source, operating in a positive ionization mode. Source operating conditions included a 3.7 kV spray voltage and a 320 °C heated capillary temperature. 

A TA Instruments thermal analyzer, model Q600, system was used to run thermogravimetric analysis (TGA) experiments. The employed heating rate was 5 °C/min. The instrument mass precision was 0.1 μg. About 18.5 mg of sample was placed in an open alumina sample pan for each experiment. High-purity helium and air (80/20 in N_2_/O_2_) were used at a constant flow rate of 100 mL/min, depending on the conditions required for running the experiment. During the experiment, the sample weight loss and rate of sample weight loss were recorded continuously under dynamic conditions, as a function of time or temperature, in the range 30–1000 °C. Prior to activating the heating routine program, the entire system was purged with the appropriate gas for 10 min, at a rate of 400 mL/min, to ensure that the desired environment had been established.

Luminescence measurements and more specifically, solid-state emission and excitation spectra (200-900 nm) were recorded on a Hitachi F-7000 fluorescence spectrophotometer from Hitachi High-Technologies Corporation. The employed split widths (em, ex) were 5.0 nm, and the scan speed was 1200 nm·min^−1^. All measurements were carried out at room temperature. The entire system was supported by the appropriate computer software, FL Solutions 2.1, running on Windows XP.

Solution ^1^H- and ^13^C-NMR experiments for **1**–**5** were carried out on a Varian 600 MHz spectrometer. The sample concentration was ≈5 mM. Freshly prepared compounds were dissolved in D_2_O. Carbon spectra were acquired with 5000 transients, a spectral width of 20,000 Hz, and a relaxation delay of 2s. Proton spectra were acquired with 2046 transients and a spectral width of 9000 Hz. Experimental data were processed using VNMR routines. Spectra were zero-filled and subjected to exponential apodization prior to FT. Chemical shifts (δ) are reported in ppm, while spectra were referenced by the standard experimental setup.

### 4.2. Syntheses

#### 4.2.1. Synthesis of Zn(C_7_H_7_NO_2_)_2_Cl_2_ (**1**)

Method A: A quantity of 0.15 g (0.63 mmoL) Zn(CH_3_COO)_2_•2H_2_O was placed in a flask and dissolved in 3 mL of H_2_O. Subsequently, trigonelline hydrochloride 0.24 g (1.38 mmoL) was added slowly and under continuous stirring. At first, trigonelline hydrochloride was not soluble. The solution was heated to 70 °C for 1 h and then cooled to room temperature. The pH of the reaction was 3.5. Dissolution of the reaction mixture was achieved upon the addition of HCl to adjust the pH to a final value of ≈2. The resulting reaction solution was stirred overnight. Then, the derived mixture was left for slow evaporation. A few days later, crystals appeared at the bottom of the vial. The crystals were isolated by filtration and dried in vacuo. The yield was 0.20 g (70%). Anal. Calcd. for **1**, Zn(C_7_H_7_NO_2_)_2_Cl_2_, C_14_H_14_Cl_2_N_2_O_4_Zn, M.W. 410.54: C 40.90, H 3.41, Ν 6.15; Found: C 40.88, H 3.42, Ν 6.13. FT-IR (KBr, cm^−1^) for **1**: ν_as_(COO^–^): 1660–1625 cm^−1^, ν_s_(COO^–^): 1374–1286 cm^−1^.

Method B: The same quantities of starting material solids were dissolved in 13 mL of H_2_O. The final pH value was ≈3.5. The arising reaction mixture was allowed to stir at room temperature for ½ h. Then, it was placed in a Teflon-lined stainless-steel reactor (23 mL) and heated to 180 °C for 5 days. Upon cooling of the reactor to room temperature, crystals appeared at the bottom of the vessel. They were isolated by filtration, water-washed, and dried in the air. The yield was 0.15 g (40%). Anal. Calcd. for **1**, Zn(C_7_H_7_NO_2_)_2_Cl_2_, C_14_H_14_Cl_2_N_2_O_4_Zn, M.W. 410.54: C 40.90, H 3.41, Ν 6.15; Found: C 40.87, H 3.40, Ν 6.14.

#### 4.2.2. Synthesis of [Zn(C_6_H_9_NO_5_)(H_2_O)]_n_ • nH_2_O (**2**)

Method A: A quantity of 0.29 g (1.0 mmoL) Zn(ΝO_3_)_2_•6H_2_O was placed in a flask and dissolved in 5 mL of H_2_O. Subsequently, N-(2-hydroxyethyl)iminodiacetic acid 0.17 g (1.0 mmoL) was added slowly and under continuous stirring. The pH of the reaction was adjusted upon addition of ammonia solution to a final value of ≈7.0. The resulting solution was stirred overnight. On the following morning, addition of cold ethanol at 4 °C resulted after a month in the deposition of crystalline material. The crystals were isolated by filtration and dried in vacuo. The yield was 0.23 g (88%). Anal. Calcd. for **2**, [Zn(C_6_H_9_NO_5_)(H_2_O)] • H_2_O, C_6_H_13_NO_7_Zn, M.W. 276.54: C 26.05, H 4.71, Ν 4.99; Found: C 26.01, H 4.75, Ν 4.94. FT-IR (KBr, cm^−1^) for **2**: ν_as_(COO^–^): 1585 cm^−1^, ν_s_(COO^–^): 1401–1380 cm^−1^.

Method B**:** The same quantities of solids were dissolved in 13 mL of H_2_O. The final pH value was ≈3.5. The arising reaction mixture was allowed to stir at room temperature for ½ h. Then, it was placed in a Teflon-lined stainless-steel reactor (23 mL) and heated to 180 ^o^C for 5 days. Subsequently, the reactor was allowed to cool down to room temperature. The recovered solution was allowed to evaporate slowly, and a few days later, colorless crystals emerged. They were isolated by filtration, water-washed, and dried in the air. The yield was 0.20 g (77%). Calcd. for **2**, [Zn(C_6_H_9_NO_5_)(H_2_O)]•H_2_O, C_6_H_13_NO_7_Zn, M.W. 276.54: C 26.05, H 4.71, Ν 4.99; Found: C 25.96, H 4.68, Ν 4.95. FT-IR (KBr, cm^−1^) for **2**: ν_as_(COO^–^): 1585 cm^−1^, ν_s_(COO^–^): 1401–1380 cm^−1^.

#### 4.2.3. Synthesis of [Zn(C_6_H_9_NO_5_)(C_12_H_8_N_2_)]•4H_2_O (**3**)

Method A: A mixture of 0.11 g (0.50 mmoL) Zn(CH_3_COO)_2_•2H_2_O, 0.17 g (1.0 mmoL) 2-hydroxyethyliminodiacetic acid and 0.19 g (1.0 mmoL) phen were placed in a flask, in the sequence specified, and dissolved in 13 mL of H_2_O. The final pH value was ≈5. The reaction mixture was stirred for ½ h. Then, it was placed in a Teflon-lined stainless-steel reactor (23 mL) and heated to 180 °C for 5 days. Subsequently, the reactor was allowed to cool down to room temperature. The recovered solution was allowed to evaporate slowly, and a few days later, colorless crystals emerged. The crystals were isolated by filtration and dried in vacuo. The yield was 0.25 g (84%). Anal. Calcd. for **3**, [Zn(C_6_H_9_NO_5_)(C_12_H_8_N_2_)]•4H_2_O, C_18_H_25_N_3_O_9_Zn, M.W. 492.78: C 40.10, H 4.20, N 10.34; Found: C 40.13, H 4.17, Ν 10.31. FT-IR (KBr, cm^−1^) for **3**: ν_as_(COO^–^): 1609 cm^−1^, ν_s_(COO^–^): 1429–1380 cm^−1^.

Method B: The same quantities of starting material solids were dissolved in 13 mL of H_2_O. The final pH value of the reaction mixture was adjusted upon the addition of NaOH to the final pH value 9.0. The ensuing synthetic procedure was the same as mentioned above. The emerging crystalline material was isolated by filtration, water-washed, and dried in the air. The yield was 0.18 g (60%). Anal. Calcd. for **3**, [Zn(C_6_H_9_NO_5_)(C_12_H_8_N_2_)]•4H_2_O, C_18_H_25_N_3_O_9_Zn, M.W. 492.78: C 40.10, H 4.20, N 10.34; Found: C 40.06, H 4.18, Ν 10.36. FT-IR (KBr, cm^−1^) for **3**: ν_as_(COO^–^): 1609 cm^−1^, ν_s_(COO^–^): 1429–1380 cm^−1^.

#### 4.2.4. Synthesis of Zn(C_12_H_8_N_2_)Cl_2_ (**4**)

A mixture of 0.11 g (0.50 mmoL) Zn(CH_3_COO)_2_•2H_2_O, 0.18 g (1.0 mmoL) trigonelline hydrochloride, and 0.10 g (0.50 mmoL) phen were placed in a flask, in the sequence specified, and dissolved in 13 mL of H_2_O. The final pH value was ≈5. The reaction mixture was stirred for ½ h. Then, it was placed in a Teflon-lined stainless-steel reactor (23 mL) and heated to 180 °C for 5 days. Subsequently, the reactor was allowed to cool down to room temperature. The recovered solution was allowed to evaporate slowly, and a few days later, colorless crystals emerged. The crystals were isolated by filtration and dried in vacuo. The yield was 0.13 g (81%). Anal. Calcd. for **4**, Zn(C_12_H_8_N_2_)Cl_2_, C_12_H_8_Cl_2_N_2_Zn, M.W. 316.47: C 45.35, H 2.83, Ν 8.81; Found: C 45.32, H 2.79, Ν 8.82. FT-IR (KBr, cm^−1^) for **4**: C=N (ν_C=N_, 1638 cm^−1^) and C=C (ν_C=C_, 1585 cm^−1^), with the skeleton vibration peak of the ligand phen appearing at 1515 cm^−1^”.

#### 4.2.5. Synthesis of [Zn(C_12_H_8_N_2_)_2_Cl](NO_3_)•H_2_O (**5**)

A mixture of 0.20 g (0.67 mmoL) Zn(NO_3_)_2_•6H_2_O, 0.25 g (1.4 mmoL) trigonelline hydrochloride, and 0.13 g (0.70 mmoL) phen was placed in a flask, in the sequence specified, and dissolved in 13 mL of H_2_O. The final pH value was adjusted with a solution of sodium hydroxide at the final pH value ≈8. The reaction mixture was stirred for ½ h. Then, it was placed in a Teflon-lined stainless-steel reactor (23 mL) and heated to 180 °C for 5 days. Subsequently, the reactor was allowed to cool down to room temperature. The recovered solution was allowed to evaporate slowly and a few days later colorless crystals emerged. The crystals were isolated by filtration and dried in vacuo. The yield was 0.33 g (90%). Anal. Calcd. for **5**,^.^[Zn(C_12_H_8_N**_2_**)_2_Cl](NO_3_)•H_2_O, C_24_H_18_ClN_5_O_4_Zn, M.W. 541.25: C 53.00, H 3.59, Ν 12.89; Found: C 52.99, H 3.57, Ν 12.93. FT-IR (KBr, cm^−1^) for **5**: C=N (ν_C=N_, 1642 cm^−1^), with the skeleton vibration peak of the ligand phen appearing at 1515 cm^−1^”.

### 4.3. X-ray Crystal Structure Determination

X-ray quality crystals of compounds **1**–**5** were grown from aqueous solutions or mixtures of water–ethanol solutions. A single crystal, with dimensions 0.22 × 0.33 × 0.47 mm (**1**), 0.05 × 0.05 × 0.49 mm (**2**), 0.10 × 0.24 × 0.37 mm (**3**), 0.20 × 0.40 × 0.55 mm (**4**), and 0.35 × 0.43 × 0.65 mm (**5**) was taken from the mother liquor and immediately cooled to −113 °C. Diffraction measurements were made on a Rigaku R-AXIS SPIDER Image Plate diffractometer, using graphite monochromated Cu Kα radiation. Data collection (ω-scans) and processing (cell refinement, data reduction, and empirical absorption correction) were performed using the CrystalClear program package [32]. Important crystallographic data are listed in Table 1 and Table 2. The structures were solved by direct methods, using SHELXS v.2013/1, and refined by full-matrix least-squares techniques on F^2^ with SHELXL ver. 2014/6 [33,34]. Further experimental crystallographic details for **1**: 2*θ*_max_ = 130°; reflections collected/unique/used, 10097/2408 [R_int_ = 0.0476]/2408; 212 parameters refined; (*Δ*/*σ*)_max_ = 0.001; (*Δρ*)_max_/(*Δρ*)_min_ = 0.451/−0.453 e/Å^3^; *R*/*R*_w_ (for all data), 0.0464/0.0908. Further experimental crystallographic details for **2**: 2*θ*_max_ = 130°; reflections collected/unique/used, 12966/1694 [R_int_ = 0.1100]/1694; 188 parameters refined; (*Δ*/*σ*)_max_ = 0.001; (*Δρ*)_max_/(*Δρ*)_min_ = 1.115/−0.836 e/Å^3^; *R*/*R*_w_ (for all data), 0.0672/0.1200. Further experimental crystallographic details for **3**: 2*θ*_max_ = 130°; reflections collected/unique/used, 12597/3242 [R_int_ = 0.0416]/3242; 380 parameters refined; (*Δ*/*σ*)_max_ = 0.001; (*Δρ*)_max_/(*Δρ*)_min_ = 0.580/−0.331 e/Å^3^; *R*/*R*_w_ (for all data), 0.0342/0.0802. Further experimental crystallographic details for **4**: 2*θ*_max_ = 130°; reflections collected/unique/used, 12537/1938 [R_int_ = 0.0370]/1938; 186 parameters refined; (*Δ*/*σ*)_max_ = 0.000; (*Δρ*)_max_/(*Δρ*)_min_ = 0.340/−0.348 e/Å^3^; *R*/*R*_w_ (for all data), 0.0283/0.0710. Further experimental crystallographic details for **5**: 2*θ*_max_ = 130°; reflections collected/unique/used, 29199/3732 [R_int_ = 0.0477]/3732; 388 parameters refined; (*Δ*/*σ*)_max_ = 0.001; (*Δρ*)_max_/(*Δρ*)_min_ = 0.404/−0.530 e/Å^3^; *R*/*R*_w_ (for all data), 0.0370/0.0925. All hydrogen atoms were located by difference maps and were refined isotropically or were introduced at calculated positions as riding on bonded atoms. All non-hydrogen atoms were refined anisotropically. Plots of the structures were drawn using the Diamond 3 program package [35].

### 4.4. Cell Cultures and Biological Tests

#### 4.4.1. Cultivation of 3T3-L1 Cells

In the present study, the 3T3-L1 cell line (mouse pre-adipocytes) was employed to assess the insulin mimetic potential of the title complex zincoforms. The employed cell line is an established in vitro model for the study of chemically induced cell differentiation toward adipogenesis. Cells (both premature and mature adipocytes) were cultured in 75 cm^2^ cell culture flasks, under appropriately chosen conditions (5% CO_2_ at 37 ^o^C and standard humidity), in Dulbecco’s modified Eagle’s medium DMEM (Sigma, Steinheim, Germany). Culture media were supplemented with 10% Fetal Bovine Serum (FBS) (Biochrom, Berlin, Germany) and 1% penicillin–streptomycin (Biochrom, Berlin, Germany) prior to use. The specific cell line was tested and found free of mycoplasma contamination using the MycoAlert^®^ Kit (Promega). All experiments were run at least in triplicate, employing cells with a low passage number (P5–P9).

#### 4.4.2. Preparation of Zn(II) Compound Stock Solutions

Fresh stock solutions of the title compounds were prepared in DMEM (1% penicillin–streptomycin, 10% FBS). Prior to any experimental procedure, the solubility of the employed compound in water was assessed. All solutions were freshly prepared prior to all experiments (1 mM initial stock solution), followed by sterile filtration. Final working concentrations were applied directly onto the cell cultures and incubated for the desired time periods, according to the protocols followed.

#### 4.4.3. Cell Viability Growth Studies

Cell survival was assessed in the presence of the well-characterized Zn(II) compounds. Both premature and mature adipocytes were treated with various concentrations of the title compound(s) for 24 and 48 h. Cells were seeded in opaque 96 multi-well plates (2500 cells/well) and cell survival was examined using Cell Titer-Glo^®^. Briefly, the reagent (Promega kit Cell Titer-Glo^®^) was added to the cell culture (volumetric reagent/supernatant ratio 1:1) without removing the supernatant medium, as described elsewhere [36] The luminescence of each sample was measured on a GloMax^®^ 96 Microplate Luminometer (Promega Corporation, WI, USA). Results were expressed in relative percent change of luminescence units (RLU). Sodium deoxycholate, which is a known cytotoxic agent, served as positive control of the assay at a final concentration of 1 mg/mL, under light-protected conditions.

#### 4.4.4. Cell Biocompatibility—Morphology—Migration

Potential cytotoxic effects, in the presence of the materials tested, were also investigated with respect to cell morphology. To that end, both cell types were regularly examined with respect to shape, appearance, color, confluency, etc., to further confirm any aberration from healthy status. Images were captured, using an AxioCamHc camera, at several time points (prior to and post treatment). The motility of 3T3-L1 fibroblast-like cells was evaluated by the in vitro scratch assay. For that assay, cells were seeded in 35 mm cell culture dishes in DMEM. After achieving confluency (70–80%), a scratch was made in the monolayer over the total diameter of each culture dish, using a sterile pipette tip, and cells were allowed to grow in the culture medium in the presence of a final concentration of 10 μΜ of the emerging zinc complexes. Cells were visualized using an Axio Observer Z1 microscope, with a 10× phase contrast water immersion objective (Carl Zeiss, GmbH Lena, Germany). Images were captured, using an AxioCamHc camera, at 24 h after the scratch had been made [37].

#### 4.4.5. Induction of Adipogenesis

Pre-adipocyte differentiation into mature adipocytes was achieved by exposing pre-adipocytes to an appropriate adipogenic hormone cocktail (dexamethazone, 3-isobutyl-1-methylxanthine, and insulin), according to a standard differentiation protocol as described elsewhere [38] In brief, two days past 70% confluency (day 0), cells were treated with 0.5 mM 3-isobutyl-1-methylxanthine (Sigma, Germany), 1.0 μΜ dexamethazone (Sigma, Steinheim, Germany), and 10 ng/mL of insulin, as final concentrations in DMEM. Two days later (day 2), the cells were cultured in maintenance medium, treated only with insulin (main adipogenic factor) and the medium was serially changed every two days, whereas lipid formation was monitored on a daily basis. All experiments were performed on the 8th day of the differentiation process.

#### 4.4.6. Zn(II)-Induced Adipogenesis

3T3-L1 pre-adipocytes were differentiated into mature adipocytes following the standard differentiation protocol (vide supra), as described previously [38]. 3T3-L1 fibroblast-like cells were treated with either 10 ng/mL of insulin and/or Zn(II) complexes (0.5–50 μΜ). In all experiments run, the insulin group was considered as a positive control. A control group with untreated cells (without insulin or Zn(II)) was also included. Inhibitors of key components of insulin-signaling pathways were also used (MAPK (U0126, MEK 1/2 inhibitor) and PI3K (LY294002)). Inhibitors were added to cell cultures at a final working concentration of 10 or 100 μΜ in the case of PI3K, and 50 or 100 μΜ in the case of MAPK, on day 0 of the induction process, for a period of 2 h prior to addition of the induction media. Then, they were removed [39,40] On the 8th day of the differentiation process, cell differentiation was assessed with oil red O staining and real-time PCR for the relative expression of closely related biomarkers. All tests were carried out at least in triplicate. 

#### 4.4.7. Oil Red O Staining

To visualize the formation of fat droplets, oil red O staining was performed on the 8th day of the differentiation process. Briefly, cells were washed with PBS (1X, pH 7.4) and fixed with 4% formalin for 20 min. Then, cells were washed with sterile doubly deionized water (ddH_2_O) and treated with oil red O freshly prepared working solution for 15 min at room temperature. Subsequently, cells were washed with sterile ultrapure water and stained with hematoxylin for 1 min at room temperature (optional). Cells were directly visualized using an Axio Observer Z1 microscope, with a 10x and 40x phase contrast objective (Carl Zeiss, GmbH Jena, Germany). Images were captured on an AxioCamHc camera. All tests were carried out at least in triplicate.

##### 4.4.8. cDNA Synthesis and RT-PCR Assay

Total RNA was extracted from cells on the 8th day of the differentiation process, using Trizol (Life Science, Chemilab, Berkeley, CA, USA). Synthesis of cDNA was performed with the iScript cDNA synthesis kit (BioRad), according to manufacturer’s instructions. RT-PCR was run on Rotor Gene Q (Qiagen), using the QuantiTect SYBR Green PCR kit and appropriate reagents (Qiagen). Primer sequences are provided in Table 3. 

### 4.5. Statistical Analysis

The obtained data were presented as average and standard error mean (SEM) values of multiple sets of independent measurements. Mean survival rates and SEMs were calculated for each individual group. Absolute survival rates were calculated for each control group, and one-way analysis of variance (ANOVA) was performed for all pair comparisons, followed by post hoc analyses (Tukey) and Student’s t-test in the case of RT-PCR experiments, using GraphPad Prism v.6. Degrees of significance were assessed by three different rating values: * *p* 0.05 (significant), ** *p* 0.01 (highly significant), *** *p* 0.001 (extremely significant), and **** *p* ≤ 0.0001 (extremely significant) or non-significant (*p* 0.05).

## 5. Conclusions

Efforts to design and synthesize well-defined Zn(II) binary and ternary species capable of inducing cellular differentiation of pre-adipocytes under conditions reminiscent of insulin resistance in diabetes mellitus II led to the (a) employment of rationally formulated specific sets of organic substrates as potential Zn(II) binders and (b) isolation of well-defined crystalline materials at the binary and ternary level. The employment of (a) zwitterionic and (b) (hydroxy)carboxylic acid metal binders revealed discrete mononuclear Zn(II) assemblies, with composition and molecular stoichiometry in the arising complex assemblies being quintessential in further investigating their influence on cellular differentiation in in vitro biological studies. The solid-state and solution physicochemical properties of all materials derived, **1**–**5**, formulated a well-defined profile, thus enabling rationalization of the emerging results at the biological level. The in vitro biological studies rode on the concentration-dependent (a)toxicity profile of the title compounds and revealed their strength of adipogenicity on 3T3-L1 adipocyte cells. The observed adipogenic potential was further corroborated by gene-specific expression levels inquired into so as to delineate, assert, and validate observations linked to cell differentiation processes, ultimately leading to cell maturation. The nature of the organic substrates and the arising structural characteristics of the title compounds project the importance of ligand features in bestowing upon Zn(II) solubility, bioavailability, and interactive potential with genetic loci poised to influence cellular events vital to insulin mimesis in diabetes physiology. The collective physicochemical and biological results portray vividly the interwoven dependability of well-defined Zn(II) metal–organic species on the synthetic bioinorganic chemistry, which through distinct structural speciation profiles supports commensurably distinct insulin mimetic activity profiles. The advent of such collaborating data sets the basis for further in-depth perusal of Zn(II)-organic substrate metallopharmaceuticals as potential drugs in diabetes mellitus II.

## Figures and Tables

**Table 1:** Crystallographic data for compounds Zn(C_7_H_7_NO_2_)_2_Cl_2_ (**1**), [Zn(C_6_H_9_NO_5_)(H_2_O)]_n_•nH_2_O (**2**), [Zn(C_6_H_9_NO_5_)(C_12_H_8_N_2_)]•4H_2_O (**3**), Zn(C_12_H_8_N_2_)Cl_2_ (**4**), and [Zn(C_12_H_8_N_2_)_2_Cl](NO_3_)•H_2_O (**5**).

	1	2	3	4	5
Formula	C_14_H_14_Cl_2_N_2_O_4_Zn	C_6_H_13_NO_7_Zn	C_18_H_25_N_3_O_9_Zn	C_12_H_8_Cl_2_N_2_Zn	C_24_H_18_ClN_5_O_4_Zn
*Fw*	410.54	276.54	492.78	316.47	541.25
Space group	*P* $\bar{1}$	*P*2_1_/*c*	*P* $\bar{1}$	*P*2_1_/*n*	*P*2_1_/*n*
*a* (Å)	7.2582(1)	7.3509(3)	7.0736(1)	7.9667(1)	10.8589(2)
*b* (Å)	8.1539(1)	9.7341(2)	12.4910(2)	15.6568(2)	15.4397(2)
*c* (Å)	13.8300(3)	14.3156(3)	12.7785(2)	9.6838(1)	13.1342(2)
*α*(^o^)	83.107(1)	90.0	67.493(1)	90.0	90.0
*β*(^o^)	84.445(1)	99.807(1)	82.002(1)	102.044(1)	93.997(1)
*γ*(^o^)	75.707(1)	90.0	79.475(1)	90.0	90.0
*V* (Å^3^)	785.51(2)	1009.38(5)	1022.53(3)	1181.30(2)	2196.70(6)
*Z*	2	4	2	4	4
*T* (°C)	−113	−113	−113	−113	−113
Radiation	Cu Kα	Cu Kα	Cu Kα	Cu Kα	Cu Kα
*ρ*_calcd (_g cm^−3^_)_	1.736	1.820	1.600	1.779	1.637
*μ* (mm^−1^)	5.500	3.622	2.187	6.840	3.056
Reflections with *I 2σ(I)*	2016	1347	2991	1848	3535
*R* ^a^	0.0362	0.0556	0.0316	0.0271	0.0350
*R_w_* ^a^	0.0787	0.1085	0.0781	0.0703	0.0909

^a^*w* = 1⁄[*σ*^2^(*F*_o_^2^) + (*αP*)^2^ + *bP*] and *P =* [max(*F_o_^2^,*0) + 2*F_c_*^2^]/3, *R* = Σ(||*F*_o_|−|*F*_c_|)/Σ(|*F*_o_||) and *R*_w_ = {Σ[*w*(*F*_o_^2^−*F*_c_^2^)^2^]/Σ[*w*(*F*_o_^2^)^2^]}^1/2^_._

**Table 2:** Selected bond distances (Å) and angles (^o^) for compounds Zn(C_7_H_7_NO_2_)_2_Cl_2_ (**1**), [Zn(C_6_H_9_NO_5_)(H_2_O)]_n_•nH_2_O (**2**), [Zn(C_6_H_9_NO_5_)(C_12_H_8_N_2_)]•4H_2_O (**3**), Zn(C_12_H_8_N_2_)Cl_2_ (**4**), and [Zn(C_12_H_8_N_2_)_2_Cl](NO_3_)•H_2_O (**5**).

Distances (Å)									
1		2		3		4		5	
Zn-O(1)	1.966(2)	Zn-O(4)	2.016(3)	Zn(1)-N(12)	2.086(2)	Zn(1)-N(2)	2.059(2)	Zn-N(21)	2.068(2)
Zn-O(11)	1.967(2)	Zn-O(1)	2.074(3)	Zn(1)-O(3)	2.088(2)	Zn(1)-N(1)	2.078(2)	Zn-N(1)	2.076(2)
Zn-Cl(2)	2.250(1)	Zn-O(1w)	2.108(4)	Zn(1)-O(1)	2.088(2)	Zn(1)-Cl(2)	2.2102(6)	Zn-N(2)	2.148(2)
Zn-Cl(1)	2.269(1)	Zn-O(3)	2.134(3)	Zn(1)-N(1)	2.144(2)	Zn(1)-Cl(1)	2.2190(6)	Zn-N(22)	2.179(2)
		Zn-O(5)	2.145(3)	Zn(1)-N(11)	2.199(2)			Zn-Cl	2.2951(6)
		Zn-N	2.201(3)	Zn(1)-O(5)	2.244(2)				
**Angles (^o^)**									
O(1)-Zn-O(11)	113.52(10)	O(4)-Zn-O(1)	110.84(11)	N(12)-Zn(1)-O(3)	90.01(7)	N(2)-Zn(1)-N(1)	81.49(8)	N(21)-Zn-N(1)	121.09(7)
O(1)-Zn-Cl(2)	105.80(8)	O(4)-Zn-O(1w)	92.99(12)	N(12)-Zn(1)-O(1)	107.62(7)	N(2)-Zn(1)-Cl(2)	108.31(6)	N(21)-Zn-N(2)	96.90(7)
O(11)-Zn-Cl(2)	112.04(9)	O(1)-Zn-O(1w)	88.78(13)	O(3)-Zn(1)-O(1)	101.41(6)	N(1)-Zn(1)-Cl(2)	125.83(6)	N(1)-Zn-N(2)	78.94(7)
O(1)-Zn-Cl(1)	111.22(9)	O(4)-Zn-O(3)	93.25(11)	N(12)-Zn(1)-N(1)	171.07(8)	N(2)-Zn(1)-Cl(1)	115.77(5)	N(21)-Zn-N(22)	78.72(7)
O(11)-Zn-Cl(1)	104.54(8)	O(1)-Zn-O(3)	155.57(11)	O(3)-Zn(1)-N(1)	82.41(6)	N(1)-Zn(1)-Cl(1)	107.11(5)	N(1)-Zn-N(22)	98.24(7)
Cl(2)-Zn-Cl(1)	109.80(4)	O(1w)-Zn-O(3)	85.85(14)	O(1)-Zn(1)-N(1)	78.66(7)	Cl(2)-Zn(1)-Cl(1)	114.40(2)	N(2)-Zn-N(22)	172.72(7)
		O(4)-Zn-O(5)	88.91(12)	N(12)-Zn(1)-N(11)	78.29(7)			N(21)-Zn-Cl	118.31(5)
		O(1)-Zn-O(5)	96.16(12)	O(3)-Zn(1)-N(11)	166.26(7)			N(1)-Zn-Cl	120.59(5)
		O(1w)-Zn-O(5)	173.70(14)	N(1)-Zn(1)-N(11)	108.58(7)			N(2)-Zn-Cl	95.16(5)
		O(3)-Zn-O(5)	88.06(12)	N(12)-Zn(1)-O(5)	95.65(7)			N(22)-Zn-Cl	92.04(5)
		O(4)-Zn-N	166.17(12)	O(3)-Zn(1)-O(5)	87.80(6)				
		O(1)-Zn-N	78.18(11)	O(1)-Zn(1)-O(5)	154.81(7)				
		O(1w)-Zn-N	97.77(13)	N(1)-Zn(1)-O(5)	79.42(7)				
		O(3)-Zn-N	78.96(11)	N(11)-Zn(1)-O(5)	86.24(6)				
		O(5)-Zn-N	79.49(12)						

**Table 3:** Primers used in RT-PCR.

Gene	Primer (Forward (F); Reverse (R))	Sequence
*GLUT 4*	F	5′-AACCAGCATCTTCGAGTCGG-3′
	R	5′-TAAGAGCACCGAGACCAACG-3′
*GLUT 1*	F	5′-CTCACCACGCTTTGGTCTCT-3′
	R	5′-CGCCTGCCAAAGCGATTAAC-3′
*PPAR-γ*	F	5′-GTCAGCGACTGGGACTTTTC-3′
	R	5′-CGAGGACATCCAAGACAACC-3′

## Data Availability

The data presented in this study are available on request from the corresponding author.

## Supplementary Materials

The following are available online at https://www.mdpi.com/article/10.3390/ijms22136757/s1. X-ray crystal crystallographic files, in CIF format (CCDC 2076934 (**1**), 2076935 (**2**), 2076936 (**3**), 2076937 (**4**), and 2076938(**5**)), and listings of positional and thermal parameters and H-bond distances and angles for **1**–**5**.

## Abbreviation List

ANOVA	One-way analysis of variance
DM	Diabetes mellitus
DMEM	Dulbecco’s modified Eagle’s medium
FBS	Fetal Bovine Serum
GLUT 1,4	Glucose transporter 1,4
PPAR-γ	Peroxisome proliferator-activated receptor gamma
SEM	Standard error mean
LMCT	Ligand-to-metal-charge-transfer
ADA	American Diabetes Association
ESI-MS	Electrospray ionization mass spectrometry
TGA	Thermogravimetric analysis
RLU	Relative luminescence units
MAPK	Mitogen-activated protein kinase
PI3K	Phosphoinositide 3-kinase
NMR	Nuclear magnetic resonance
