# Supplementary material for: Evaluation of Insulin-Like Activity of Novel Zinc Metal–Organics toward Adipogenesis Signaling
Source: Int J Mol Sci. 2021 Jun 23;22(13):6757. doi: 10.3390/ijms22136757 (PMC8268141; doi:10.3390/ijms22136757)
Supplement: Supplementary file 1 [file ijms-22-06757-s001.zip › ijms-1239064 resubmitted supplementary.pdf]

Supplementary Information

Evaluation of insulin-like activity of novel zinc metal-organics toward adipogenesis signaling.

C. Gabriel,[a,b,†] O. Tsave,[a,†] M.P. Yavropoulou,[c] T. Architektonidis,[a] C. P. Raptopoulou,[d*] V. Psycharis,[d] A. Salifoglou[a]

*Author to whom correspondence should be addressed.

Tel: +30-2310-996-179, E-mail: salif@auth.gr

[a] School of Chemical Engineering, Laboratory of Inorganic Chemistry and Advanced Materials, Aristotle University of Thessaloniki, Thessaloniki 54124 Greece

[b] Center for Research of the Structure of Matter, Magnetic Resonance Laboratory, School of Chemical Engineering, Aristotle University of Thessaloniki, Thessaloniki 54124, Greece

[c] Endocrinology Unit, 1st Department of Propaedeutic and Internal Medicine, Medical School, National and Kapodistrian University of Athens, Greece

[d] Institute of Nanoscience and Nanotechnology, NCSR "Demokritos", Aghia Paraskevi 15310, Attiki, Greece

[†] These authors contributed equally to this work.

Table S1. Intermolecular interactions in the structures of **1-5**.

Interaction	D···A (Å)	H···A (Å)	D-H···A (°)	Symmetry operation
**1**				
C7-H7B···Cl2	3.747	2.804	161.4	1+x, 1+y, z
C14-H14···Cl1	3.612	2.951	127.7	-2-x, -y, 2-z intra layer
C17-H17A···Cl1	3.865	2.919	162.6	-2-x, 1-y, 2-z intra chain
C4-H4···O12	3.032	2.401	123.6	-1-x, 1-y, 1-z intra chain
C5-H5···O12	3.082	2.499	119.7	-1-x, 1-y, 1-z
C14-H14···O2	3.045	2.442	121.2	-2-x, 1-y, 2-z
C15-H15···O2	3.049	2.445	121.3	-2-x, 1-y, 2-z
**2**				
O5-H5O···O2	2.627	1.804	172.7	-1+x, y, z
O1w-H1w1···O2	2.776	1.901	167.3	1-x, 0.5+y, 0.5-z
O1w-H2w1···O2w	2.660	1.938	163.7	1+x, y, z
O2w-H1w2···O4	2.775	2.014	159.5	x, y, z
O2w-H2w2···O1	2.836	2.090	163.7	-x, 0.5+y, 0.5-z
C5-H5B···O5	3.401	2.664	138.5	-x, -y, -z
**3**				
O5-H5O···O4	2.706	1.916	169.3	1-x, -y, 1-z
O1w-H1w1···O2	2.766	1.973	171.2	-x, 1-y, 1-z
O1w-H2w1···O2	2.849	2.049	178.6	x, y, z
O2w-H1w2···O2	2.800	1.997	170.2	x, y, z
O2w-H2w2···O3w	2.844	2.055	157.3	-x, 1-y, 1-z
O3w-H1w3···O1w	2.753	1.990	172.5	1+x, y, z
O3w-H2w3···O4w	2.831	2.023	169.2	1-x, 1-y, -z
O4w-H1w4···O3w	2.810	1.953	168.7	x, y, z
O4w-H2w4···O4	2.774	2.039	172.2	x, y, z
C1-H1A···O2	3.366	2.551	145.9	-x, 1-y, 1-z
C1-H1B···O2w	3.258	2.314	165.6	1+x, y, z
C5-H5B···O2w	3.417	2.612	144.6	1+x, y, z
C11-H11···O2w	3.433	2.515	157.7	1+x, y, z

C13-H13···O5	3.436	2.654	155.6	1-x, -y, 2-z
C15-H15···O1	3.260	2.591	129.7	-x, -y, 2-z
C19-H19···O1w	3.415	2.655	137.7	-x, -y, 1-z
**4**				
C5-H5···Cl1	3.493	2.778	136.2	-x, -y, 1-z
C8-H8···Cl1	3.647	2.836	153.8	0.5-x, -0.5+y, 0.5-z
C9-H9···Cl1	3.552	2.856	136.2	0.5+x, 0.5-y, -0.5+z
**5**				
C3-H3···Cl	3.538	2.869	133.8	0.5-x, -y, -z Intralayer
C6-H6···Cl	3.672	2.886	132.6	0.5+x, 0.5-y, -0.5+z
C23-H23···Cl	3.665	2.909	141.0	0.5-x, 0.5-y, 0.5-z Intralayer
O1w-H1w1···Cl	3.270	2.452	177.5	0.5+x, 0.5-y, 0.5+z
O1w-H2w1···O32	2.842	2.130	176.5	-0.5+x, 0.5-y, 0.5+z

Figures

Fig. S1: ESI Spectra of **1(A)-5(E)** in methanol.

Fig. S2: ^{13}C-NMR solution spectra (in D_2O) of compounds **1**, **2, 3, 4**, and **5** (**2A**, **2B, 2C, 2D** and **2E**, respectively), with shown peak assignments.

Fig. S3: ^{1}H-NMR solution spectra (in D_2O) of compounds **1**, **2, 3, 4**, and **5** (**3A**, **3B, 3C, 3D** and **3E**, respectively), with shown peak assignments.

Fig. S4: TGA diagrams of **1(A)-5(E).**

Fig. S5: Luminescence diagrams of **1(A)-5(E)** in the solid state.

Fig. S6: Cell migration of 3T3-L1 cells: **A**) control (t=0), **B**) control after 24 h, **C**) cells treated with 10 μM of **1**, **D**) cells treated with 10 μM of **2**, **E**) cells treated with 10 μM of **3**, **F**) cells treated with 10 μM of **4**, and **G**) cells treated with 10 μM of **5** after 24 h using an *in vitro* standard scratch assay.

Fig. S7 Representative micrographs of culture samples of 3T3-L1 in the presence of **1**-**5** at concentrations of 10 and 50 μM, 24 h post treatment in pre-adipocytes.

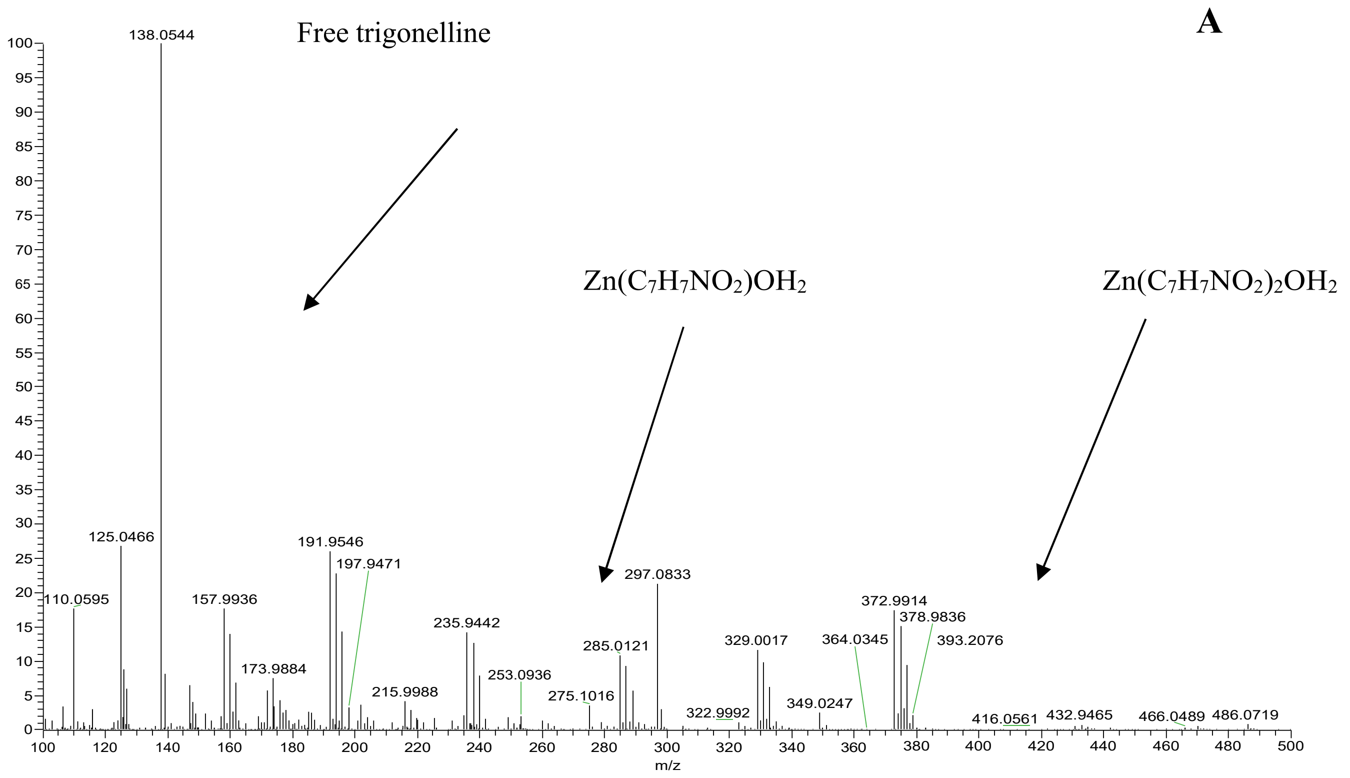
A
Free trigonelline
Zn(C7H7NO2)OH2
Zn(C7H7NO2)2OH2
138.0544
125.0466
110.0595
157.9936
173.9884
191.9546
197.9471
215.9988
235.9442
253.0936
275.1016
285.0121
297.0833
322.9992
329.0017
349.0247
364.0345
372.9914
378.9836
393.2076
416.0561
432.9465
466.0489
486.0719
m/z

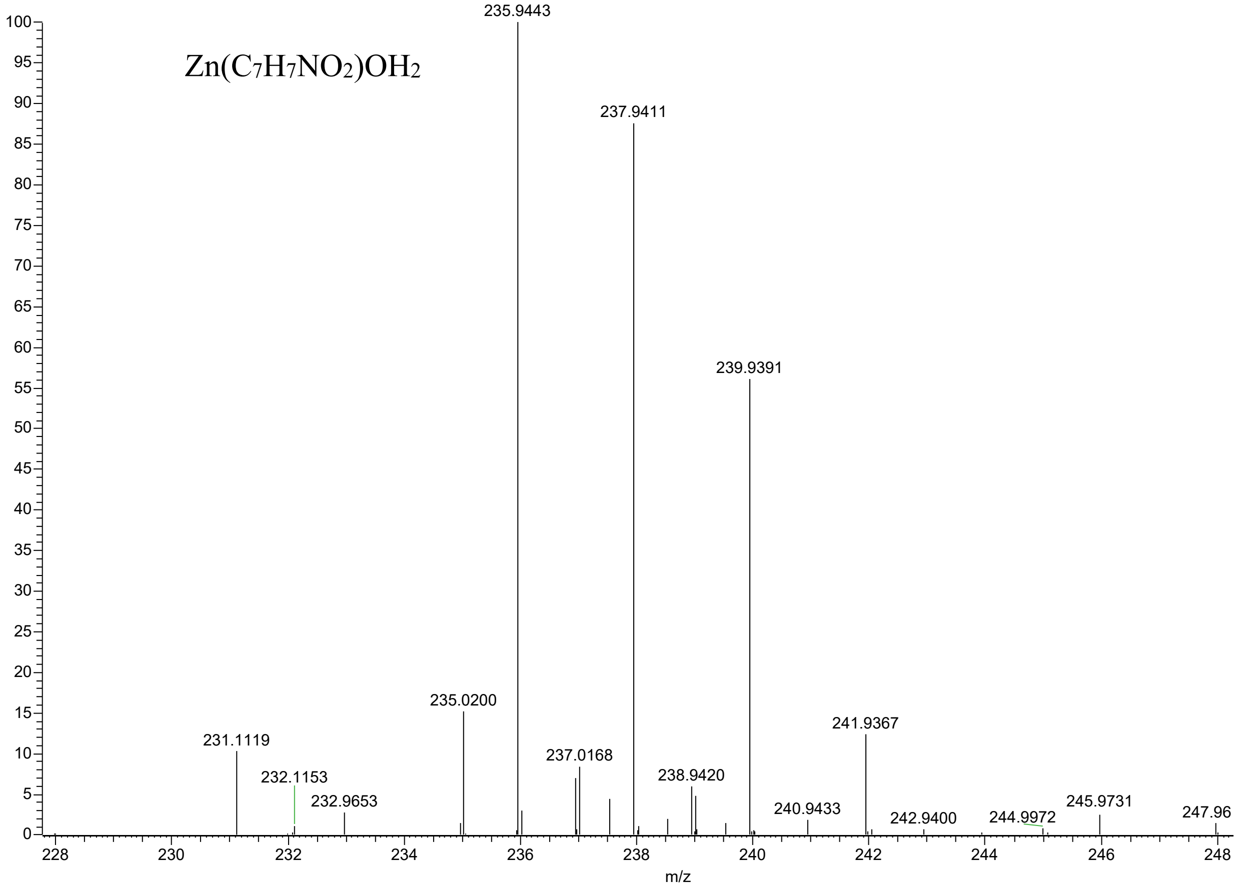

$Zn(C_7H_7NO_2)_2OH_2$

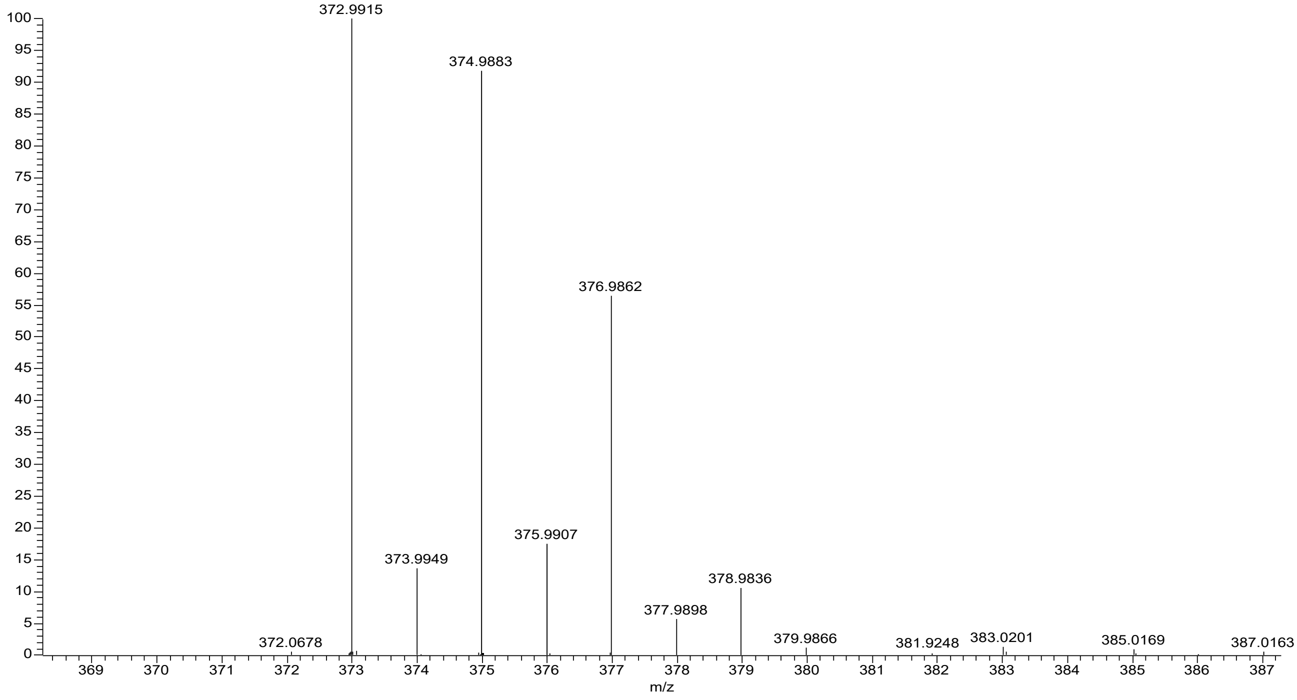

B

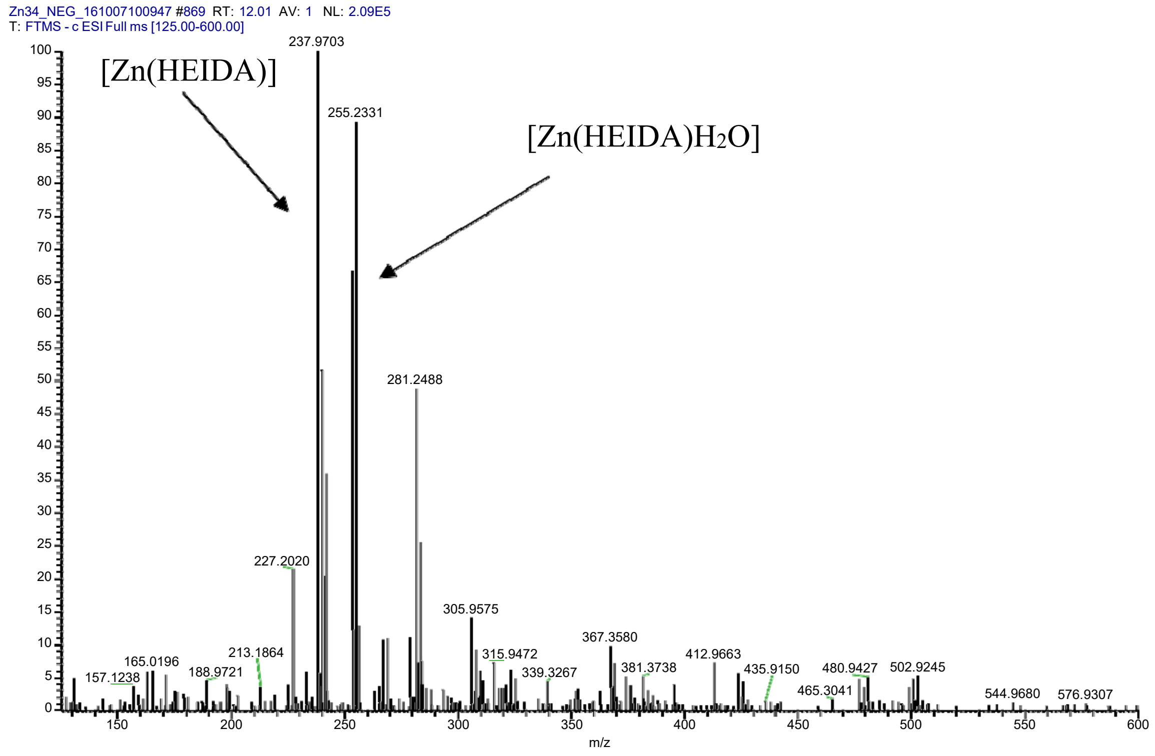
Zn34_NEG_161007100947 #869 RT: 12.01 AV: 1 NL: 2.09E5
T: FTMS - c ESI Full ms [125.00-600.00]
[Zn(HEIDA)]
[Zn(HEIDA)H2O]
237.9703
255.2331
281.2488
227.2020
305.9575
367.3580
213.1864
165.0196
315.9472
412.9663
188.9721
339.3267
381.3738
435.9150
480.9427
502.9245
157.1238
465.3041
544.9680
576.9307
m/z

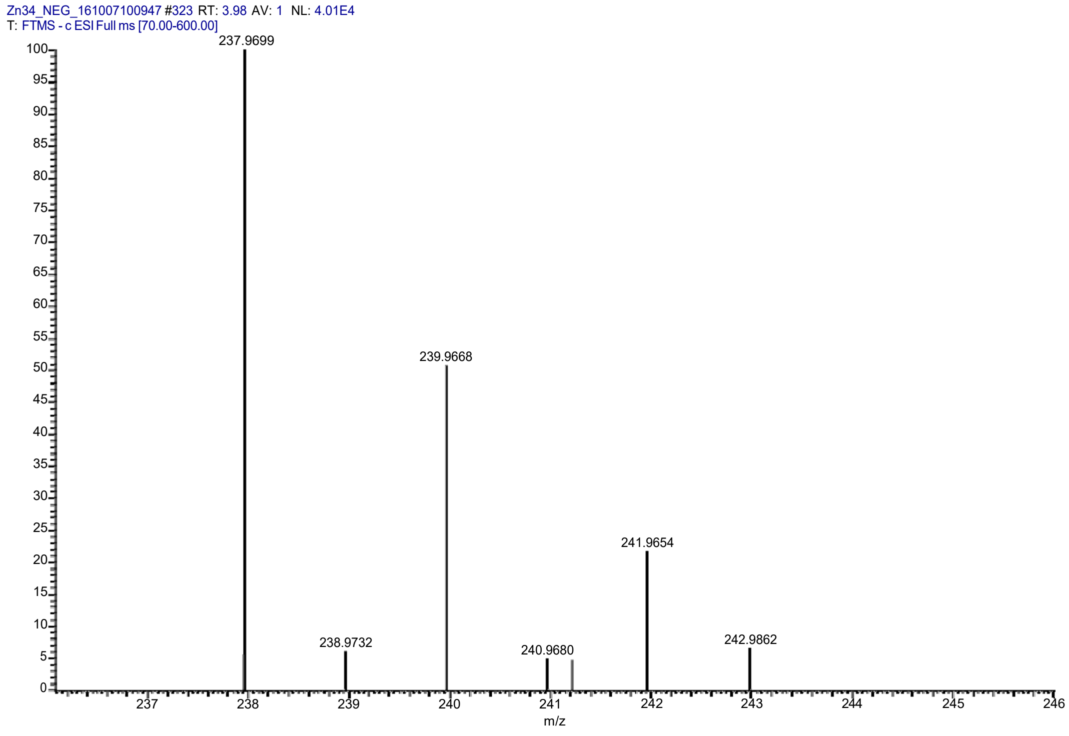
Zn34_NEG_161007100947 #323 RT: 3.98 AV: 1 NL: 4.01E4
T: FTMS - c ESI Full ms [70.00-600.00]
237.9699
239.9668
241.9654
238.9732
240.9680
242.9862
m/z

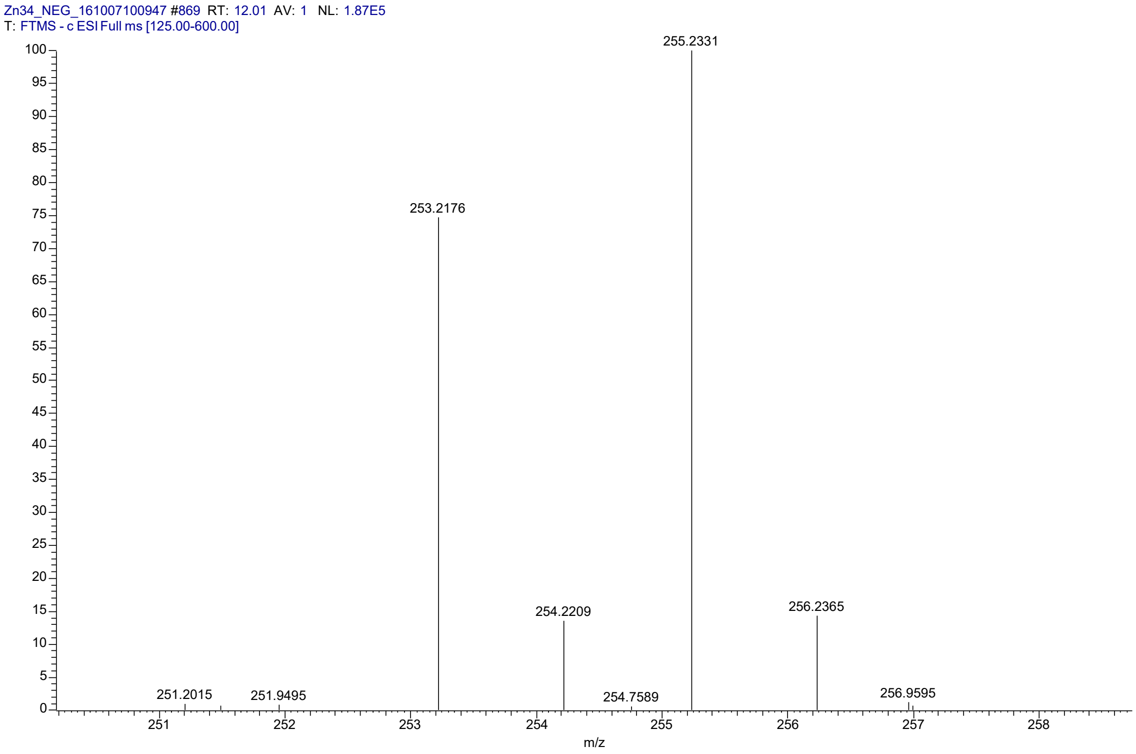
Zn34_NEG_161007100947 #869 RT: 12.01 AV: 1 NL: 1.87E5
T: FTMS - c ESI Full ms [125.00-600.00]
255.2331
253.2176
254.2209
256.2365
251.2015
251.9495
254.7589
256.9595
m/z

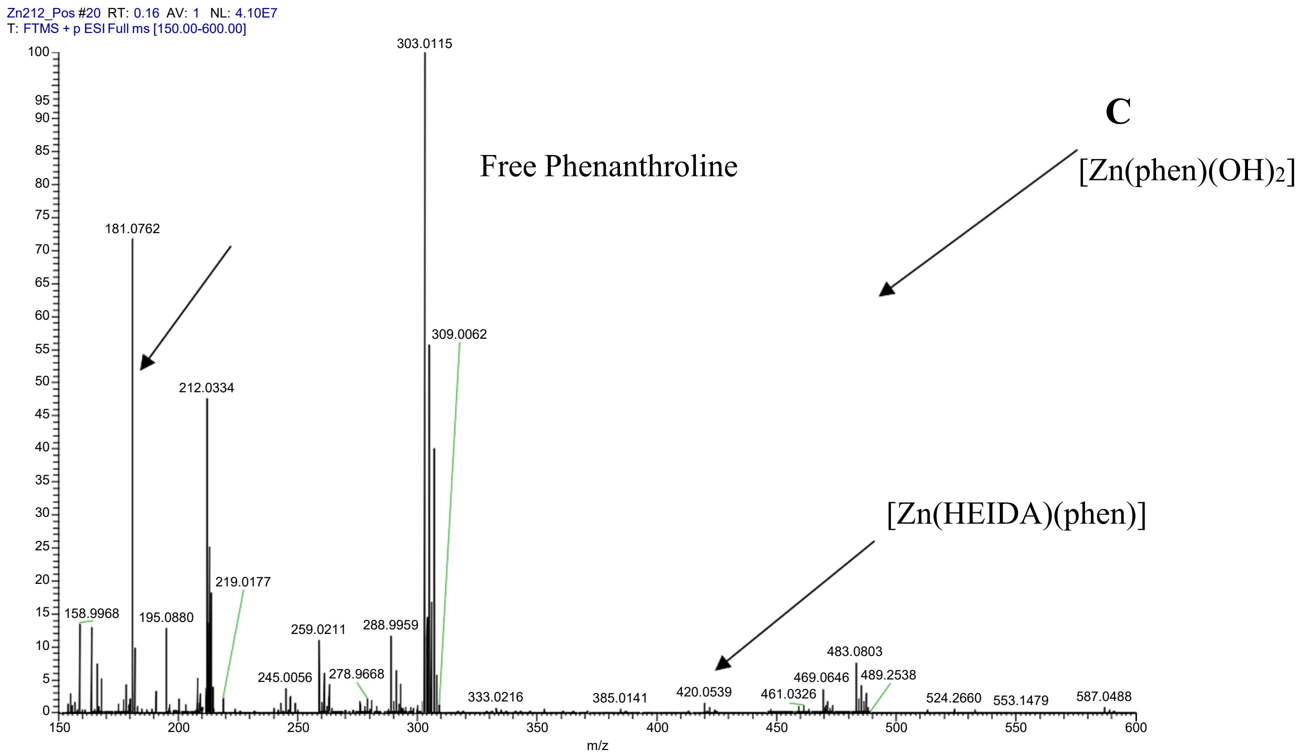
Zn212_Pos #20 RT: 0.16 AV: 1 NL: 4.10E7
T: FTMS + p ESI Full ms [150.00-600.00]
C
Free Phenanthroline
[Zn(phen)(OH)2]
[Zn(HEIDA)(phen)]
303.0115
181.0762
309.0062
212.0334
219.0177
158.9968
195.0880
259.0211
288.9959
245.0056
278.9668
333.0216
385.0141
420.0539
461.0326
469.0646
483.0803
489.2538
524.2660
553.1479
587.0488
m/z

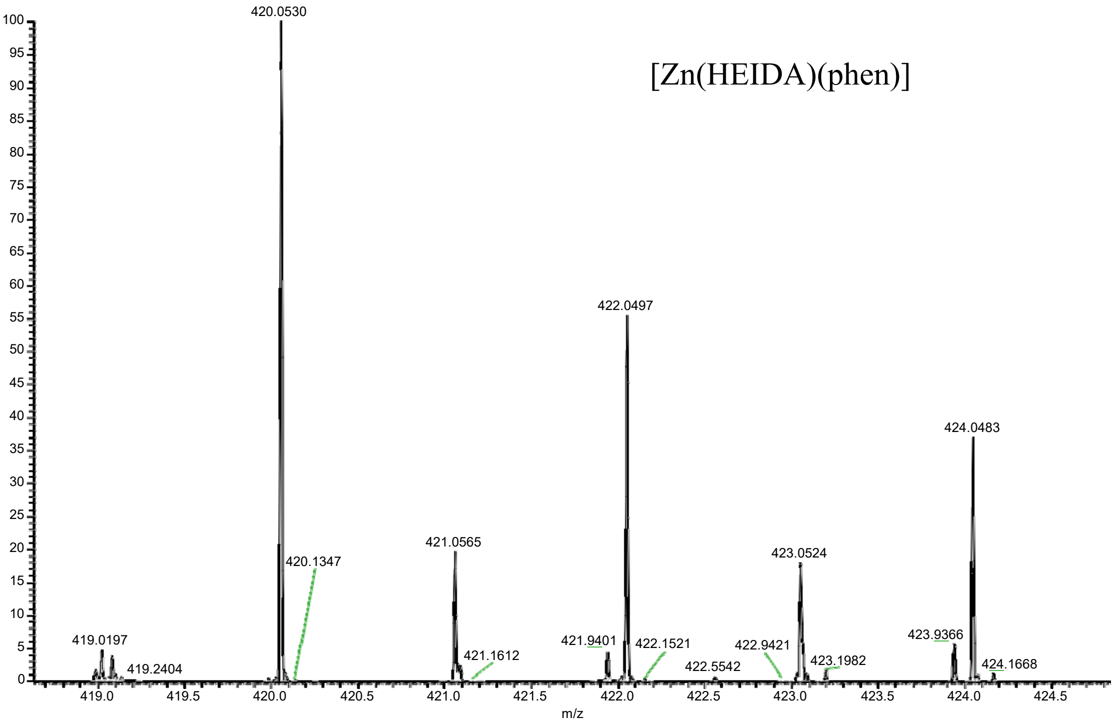
[Zn(HEIDA)(phen)]
420.0530
422.0497
424.0483
421.0565
420.1347
423.0524
419.0197
419.2404
421.1612
421.9401
422.1521
422.5542
422.9421
423.1982
423.9366
424.1668
m/z

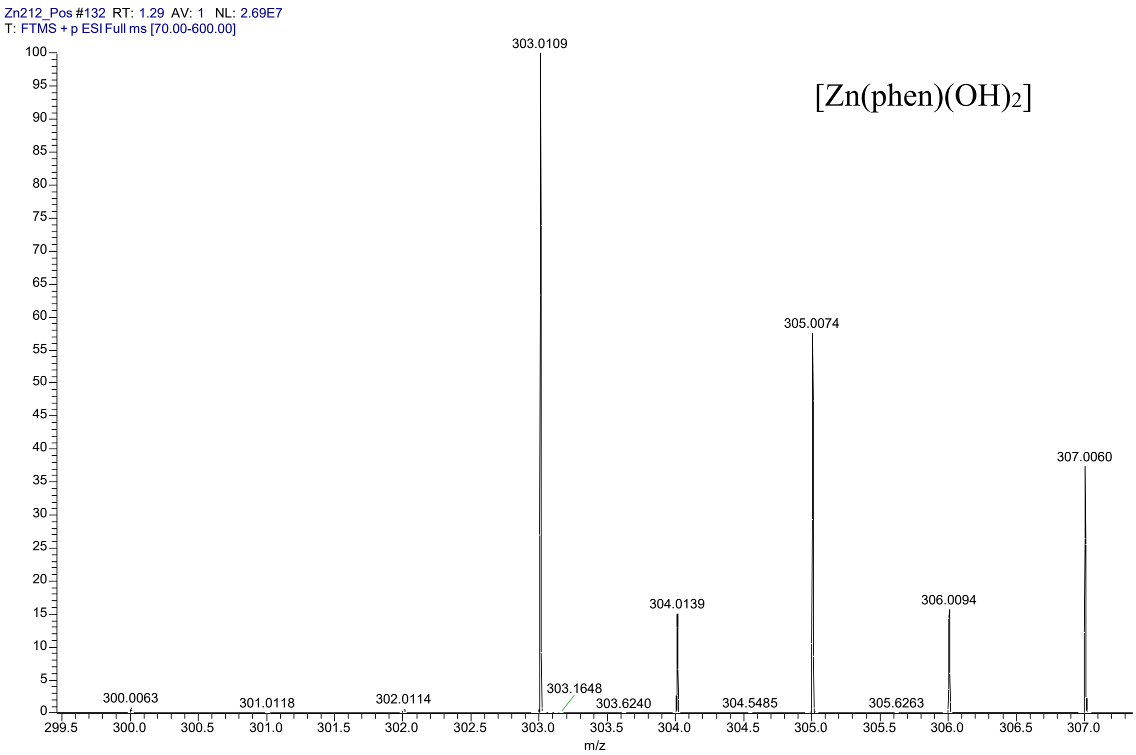
Zn212_Pos #132 RT: 1.29 AV: 1 NL: 2.69E7
T: FTMS + p ESI Full ms [70.00-600.00]
[Zn(phen)(OH)2]
303.0109
305.0074
307.0060
304.0139
306.0094
300.0063
301.0118
302.0114
303.1648
303.6240
304.5485
305.6263
m/z

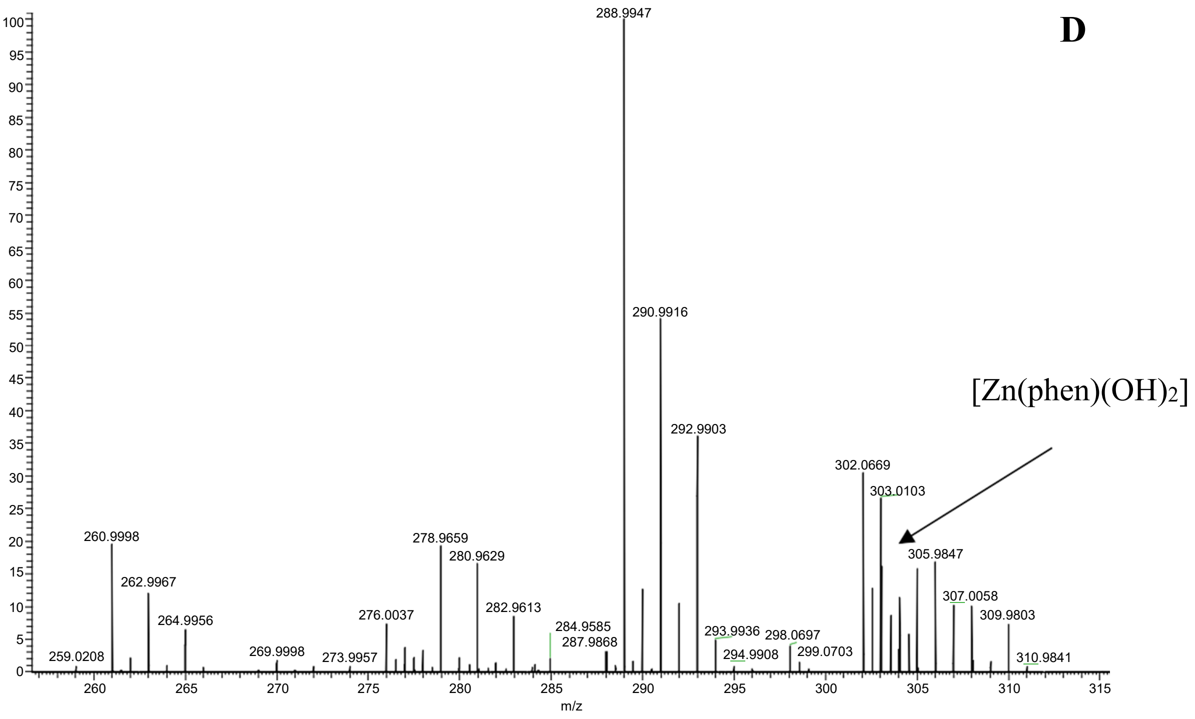
D
[Zn(phen)(OH)2]
288.9947
290.9916
292.9903
302.0669
303.0103
305.9847
307.0058
309.9803
310.9841
260.9998
262.9967
264.9956
259.0208
269.9998
273.9957
276.0037
278.9659
280.9629
282.9613
284.9585
287.9868
293.9936
294.9908
298.0697
299.0703
m/z

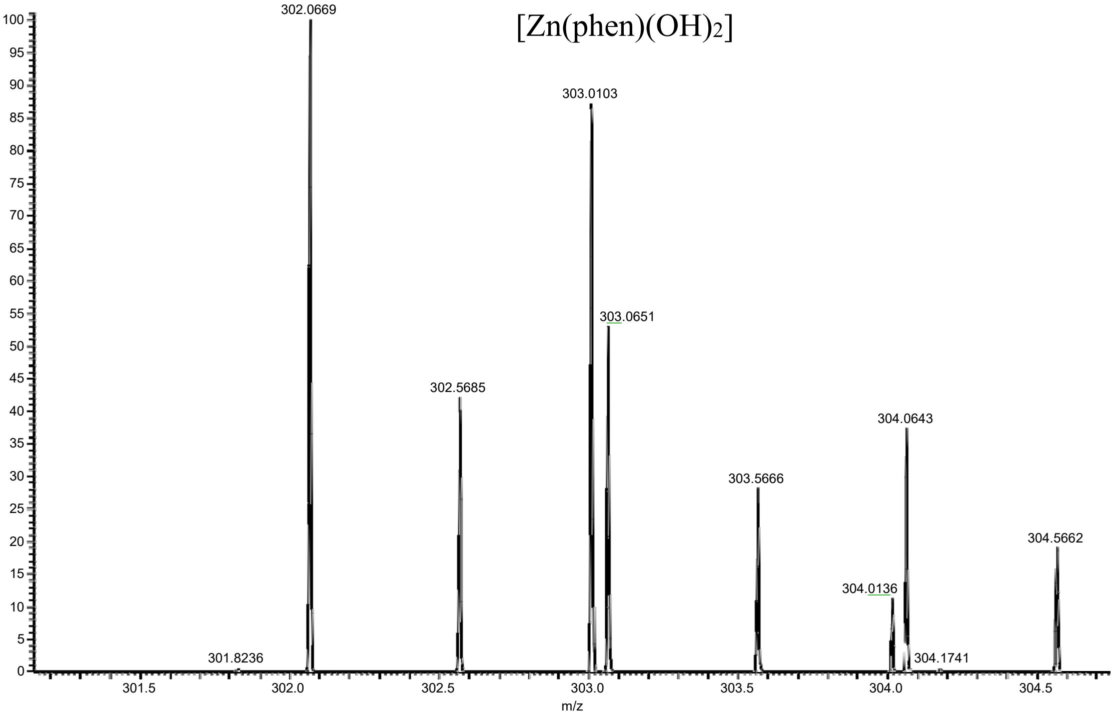
[Zn(phen)(OH)2]
302.0669
303.0103
303.0651
302.5685
304.0643
303.5666
304.5662
304.0136
301.8236
304.1741
m/z

E

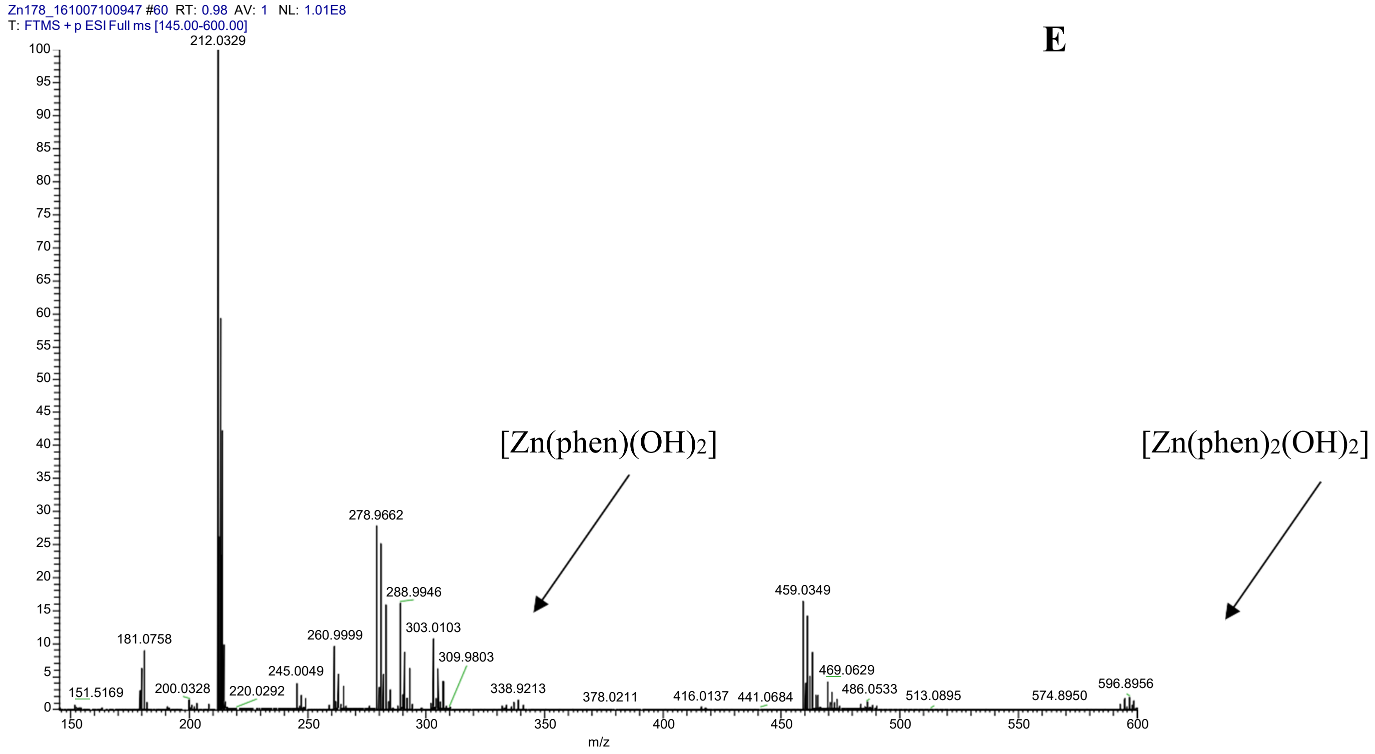
Zn178_161007100947 #60 RT: 0.98 AV: 1 NL: 1.01E8
T: FTMS + p ESI Full ms [145.00-600.00]
212.0329
181.0758
151.5169
200.0328
220.0292
245.0049
260.9999
278.9662
288.9946
303.0103
309.9803
338.9213
378.0211
416.0137
441.0684
459.0349
469.0629
486.0533
513.0895
574.8950
596.8956
m/z
[Zn(phen)(OH)2]
[Zn(phen)2(OH)2]

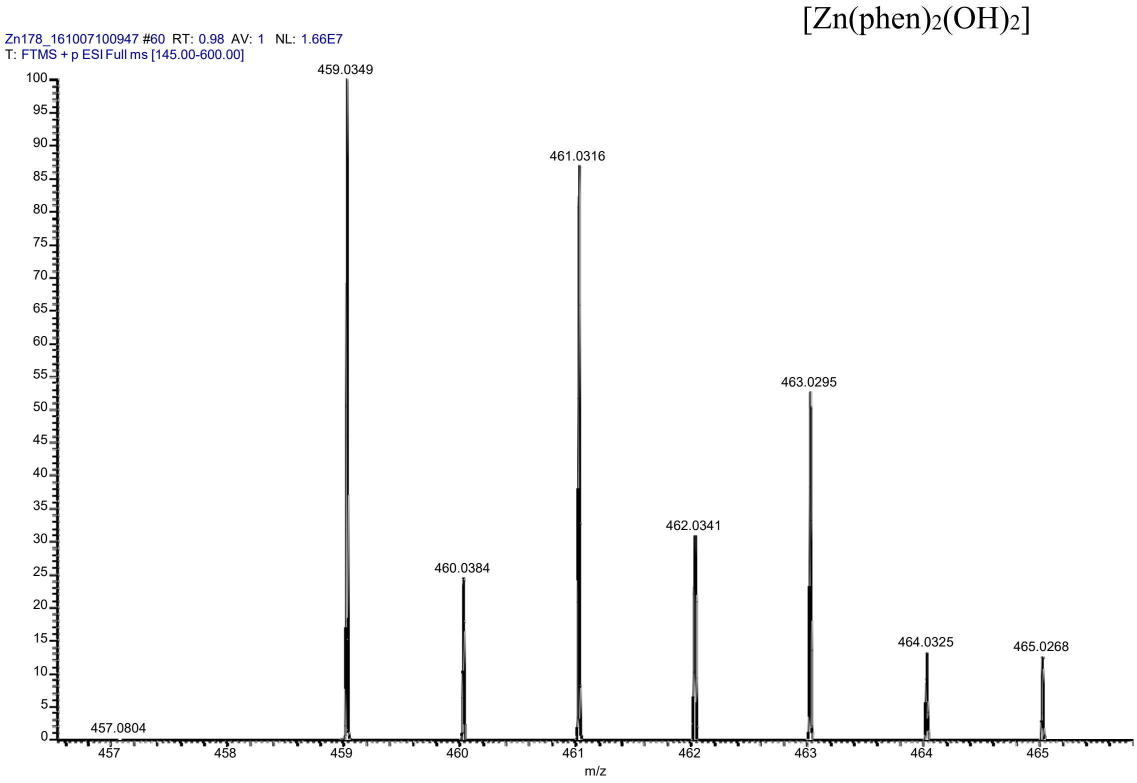
[Zn(phen)2(OH)2]
Zn178_161007100947 #60 RT: 0.98 AV: 1 NL: 1.66E7
T: FTMS + p ESI Full ms [145.00-600.00]
459.0349
461.0316
463.0295
462.0341
460.0384
464.0325
465.0268
457.0804
m/z

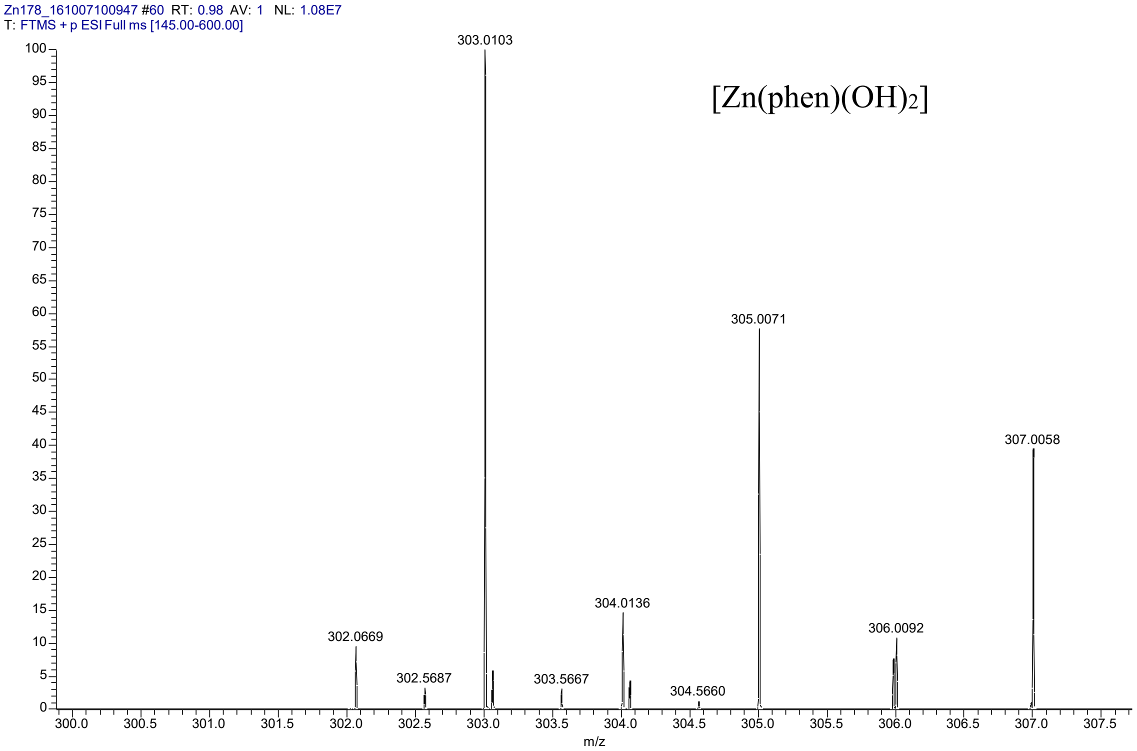
Zn178_161007100947 #60 RT: 0.98 AV: 1 NL: 1.08E7
T: FTMS + p ESI Full ms [145.00-600.00]
[Zn(phen)(OH)2]
303.0103
305.0071
307.0058
304.0136
306.0092
302.0669
302.5687
303.5667
304.5660
m/z

Figure S1

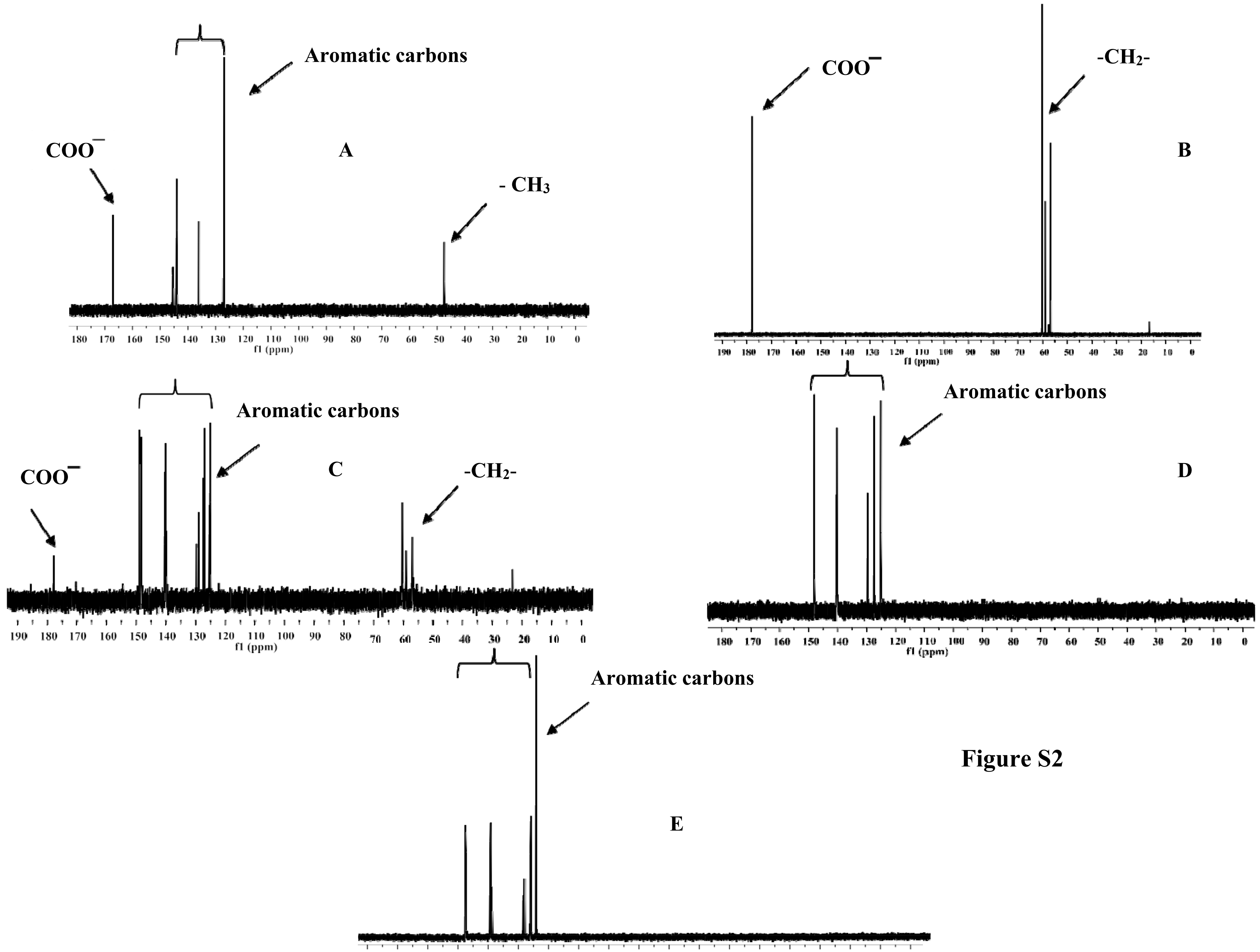
Aromatic carbons
COO‾
A
- CH3
f1 (ppm)
COO‾
-CH2-
B
f1 (ppm)
Aromatic carbons
COO‾
C
-CH2-
f1 (ppm)
Aromatic carbons
D
f1 (ppm)
Aromatic carbons
E

Figure S2

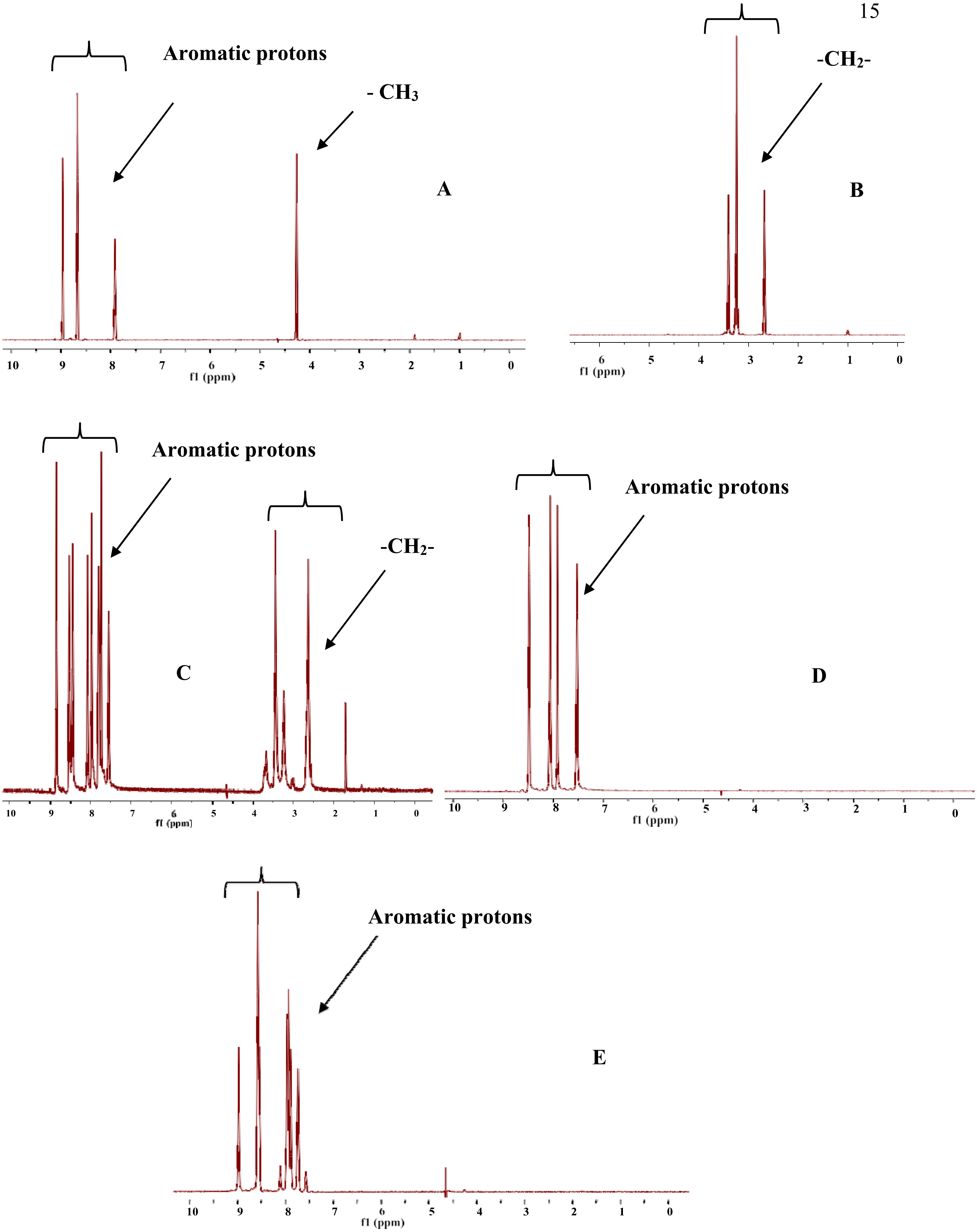
Aromatic protons
- CH3
A
f1 (ppm)
-CH2-
B
f1 (ppm)
Aromatic protons
-CH2-
C
f1 (ppm)
Aromatic protons
D
f1 (ppm)
Aromatic protons
E
f1 (ppm)

Figure S3

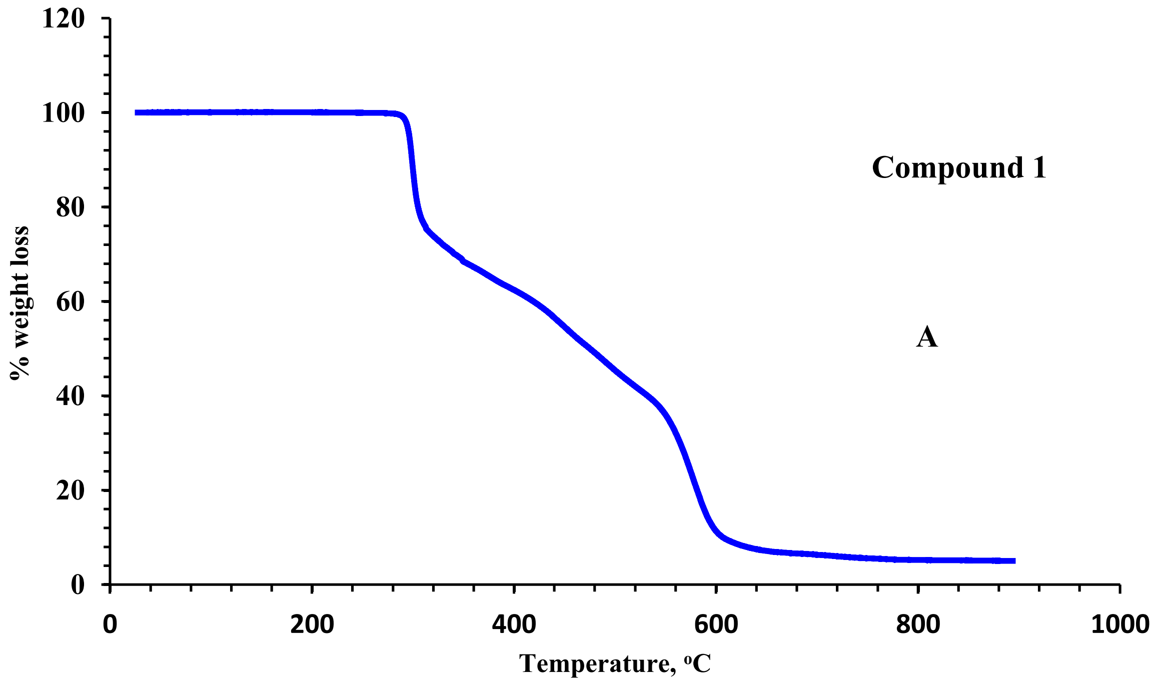
120
100
80
60
40
20
0
% weight loss
0
200
400
600
800
1000
Temperature, oC
Compound 1
A

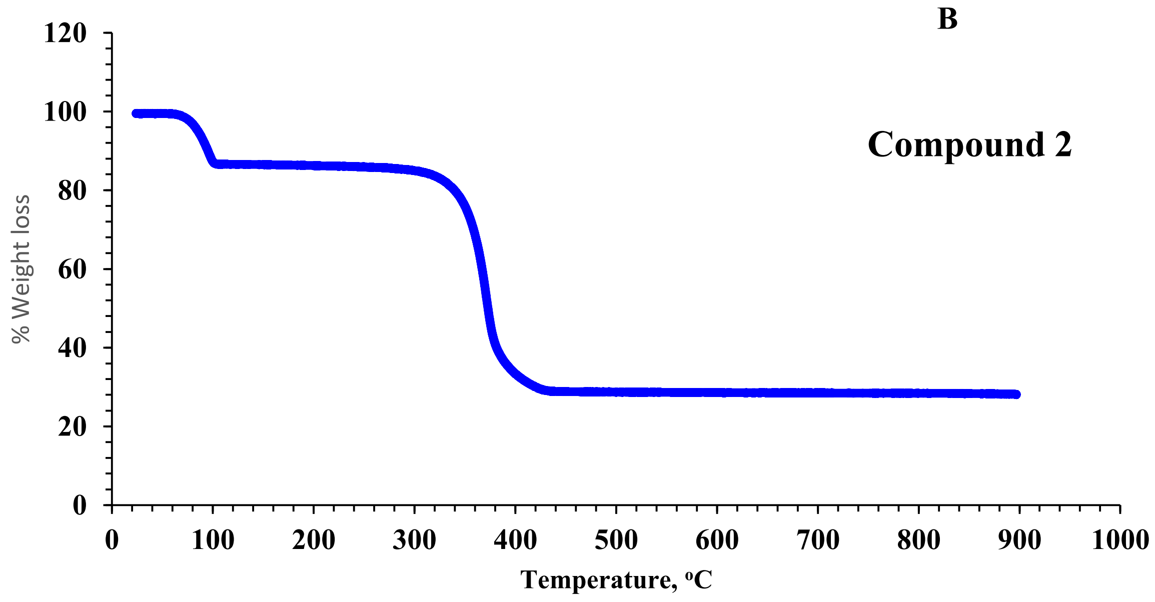
B
120
100
80
60
40
20
0
% Weight loss
0
100
200
300
400
500
600
700
800
900
1000
Temperature, oC
Compound 2

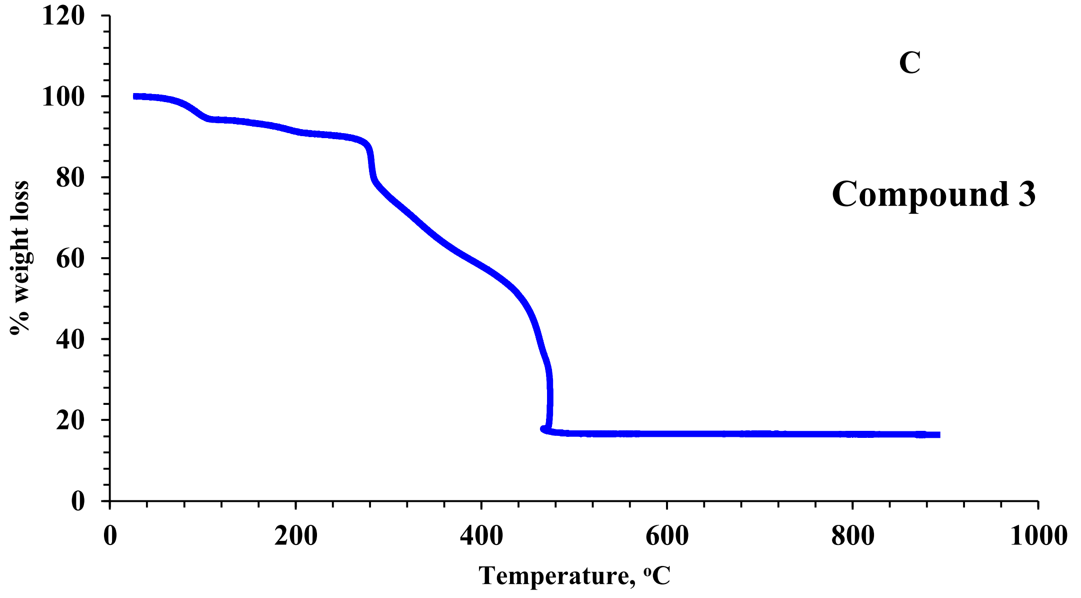
C
Compound 3
% weight loss
120
100
80
60
40
20
0
0
200
400
600
800
1000
Temperature, °C

D

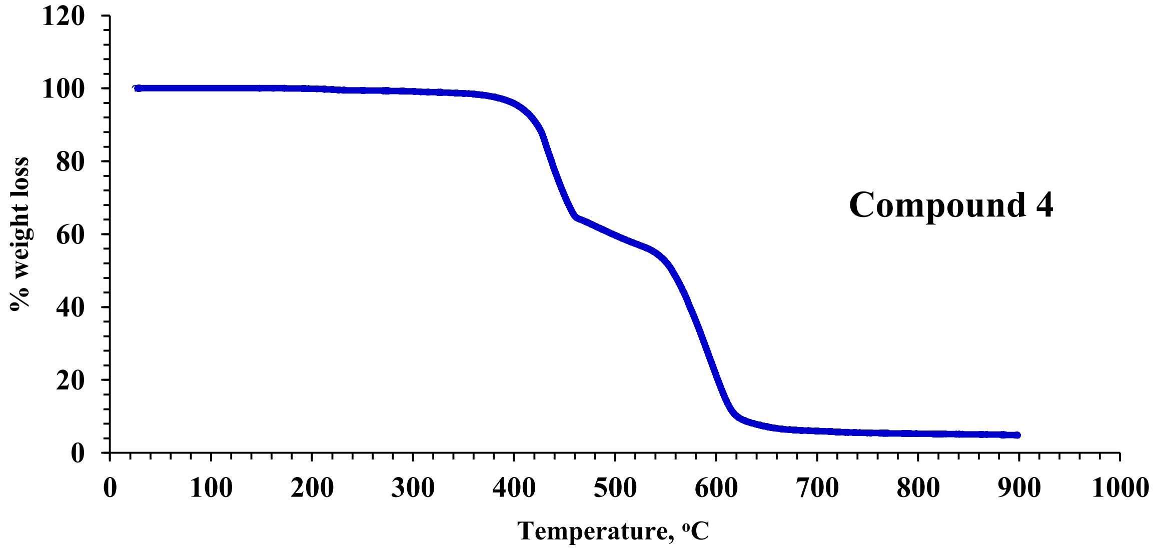
Compound 4
% weight loss
120
100
80
60
40
20
0
0
100
200
300
400
500
600
700
800
900
1000
Temperature, °C

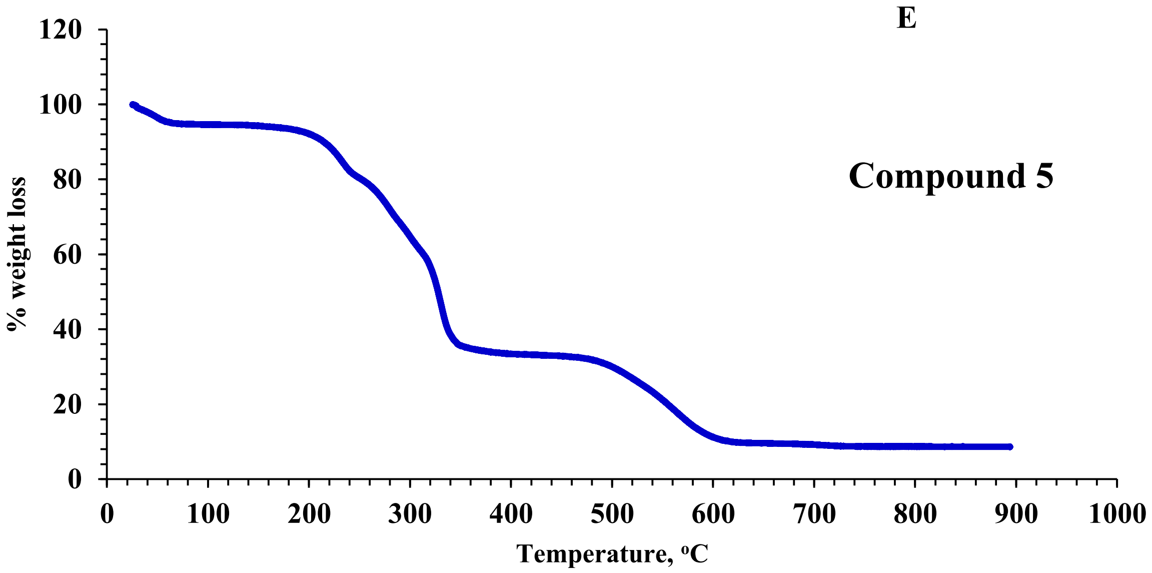
E
Compound 5
% weight loss
0
20
40
60
80
100
120
0
100
200
300
400
500
600
700
800
900
1000
Temperature, °C

Figure S4

A

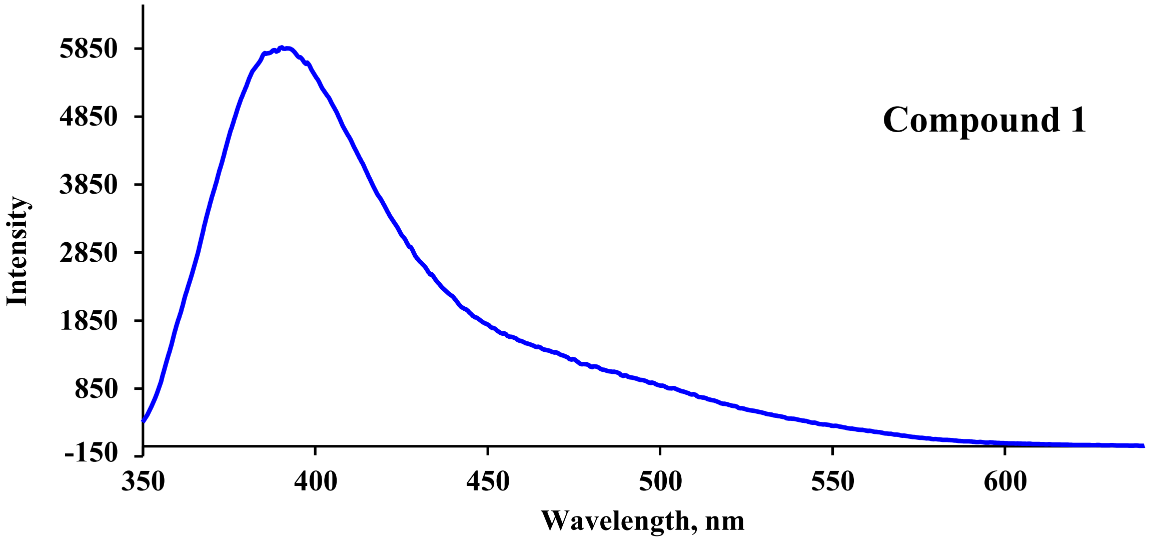
Compound 1
Intensity
5850
4850
3850
2850
1850
850
-150
350
400
450
500
550
600
Wavelength, nm

B

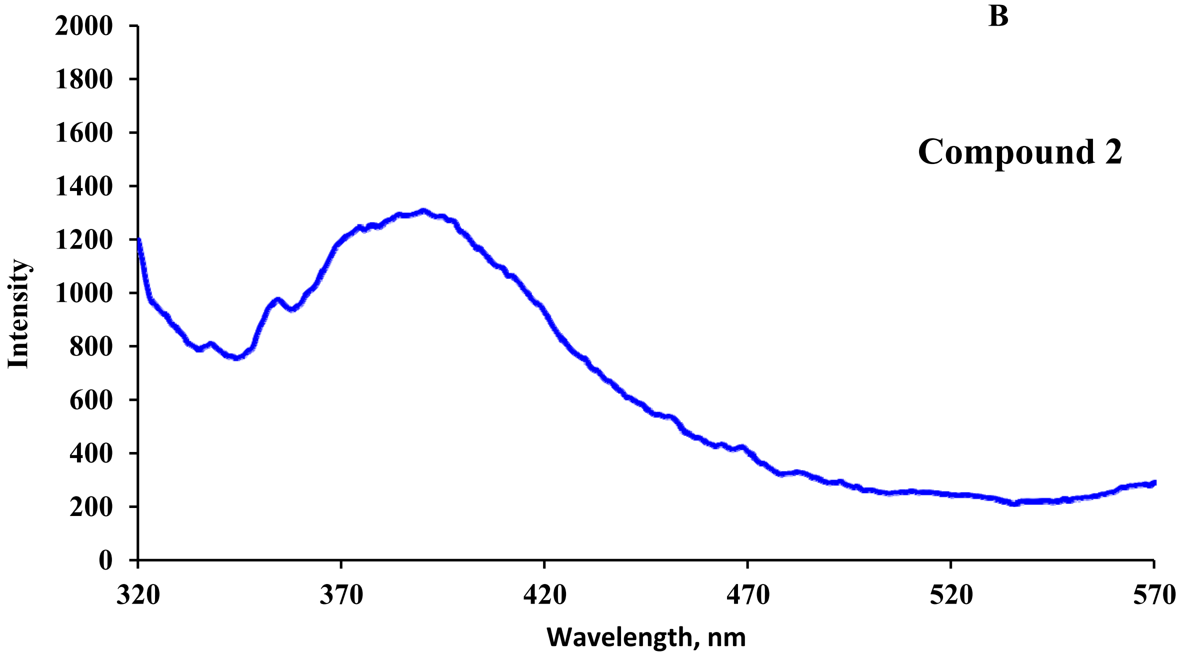
Compound 2
Intensity
2000
1800
1600
1400
1200
1000
800
600
400
200
0
320
370
420
470
520
570
Wavelength, nm

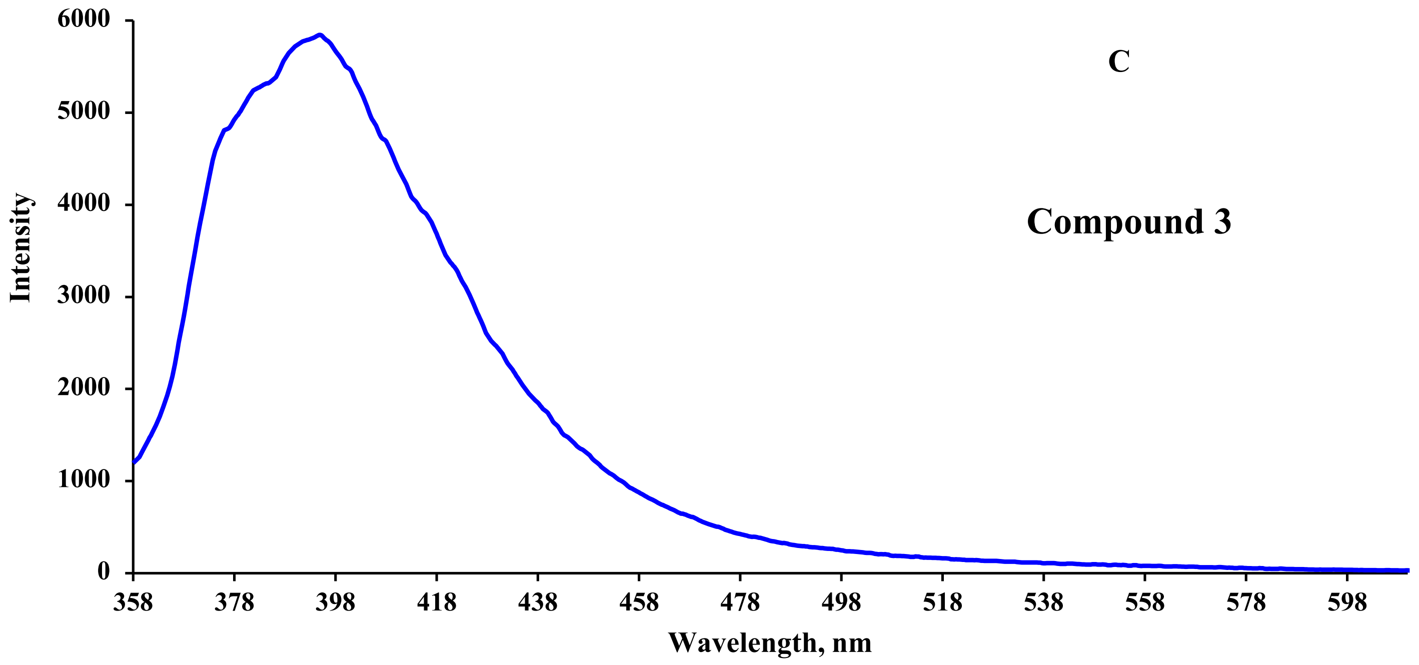
C
Compound 3
Intensity
6000
5000
4000
3000
2000
1000
0
358
378
398
418
438
458
478
498
518
538
558
578
598
Wavelength, nm

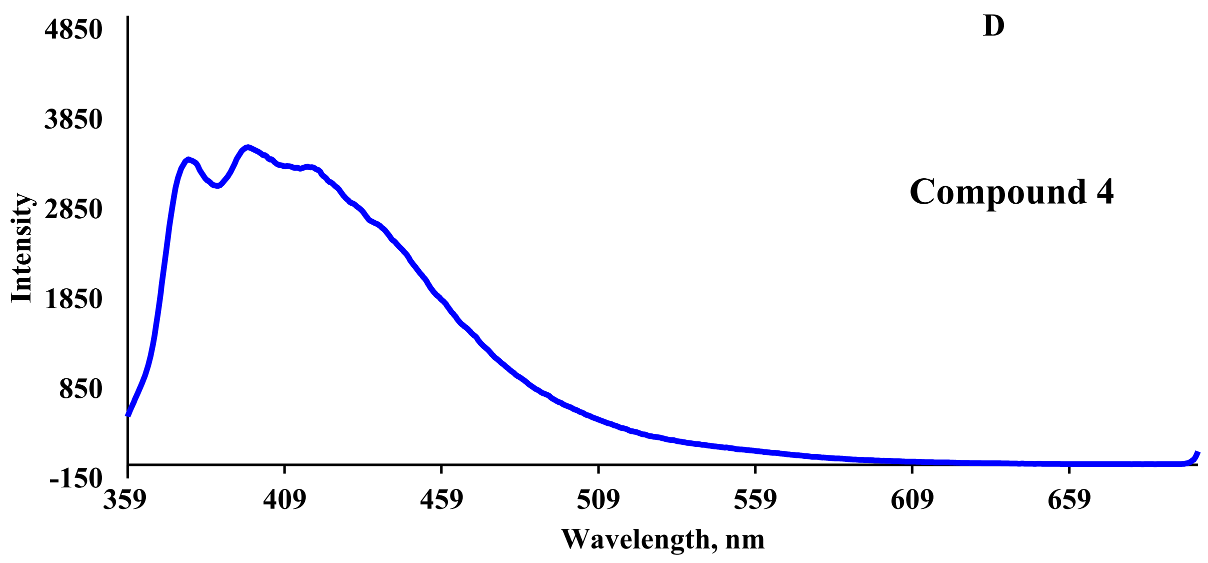
D
Compound 4
Intensity
4850
3850
2850
1850
850
-150
359
409
459
509
559
609
659
Wavelength, nm

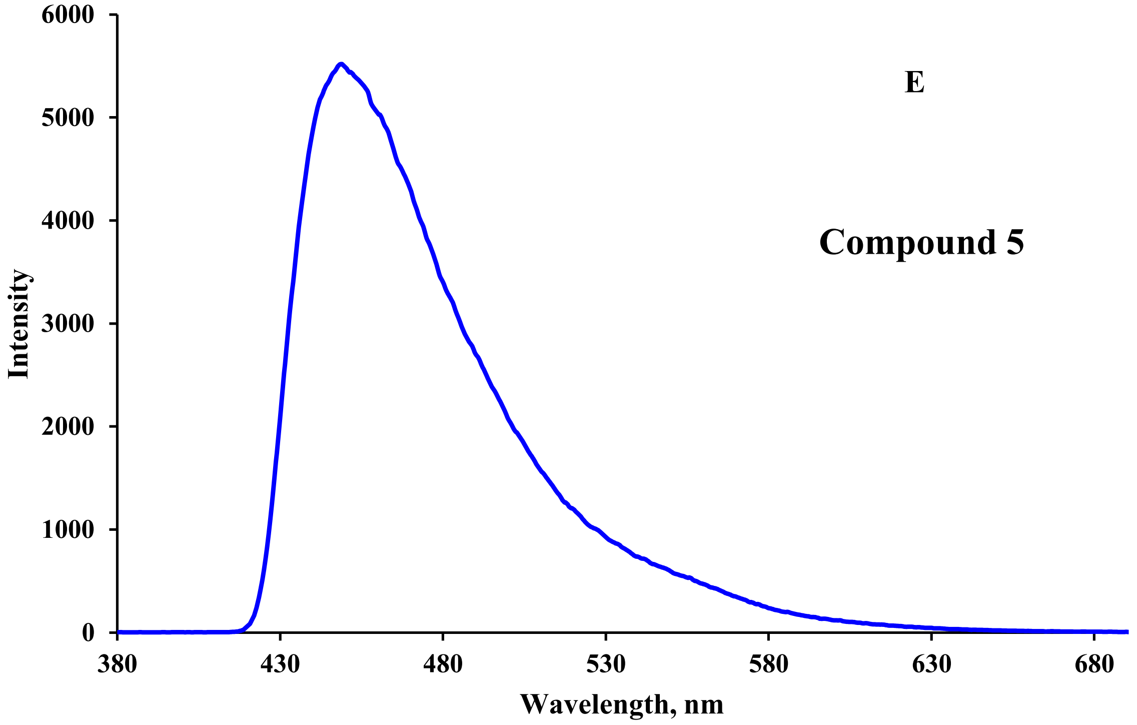

6000
5000
4000
3000
2000
1000
0
Intensity
380
430
480
530
580
630
680
Wavelength, nm
E
Compound 5

Figure S5

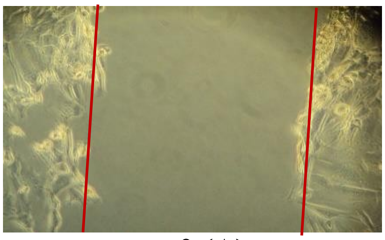

t=0 (A)

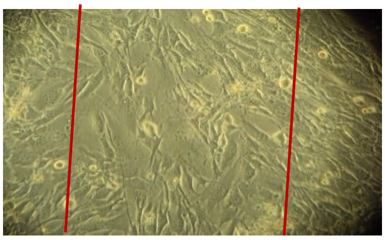

24h (B)

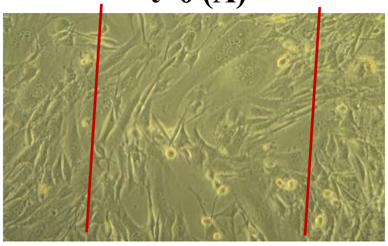

1 (10 µM) (C)

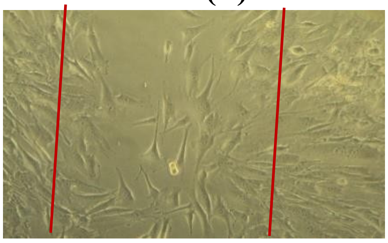

2 (10 µM) (D)

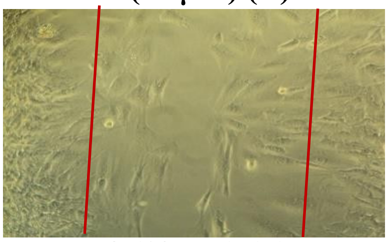

3 (10 µM) (E)

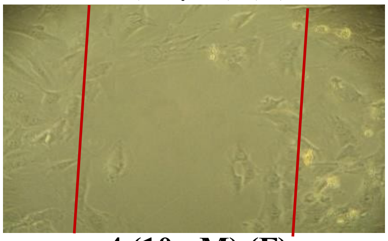

4 (10 µM) (F)

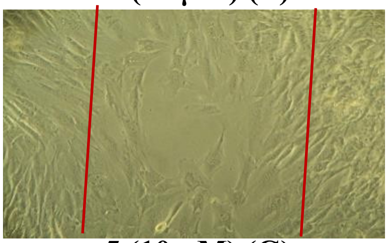

5 (10 µM) (G)

Figure S6

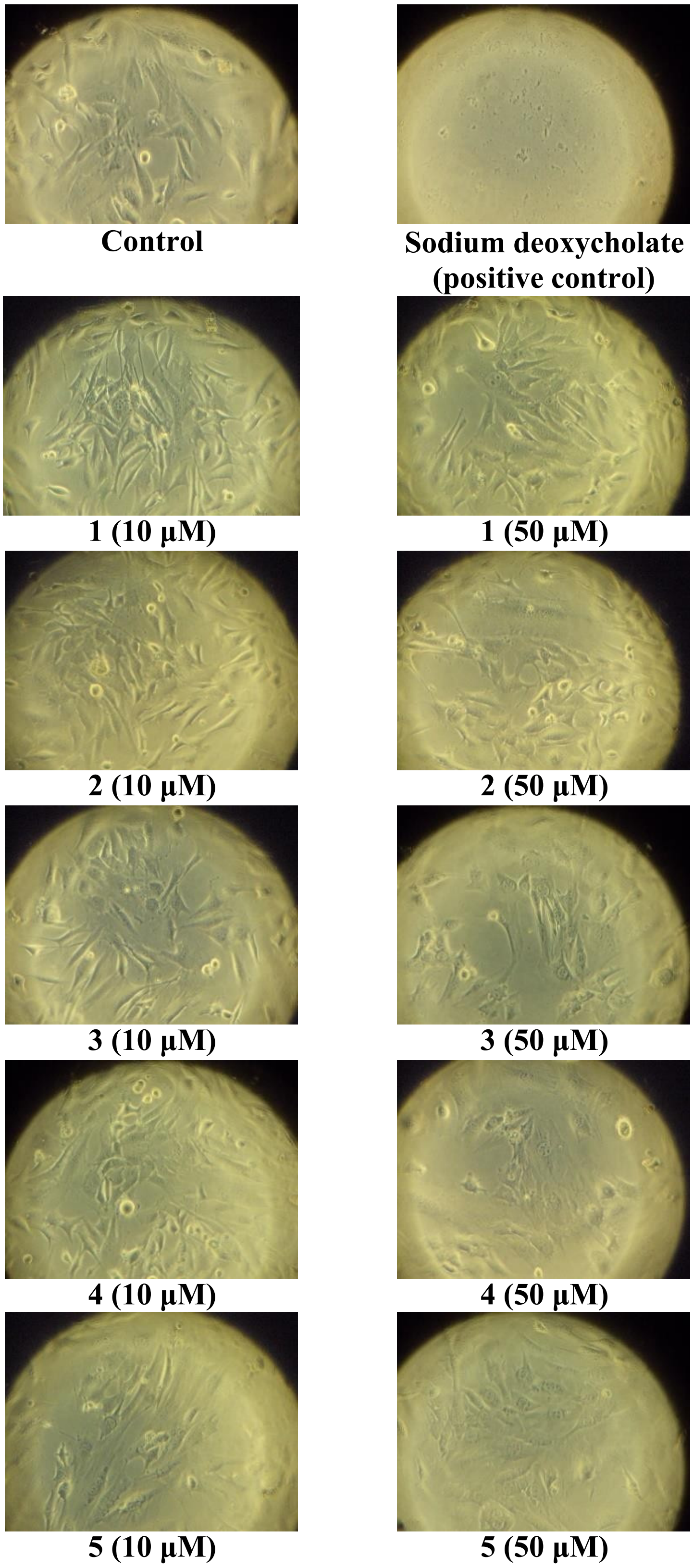
Control
Sodium deoxycholate
(positive control)
1 (10 µM)
1 (50 µM)
2 (10 µM)
2 (50 µM)
3 (10 µM)
3 (50 µM)
4 (10 µM)
4 (50 µM)
5 (10 µM)
5 (50 µM)

Figure S7
