# Supplementary material for: Evaluation of Insulin-Like Activity of Novel Zinc Metal–Organics toward Adipogenesis Signaling
Source: Int J Mol Sci. 2021 Jun 23;22(13):6757. doi: 10.3390/ijms22136757 (PMC8268141; doi:10.3390/ijms22136757)

checkCIF/PLATON report

Structure factors have been supplied for datablock(s) compound2

THIS REPORT IS FOR GUIDANCE ONLY. IF USED AS PART OF A REVIEW PROCEDURE FOR PUBLICATION, IT SHOULD NOT REPLACE THE EXPERTISE OF AN EXPERIENCED CRYSTALLOGRAPHIC REFEREE.

No syntax errors found.   CIF dictionary   Interpreting this report

Datablock: compound2

```
Bond precision:  C-C = 0.0067 A          Wavelength=1.54178

Cell:             a=7.3509(3)     b=9.7341(2)        c=14.3156(3)
                  alpha=90        beta=99.807(1)     gamma=90
Temperature:      160 K

                 Calculated                Reported
Volume           1009.38(5)                1009.38(5)
Space group      P 21/c                    P 21/c
Hall group       -P 2ybc                   -P 2ybc
Moiety formula   C6 H11 N O6 Zn, H2 O      ?
Sum formula      C6 H13 N O7 Zn            C6 H13 N O7 Zn
Mr               276.56                    276.54
Dx,g cm-3        1.820                     1.820
Z                4                         4
Mu (mm-1)        3.622                     3.622
F000             568.0                     568.0
F000'            563.64
h,k,lmax         8,11,16                   8,11,16
Nref             1724                      1694
Tmin,Tmax        0.805,0.834               0.699,1.000
Tmin'            0.170

Correction method= # Reported T Limits: Tmin=0.699 Tmax=1.000
AbsCorr = EMPIRICAL

Data completeness= 0.983          Theta(max)= 64.968

R(reflections)= 0.0556( 1347)     wR2(reflections)= 0.1200( 1694)

S = 1.194                  Npar= 188
```

The following ALERTS were generated. Each ALERT has the format
test-name_ALERT_alert-type_alert-level.
Click on the hyperlinks for more details of the test.

Alert level C

```
THETM01_ALERT_3_C  The value of sine(theta_max)/wavelength is less than 0.590
            Calculated sin(theta_max)/wavelength =    0.5877
PLAT018_ALERT_1_C _diffrn_measured_fraction_theta_max .NE. *_full           ! Check
PLAT088_ALERT_3_C Poor Data / Parameter Ratio ....................       9.01 Note
PLAT222_ALERT_3_C NonSolvent Resd 1  H   Uiso(max)/Uiso(min) Range       10.0 Ratio
PLAT245_ALERT_2_C U(iso) H3B       Smaller than U(eq) C3        by      0.018 Ang**2
PLAT245_ALERT_2_C U(iso) H5B       Smaller than U(eq) C5        by      0.019 Ang**2
PLAT341_ALERT_3_C Low Bond Precision on  C-C Bonds ...............    0.00667 Ang.
PLAT911_ALERT_3_C Missing FCF Refl Between Thmin & STh/L=    0.588         28 Report
PLAT976_ALERT_2_C Check Calcd Resid. Dens.  0.71A   From O1W            -0.41 eA-3
```

Alert level G

```
PLAT004_ALERT_5_G Polymeric Structure Found with Maximum Dimension          1 Info
PLAT164_ALERT_4_G Nr. of Refined C-H H-Atoms in Heavy-Atom Struct.          8 Note
PLAT720_ALERT_4_G Number of Unusual/Non-Standard Labels ..........          4 Note
PLAT794_ALERT_5_G Tentative Bond Valency for Zn        (II)      .       2.02 Info
PLAT883_ALERT_1_G No Info/Value for _atom_sites_solution_primary .     Please Do !
PLAT909_ALERT_3_G Percentage of I>2sig(I) Data at Theta(Max) Still        60% Note
PLAT910_ALERT_3_G Missing # of FCF Reflection(s) Below Theta(Min).          3 Note
PLAT933_ALERT_2_G Number of OMIT Records in Embedded .res File ...         28 Note
PLAT978_ALERT_2_G Number C-C Bonds with Positive Residual Density.          1 Info
```

```
   0 ALERT level A = Most likely a serious problem - resolve or explain
   0 ALERT level B = A potentially serious problem, consider carefully
   9 ALERT level C = Check. Ensure it is not caused by an omission or oversight
   9 ALERT level G = General information/check it is not something unexpected

   2 ALERT type 1 CIF construction/syntax error, inconsistent or missing data
   5 ALERT type 2 Indicator that the structure model may be wrong or deficient
   7 ALERT type 3 Indicator that the structure quality may be low
   2 ALERT type 4 Improvement, methodology, query or suggestion
   2 ALERT type 5 Informative message, check
```

Validation response form

Please find below a validation response form (VRF) that can be filled in and pasted into your CIF.

```
# start Validation Reply Form
_vrf_THETM01_compound2
;
PROBLEM: The value of sine(theta_max)/wavelength is less than 0.590
RESPONSE: ...
;
_vrf_PLAT018_compound2
;
PROBLEM: _diffrn_measured_fraction_theta_max .NE. *_full           ! Check
RESPONSE: ...
;
_vrf_PLAT088_compound2
;
PROBLEM: Poor Data / Parameter Ratio ....................       9.01 Note
RESPONSE: ...
;
_vrf_PLAT222_compound2
;
PROBLEM: NonSolvent Resd 1  H   Uiso(max)/Uiso(min) Range       10.0 Ratio
RESPONSE: ...
```

```
;
_vrf_PLAT245_compound2
;
PROBLEM: U(iso) H3B       Smaller than U(eq) C3        by      0.018 Ang**2
RESPONSE: ...
;
_vrf_PLAT341_compound2
;
PROBLEM: Low Bond Precision on  C-C Bonds ...............    0.00667 Ang.
RESPONSE: ...
;
_vrf_PLAT911_compound2
;
PROBLEM: Missing FCF Refl Between Thmin & STh/L=    0.588         28 Report
RESPONSE: ...
;
_vrf_PLAT976_compound2
;
PROBLEM: Check Calcd Resid. Dens.  0.71A   From O1W            -0.41 eA-3
RESPONSE: ...
;
# end Validation Reply Form
```

It is advisable to attempt to resolve as many as possible of the alerts in all categories. Often the minor alerts point to easily fixed oversights, errors and omissions in your CIF or refinement strategy, so attention to these fine details can be worthwhile. In order to resolve some of the more serious problems it may be necessary to carry out additional measurements or structure refinements. However, the purpose of your study may justify the reported deviations and the more serious of these should normally be commented upon in the discussion or experimental section of a paper or in the "special_details" fields of the CIF. checkCIF was carefully designed to identify outliers and unusual parameters, but every test has its limitations and alerts that are not important in a particular case may appear. Conversely, the absence of alerts does not guarantee there are no aspects of the results needing attention. It is up to the individual to critically assess their own results and, if necessary, seek expert advice.

Publication of your CIF in IUCr journals

A basic structural check has been run on your CIF. These basic checks will be run on all CIFs submitted for publication in IUCr journals (*Acta Crystallographica*, *Journal of Applied Crystallography*, *Journal of Synchrotron Radiation*); however, if you intend to submit to *Acta Crystallographica Section C* or *E* or *IUCrData*, you should make sure that full publication checks are run on the final version of your CIF prior to submission.

Publication of your CIF in other journals

Please refer to the *Notes for Authors* of the relevant journal for any special instructions relating to CIF submission.

PLATON version of 22/03/2021; check.def file version of 19/03/2021

Datablock compound2 - ellipsoid plot

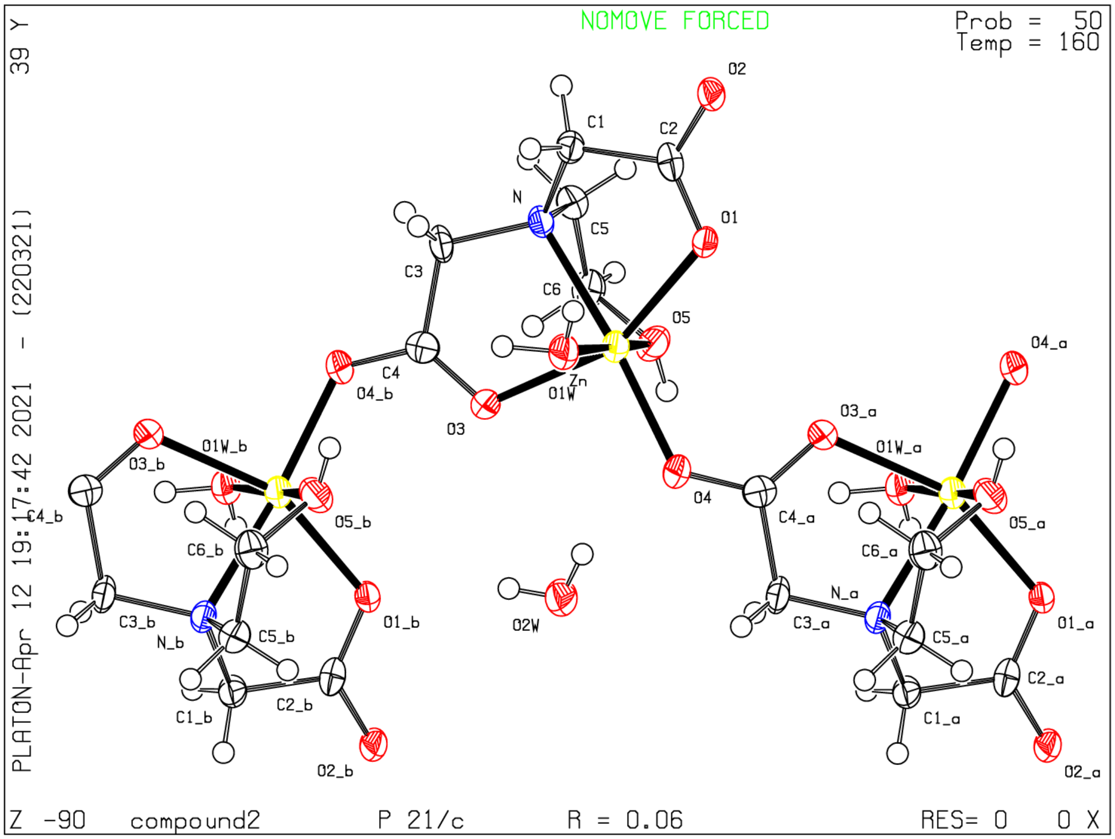

Supplement: Supplementary file 1 [file ijms-22-06757-s001.zip › ijms-1239064 supplementary - Final cifs and checkcifs/Compound 2/Compound2checkcif.pdf]
