# Supplementary material for: Evaluation of Insulin-Like Activity of Novel Zinc Metal–Organics toward Adipogenesis Signaling
Source: Int J Mol Sci. 2021 Jun 23;22(13):6757. doi: 10.3390/ijms22136757 (PMC8268141; doi:10.3390/ijms22136757)

checkCIF/PLATON report

Structure factors have been supplied for datablock(s) compound5

THIS REPORT IS FOR GUIDANCE ONLY. IF USED AS PART OF A REVIEW PROCEDURE FOR PUBLICATION, IT SHOULD NOT REPLACE THE EXPERTISE OF AN EXPERIENCED CRYSTALLOGRAPHIC REFEREE.

No syntax errors found. CIF dictionary Interpreting this report

Datablock: compound5

```
Bond precision:  C-C = 0.0034 A          Wavelength=1.54178

Cell:            a=10.8589(2)     b=15.4397(2)       c=13.1342(2)
                 alpha=90         beta=93.997(1)     gamma=90
Temperature:     160 K
```

	Calculated	Reported
Volume	2196.70(6)	2196.70(6)
Space group	P 21/n	P 21/n
Hall group	-P 2yn	-P 2yn
Moiety formula	C24 H16 Cl N4 Zn, N O3, H2 O	?
Sum formula	C24 H18 Cl N5 O4 Zn	C24 H19.50 Cl N5 O4 Zn
Mr	541.27	542.76
Dx,g cm-3	1.637	1.641
Z	4	4
Mu (mm-1)	3.056	3.056
F000	1104.0	1110.0
F000’	1102.18	
h,k,lmax	12,18,15	12,17,15
Nref	3748	3732
Tmin,Tmax	0.258,0.343	0.684,1.000
Tmin’	0.110	

```
Correction method= # Reported T Limits: Tmin=0.684 Tmax=1.000
AbsCorr = EMPIRICAL

Data completeness= 0.996          Theta(max)= 64.995

R(reflections)= 0.0350( 3535)     wR2(reflections)= 0.0925( 3732)

S = 1.060                   Npar= 388
```

The following ALERTS were generated. Each ALERT has the format
test-name_ALERT_alert-type_alert-level.
Click on the hyperlinks for more details of the test.

Alert level C

```
THETM01_ALERT_3_C  The value of sine(theta_max)/wavelength is less than 0.590
            Calculated sin(theta_max)/wavelength =    0.5878
PLAT018_ALERT_1_C _diffrn_measured_fraction_theta_max .NE. *_full           ! Check
PLAT041_ALERT_1_C Calc. and Reported SumFormula    Strings  Differ     Please Check
PLAT043_ALERT_1_C Calculated and Reported Mol. Weight Differ by ..       1.49 Check
PLAT068_ALERT_1_C Reported F000 Differs from Calcd (or Missing)...     Please Check
PLAT088_ALERT_3_C Poor Data / Parameter Ratio ....................       9.62 Note
PLAT230_ALERT_2_C Hirshfeld Test Diff for    C6       --C7       .        5.8 s.u.
PLAT244_ALERT_4_C Low    'Solvent' Ueq as Compared to Neighbors of        N31 Check
PLAT354_ALERT_3_C Short  O-H (X0.82,N0.98A)  O1W      - H2W1     .       0.71 Ang.
PLAT906_ALERT_3_C Large K Value in the Analysis of Variance ......      2.223 Check
PLAT910_ALERT_3_C Missing # of FCF Reflection(s) Below Theta(Min).          7 Note
PLAT911_ALERT_3_C Missing FCF Refl Between Thmin & STh/L=    0.588          5 Report
```

Alert level G

```
FORMU01_ALERT_2_G  There is a discrepancy between the atom counts in the
            _chemical_formula_sum and the formula from the _atom_site* data.
            Atom count from _chemical_formula_sum:C24 H19.5 Cl1 N5 O4 Zn1
            Atom count from the _atom_site data:  C24 H18 Cl1 N5 O4 Zn1
CELLZ01_ALERT_1_G Difference between formula and atom_site contents detected.
CELLZ01_ALERT_1_G WARNING: H atoms missing from atom site list. Is this intentional?
           From the CIF: _cell_formula_units_Z    4
           From the CIF: _chemical_formula_sum  C24 H19.50 Cl N5 O4 Zn
           TEST: Compare cell contents of formula and atom_site data

           atom    Z*formula  cif sites diff
           C         96.00     96.00    0.00
           H         78.00     72.00    6.00
           Cl         4.00      4.00    0.00
           N         20.00     20.00    0.00
           O         16.00     16.00    0.00
           Zn         4.00      4.00    0.00
PLAT063_ALERT_4_G Crystal Size Possibly too Large for Beam Size ..       0.65 mm
PLAT164_ALERT_4_G Nr. of Refined C-H H-Atoms in Heavy-Atom Struct.         16 Note
PLAT720_ALERT_4_G Number of Unusual/Non-Standard Labels ..........          2 Note
PLAT794_ALERT_5_G Tentative Bond Valency for Zn        (II)      .       1.84 Info
PLAT883_ALERT_1_G No Info/Value for _atom_sites_solution_primary .     Please Do !
PLAT909_ALERT_3_G Percentage of I>2sig(I) Data at Theta(Max) Still        86% Note
PLAT933_ALERT_2_G Number of OMIT Records in Embedded .res File ...          3 Note
PLAT978_ALERT_2_G Number C-C Bonds with Positive Residual Density.          6 Info
```

---

0 **ALERT level A** = Most likely a serious problem - resolve or explain
0 **ALERT level B** = A potentially serious problem, consider carefully
12 **ALERT level C** = Check. Ensure it is not caused by an omission or oversight
11 **ALERT level G** = General information/check it is not something unexpected

7 ALERT type 1 CIF construction/syntax error, inconsistent or missing data
4 ALERT type 2 Indicator that the structure model may be wrong or deficient
7 ALERT type 3 Indicator that the structure quality may be low
4 ALERT type 4 Improvement, methodology, query or suggestion
1 ALERT type 5 Informative message, check

Validation response form

Please find below a validation response form (VRF) that can be filled in and pasted into your CIF.

```
# start Validation Reply Form
_vrf_THETM01_compound5
;
PROBLEM: The value of sine(theta_max)/wavelength is less than 0.590
RESPONSE: ...
;
_vrf_PLAT018_compound5
;
PROBLEM: _diffrn_measured_fraction_theta_max .NE. *_full           ! Check
RESPONSE: ...
;
_vrf_PLAT041_compound5
;
PROBLEM: Calc. and Reported SumFormula    Strings  Differ     Please Check
RESPONSE: ...
;
_vrf_PLAT043_compound5
;
PROBLEM: Calculated and Reported Mol. Weight Differ by ..       1.49 Check
RESPONSE: ...
;
_vrf_PLAT068_compound5
;
PROBLEM: Reported F000 Differs from Calcd (or Missing)...     Please Check
RESPONSE: ...
;
_vrf_PLAT088_compound5
;
PROBLEM: Poor Data / Parameter Ratio ....................       9.62 Note
RESPONSE: ...
;
_vrf_PLAT230_compound5
;
PROBLEM: Hirshfeld Test Diff for    C6       --C7       .        5.8 s.u.
RESPONSE: ...
;
_vrf_PLAT244_compound5
;
PROBLEM: Low    'Solvent' Ueq as Compared to Neighbors of        N31 Check
RESPONSE: ...
;
_vrf_PLAT354_compound5
;
PROBLEM: Short  O-H (X0.82,N0.98A)  O1W      - H2W1     .       0.71 Ang.
RESPONSE: ...
;
_vrf_PLAT906_compound5
;
PROBLEM: Large K Value in the Analysis of Variance ......      2.223 Check
RESPONSE: ...
;
_vrf_PLAT910_compound5
;
PROBLEM: Missing # of FCF Reflection(s) Below Theta(Min).          7 Note
RESPONSE: ...
;
_vrf_PLAT911_compound5
```

```
;
PROBLEM: Missing FCF Refl Between Thmin & STh/L=    0.588          5 Report
RESPONSE: ...
;
# end Validation Reply Form
```

It is advisable to attempt to resolve as many as possible of the alerts in all categories. Often the minor alerts point to easily fixed oversights, errors and omissions in your CIF or refinement strategy, so attention to these fine details can be worthwhile. In order to resolve some of the more serious problems it may be necessary to carry out additional measurements or structure refinements. However, the purpose of your study may justify the reported deviations and the more serious of these should normally be commented upon in the discussion or experimental section of a paper or in the "special_details" fields of the CIF. checkCIF was carefully designed to identify outliers and unusual parameters, but every test has its limitations and alerts that are not important in a particular case may appear. Conversely, the absence of alerts does not guarantee there are no aspects of the results needing attention. It is up to the individual to critically assess their own results and, if necessary, seek expert advice.

Publication of your CIF in IUCr journals

A basic structural check has been run on your CIF. These basic checks will be run on all CIFs submitted for publication in IUCr journals (*Acta Crystallographica*, *Journal of Applied Crystallography*, *Journal of Synchrotron Radiation*); however, if you intend to submit to *Acta Crystallographica Section C* or *E* or *IUCrData*, you should make sure that full publication checks are run on the final version of your CIF prior to submission.

Publication of your CIF in other journals

Please refer to the *Notes for Authors* of the relevant journal for any special instructions relating to CIF submission.

PLATON version of 22/03/2021; check.def file version of 19/03/2021

Datablock compound5 - ellipsoid plot

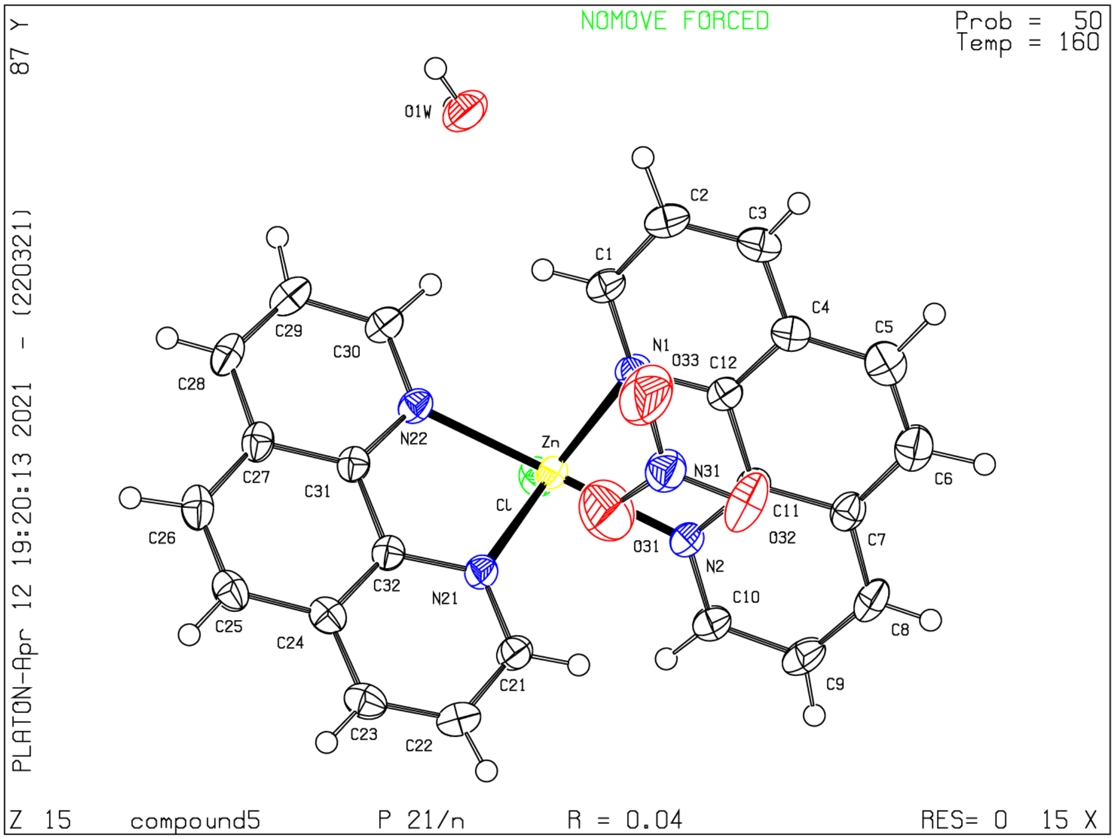

Supplement: Supplementary file 1 [file ijms-22-06757-s001.zip › ijms-1239064 supplementary - Final cifs and checkcifs/Compound 5/Compound5checkcif.pdf]
