# Supplementary material for: Evaluation of Insulin-Like Activity of Novel Zinc Metal–Organics toward Adipogenesis Signaling
Source: Int J Mol Sci. 2021 Jun 23;22(13):6757. doi: 10.3390/ijms22136757 (PMC8268141; doi:10.3390/ijms22136757)

checkCIF/PLATON report

Structure factors have been supplied for datablock(s) compound1

THIS REPORT IS FOR GUIDANCE ONLY. IF USED AS PART OF A REVIEW PROCEDURE FOR PUBLICATION, IT SHOULD NOT REPLACE THE EXPERTISE OF AN EXPERIENCED CRYSTALLOGRAPHIC REFEREE.

No syntax errors found. CIF dictionary Interpreting this report

Datablock: compound1

```
Bond precision:  C-C = 0.0057 A          Wavelength=1.54178

Cell:          a=7.2582(1)       b=8.1539(1)      c=13.8300(3)
               alpha=83.107(1)   beta=84.445(1)   gamma=75.707(1)
Temperature:   160 K

                Calculated                 Reported
Volume          785.51(2)                  785.51(2)
Space group     P -1                       P -1
Hall group      -P 1                       -P 1
Moiety formula  C14 H14 Cl2 N2 O4 Zn       ?
Sum formula     C14 H14 Cl2 N2 O4 Zn       C14 H14 Cl2 N2 O4 Zn
Mr              410.56                     410.54
Dx,g cm-3       1.736                      1.736
Z               2                          2
Mu (mm-1)       5.500                      5.500
F000            416.0                      416.0
F000'           415.33
h,k,lmax        8,9,16                     8,9,16
Nref            2669                       2408
Tmin,Tmax       0.170,0.298                0.619,1.000
Tmin'           0.050

Correction method= # Reported T Limits: Tmin=0.619 Tmax=1.000
AbsCorr = EMPIRICAL

Data completeness= 0.902          Theta(max)= 64.986

R(reflections)= 0.0362( 2016)     wR2(reflections)= 0.0908( 2408)

S = 1.188                  Npar= 212
```

```
The following ALERTS were generated. Each ALERT has the format
       test-name_ALERT_alert-type_alert-level.
Click on the hyperlinks for more details of the test.
```

Alert level B

```
PLAT911_ALERT_3_B Missing FCF Refl Between Thmin & STh/L=    0.588        256 Report
```

Author Response: This is probably due to instrument limitation for Cu radiation and small unit cells

Alert level C

```
THETM01_ALERT_3_C  The value of sine(theta_max)/wavelength is less than 0.590
            Calculated sin(theta_max)/wavelength =    0.5878
PLAT018_ALERT_1_C _diffrn_measured_fraction_theta_max .NE. *_full           ! Check
PLAT222_ALERT_3_C NonSolvent Resd 1  H   Uiso(max)/Uiso(min) Range        4.9 Ratio
PLAT790_ALERT_4_C Centre of Gravity not Within Unit Cell: Resd.  #          1 Note
              C14 H14 Cl2 N2 O4 Zn
PLAT910_ALERT_3_C Missing # of FCF Reflection(s) Below Theta(Min).          5 Note
PLAT977_ALERT_2_C Check Negative Difference Density on H7A              -0.33 eA-3
PLAT977_ALERT_2_C Check Negative Difference Density on H7C              -0.43 eA-3
PLAT977_ALERT_2_C Check Negative Difference Density on H17B             -0.33 eA-3
PLAT977_ALERT_2_C Check Negative Difference Density on H17C             -0.32 eA-3
```

Alert level G

```
PLAT093_ALERT_1_G No s.u.'s on H-positions, Refinement Reported as      mixed Check
PLAT154_ALERT_1_G The s.u.'s on the Cell Angles are Equal ..(Note)      0.001 Degree
PLAT380_ALERT_4_G Incorrectly? Oriented X(sp2)-Methyl Moiety .....         C7 Check
PLAT380_ALERT_4_G Incorrectly? Oriented X(sp2)-Methyl Moiety .....        C17 Check
PLAT794_ALERT_5_G Tentative Bond Valency for Zn        (II)      .       2.00 Info
PLAT883_ALERT_1_G No Info/Value for _atom_sites_solution_primary .     Please Do !
PLAT909_ALERT_3_G Percentage of I>2sig(I) Data at Theta(Max) Still        66% Note
PLAT933_ALERT_2_G Number of OMIT Records in Embedded .res File ...         18 Note
PLAT941_ALERT_3_G Average HKL Measurement Multiplicity ...........        4.2 Low
PLAT978_ALERT_2_G Number C-C Bonds with Positive Residual Density.          1 Info
```

```
   0 ALERT level A = Most likely a serious problem - resolve or explain
   1 ALERT level B = A potentially serious problem, consider carefully
   9 ALERT level C = Check. Ensure it is not caused by an omission or oversight
  10 ALERT level G = General information/check it is not something unexpected

   4 ALERT type 1 CIF construction/syntax error, inconsistent or missing data
   6 ALERT type 2 Indicator that the structure model may be wrong or deficient
   6 ALERT type 3 Indicator that the structure quality may be low
   3 ALERT type 4 Improvement, methodology, query or suggestion
   1 ALERT type 5 Informative message, check
```

Validation response form

Please find below a validation response form (VRF) that can be filled in and pasted into your CIF.

```
# start Validation Reply Form
_vrf_THETM01_compound1
;
PROBLEM: The value of sine(theta_max)/wavelength is less than 0.590
RESPONSE: ...
;
_vrf_PLAT018_compound1
;
PROBLEM: _diffrn_measured_fraction_theta_max .NE. *_full           ! Check
RESPONSE: ...
;
_vrf_PLAT222_compound1
;
PROBLEM: NonSolvent Resd 1  H   Uiso(max)/Uiso(min) Range        4.9 Ratio
RESPONSE: ...
;
_vrf_PLAT790_compound1
;
PROBLEM: Centre of Gravity not Within Unit Cell: Resd.  #          1 Note
RESPONSE: ...
;
_vrf_PLAT910_compound1
;
PROBLEM: Missing # of FCF Reflection(s) Below Theta(Min).          5 Note
RESPONSE: ...
;
_vrf_PLAT977_compound1
;
PROBLEM: Check Negative Difference Density on H7A              -0.33 eA-3
RESPONSE: ...
;
# end Validation Reply Form
```

It is advisable to attempt to resolve as many as possible of the alerts in all categories. Often the minor alerts point to easily fixed oversights, errors and omissions in your CIF or refinement strategy, so attention to these fine details can be worthwhile. In order to resolve some of the more serious problems it may be necessary to carry out additional measurements or structure refinements. However, the purpose of your study may justify the reported deviations and the more serious of these should normally be commented upon in the discussion or experimental section of a paper or in the "special_details" fields of the CIF. checkCIF was carefully designed to identify outliers and unusual parameters, but every test has its limitations and alerts that are not important in a particular case may appear. Conversely, the absence of alerts does not guarantee there are no aspects of the results needing attention. It is up to the individual to critically assess their own results and, if necessary, seek expert advice.

Publication of your CIF in IUCr journals

A basic structural check has been run on your CIF. These basic checks will be run on all CIFs submitted for publication in IUCr journals (*Acta Crystallographica*, *Journal of Applied Crystallography*, *Journal of Synchrotron Radiation*); however, if you intend to submit to *Acta Crystallographica Section C* or *E* or *IUCrData*, you should make sure that full publication checks are run on the final version of your CIF prior to submission.

Publication of your CIF in other journals

Please refer to the *Notes for Authors* of the relevant journal for any special instructions relating to CIF submission.

PLATON version of 22/03/2021; check.def file version of 19/03/2021

Datablock compound1 - ellipsoid plot

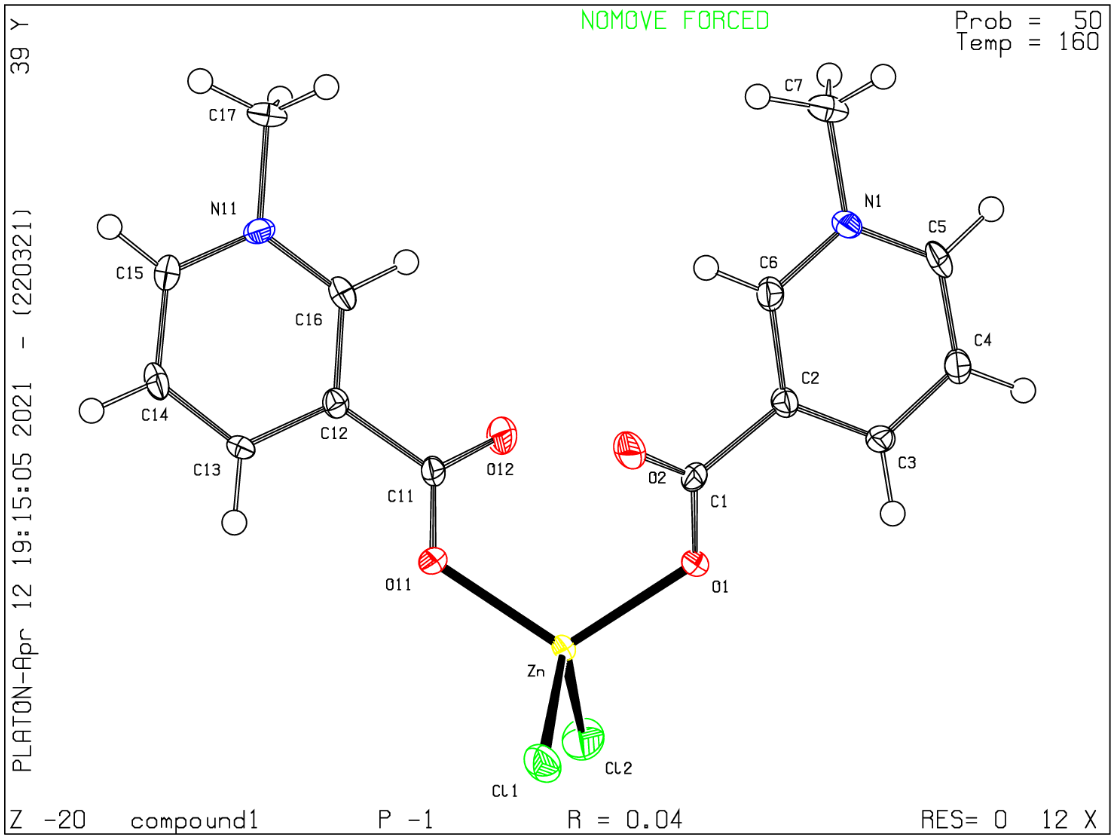

Supplement: Supplementary file 1 [file Compound1checkcif.pdf › ijms-1239064 supplementary - Final cifs and checkcifs/Compound 1/Compound1checkcif.pdf]
